# Design, Synthesis, and Biological Activity Evaluation of Quinazoline-Based PLK4 Inhibitors

**DOI:** 10.3390/biomedicines14081811

**Published:** 2026-08-12

**Authors:** Wenqiang Sun, Zehui Qi, Ningyuan Hu, Nian Liu, Shuyi Mu, Haoyu Zhang, Zixuan Gao, Zirui Luo, Yin Sun, Dongmei Zhao, Maosheng Cheng

**Affiliations:** Key Laboratory of Structure-Based Drug Design and Discovery, Ministry of Education, School of Pharmaceutical Engineering, Shenyang Pharmaceutical University, 103 Wenhua Road, Shenhe District, Shenyang 110016, Chinayinsun@stanford.edu (Y.S.);

**Keywords:** PLK4 inhibitor, quinazoline, anti-tumor

## Abstract

**Background:** As a key regulator of centrosome duplication, polo-like kinase 4 (PLK4) is abnormally overexpressed in various tumors and has emerged as an important target for the development of antitumor drugs. **Methods:** In this study, based on the lead compound **ZSL-M001** (PLK4 IC50 = 3.0 μM) previously obtained by our research group, we designed and synthesized 28 novel quinazoline-based PLK4 inhibitors to enhance kinase inhibitory activity using side-chain extension strategies. **Results:** Among them, compound **H24** (PLK4 IC50 < 0.1 nM) exhibited favorable in vitro antiproliferative activity against *TRIM37*-amplified MCF-7 breast cancer cells (MCF-7 IC50 = 2.37 ± 0.26 μM) and *TRIM37*-amplified neuroblastoma IMR-32 cells (IMR-32 IC50 = 1.28 ± 0.04 μM). Its activity was superior to that of the positive control LCR-263. Further in vitro biological evaluation demonstrated that **H24** inhibited the colony formation of MCF-7 cells in a concentration-dependent manner, induced S/G2-phase cell-cycle arrest, and promoted apoptosis. **H24** also showed favorable metabolic stability in human liver microsomes, with a half-life of 61.3 min. However, no obvious *TRIM37* amplification-dependent cellular selectivity was observed in *TRIM37* non-highly amplified A549 cells or normal human embryonic kidney HEK-293T cells. Given the suboptimal selectivity of the in vitro antiproliferative activity, Aurora kinase A, which shares high homology with PLK4, was selected for further evaluation of its selectivity. The IC50 for Aurora A inhibition was determined to be 151.2 nM, indicating that its broader kinase selectivity remains to be established. **Conclusions:** In summary, **H24** is a highly potent biochemical PLK4 inhibitor and provides a useful lead scaffold for further optimization rather than a fully selective cellular candidate at the current stage.

## 1. Introduction

Mitosis is the fundamental process of replication and proliferation in eukaryotic cells. The organelle responsible for regulating the accuracy of mitosis is the centrosome, and the development of tumors is often associated with mitotic dysregulation caused by centrosomal abnormalities [1,2]. The polo-like kinase (PLK) family is a subgroup of serine/threonine kinases that regulates various mitotic processes, including entry into and exit from mitosis, centrosome replication, chromosome segregation, and the DNA damage response (DDR) [3,4]. As important members of the serine/threonine protein kinase family, PLKs comprise five members (PLK1–PLK5), all of which possess a highly conserved N-terminal serine/threonine kinase domain and a C-terminal regulatory region [5,6,7]. The kinase domain of PLK4 exhibits unique structural differences compared to other family members, making it a distinct member of the PLK family [8,9,10,11]. For most PLK family members, the C-terminal region comprises two conserved polo-box domains (PBDs) that coalesce to form a PBD structure essential for subcellular targeting and kinase regulation [12]. In contrast, PLK4 is characterized by a single PBD and a cryptic polo-box (CPB) region. This region is followed by a coiled-coil domain and a PB3 domain. The PB3 domain of PLK4 interacts with the coiled-coil domain of the SCL-interacting locus protein (STIL), which is critical for the initiation of centriole replication [13,14].

As a key regulatory kinase in the centrosome replication process, overexpression of PLK4 can induce abnormal centrosome proliferation, leading to genomic instability and promoting tumorigenesis [15]. PLK4 has been identified as one of the key drivers of tumorigenesis and is overexpressed in various human tumors, including colorectal cancer, breast cancer, and neuroblastoma [16,17,18,19,20,21]. Consequently, PLK4 has emerged as an important target for precision cancer therapy. To date, several PLK4 inhibitors have been developed and have demonstrated significant antitumor activity across various cancer types [22].

**Figure 1:**
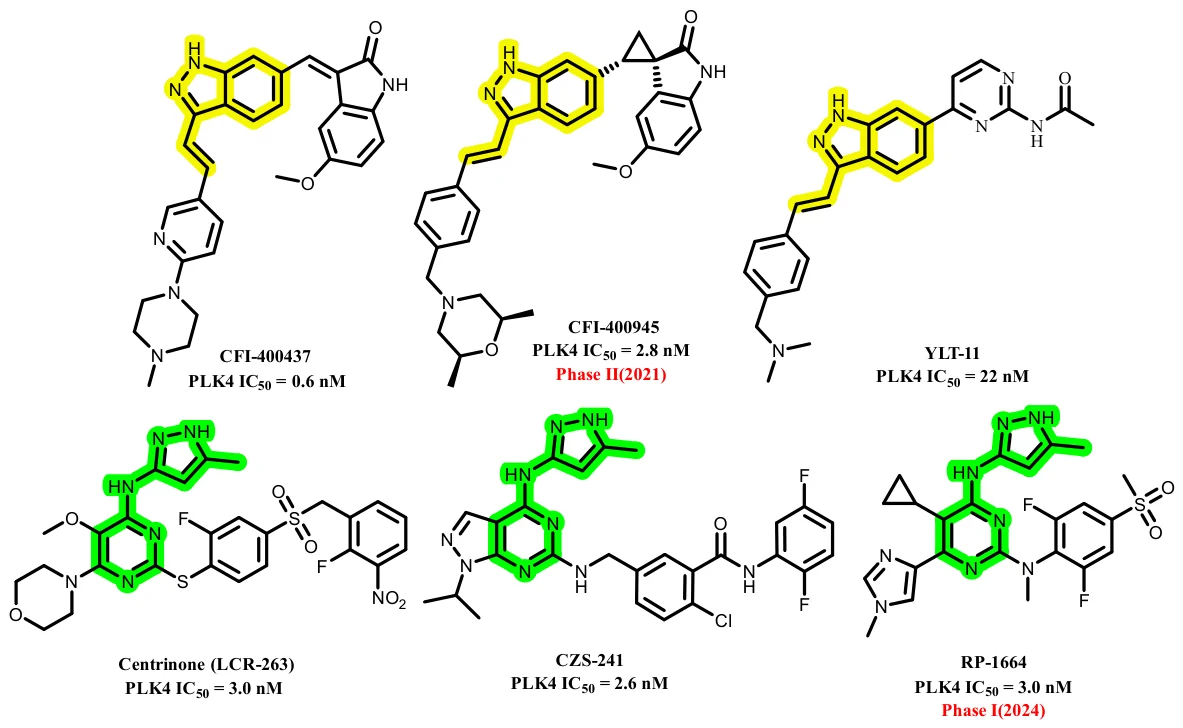
Representative PLK4 inhibitors (Yellow represents the indazole scaffold, and green represents the aminopyrazole scaffold).

From a structural perspective, reported PLK4 inhibitors can be broadly classified into two major categories: indazoles and aminopyrazoles (Figure 1). Pauls et al. [23] identified a series of novel indazole-based PLK4 inhibitors, among which **CFI-400437** exhibited superior PLK4 kinase inhibitory activity, with an IC_50_ of 0.6 nM. Subsequently, the research team further modified the structure of **CFI-400437** to develop **CFI-400945**, with an IC_50_ of 2.8 nM. **CFI-400945** is now in Phase II clinical trials for the treatment of breast cancers and acute myeloid leukemia (AML) and chronic myelomonocytic leukemia (CMML) [24]. Yu’s team conducted further structural modifications on **CFI-400945**, resulting in the novel indazole-based PLK4 inhibitor **YLT-11** (IC_50_ = 22 nM), which significantly inhibits tumor growth in MCF-7, MDA-MB-231, and MDA-MB-468 mouse xenograft models [25,26]. Centrinone (**LCR-263**) is a representative aminopyrazole-based PLK4 inhibitor (IC_50_ = 2.7 nM). However, issues such as its short half-life and poor safety profile have limited its further development [27,28,29,30]. **CZS-241** is another highly promising aminopyrazole-based PLK4 inhibitor discovered by our research group [31], exhibiting potent inhibitory activity against PLK4 with an IC_50_ value of 2.6 nM. Compound **RP-1664** (PLK4 IC_50_ = 3.0 nM), derived from the structure of Centrinone B (**LCR-263** analogs), entered Phase I clinical trials in February 2024 [32]. However, no PLK4 inhibitor has yet been approved for clinical use. Therefore, the development of novel PLK4 inhibitors with favorable safety profiles and therapeutic potential holds important scientific significance and application value.

**Figure 2:**
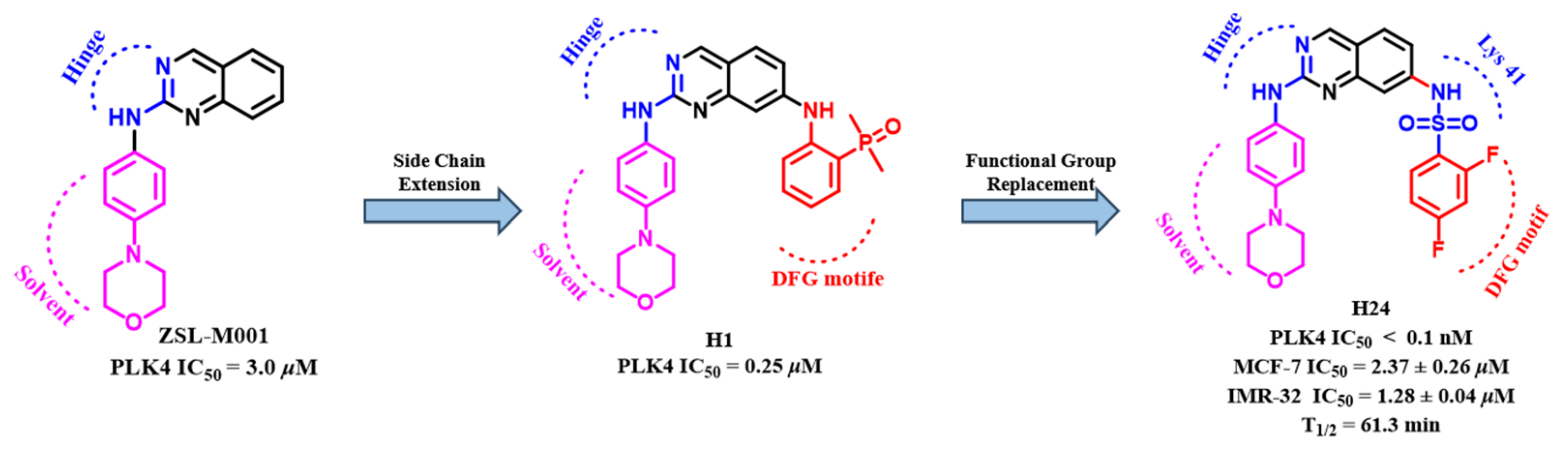
Design and modification of PLK4 inhibitor types.

In our previous study, the quinazoline-based compound **ZSL-M001** [33] was identified as having PLK4 kinase inhibitory activity (IC_50_ = 3.0 μM) and was considered a promising scaffold worthy of further optimization. Based on rational drug design principles, the left-side hydrophilic pharmacophore (4-morpholinylaniline moiety) was retained, and a side-chain extension strategy was employed to modify the hydrophobic fragment oriented toward the DFG-motif. Subsequently, guided by a rational drug design strategy, the original aniline moiety was transformed into a benzenesulfonamide scaffold, with appropriate hydrophobic groups being introduced onto the phenyl ring to occupy the hydrophobic pocket and enhance binding affinity. Ultimately, compound **H24** was obtained, which exhibited remarkably potent biochemical PLK4 inhibitory activity with an IC_50_ value below 0.1 nM. In vitro cellular assays confirmed that **H24** exerted favorable antiproliferative effects on neuroblastoma IMR-32 cells (IC_50_ = 1.28 ± 0.04 μM) and breast cancer MCF-7 cells (IC_50_ = 2.37 ± 0.26 μM) (Figure 2). However, unsatisfactory cellular selectivity was observed for **H24**, along with a notable discrepancy between its potent PLK4 inhibition and cellular antiproliferative potency. Taken together, **H24** is recognized as an excellent lead compound, yet further structural optimization is warranted to overcome its poor selectivity and the mismatch between kinase inhibition and cellular activity.

## 2. Experimental Section

### 2.1. Synthesis Experiments

Unless otherwise specified, all starting materials and reagents used for synthesis were commercially available and used directly without further purification. All reactions sensitive to air or moisture were carried out under an anhydrous argon (Ar) atmosphere. Thin-layer chromatography (TLC) was employed to monitor the reaction progress, and the plates were visualized under UV light (365 nm or 254 nm). Preparative thin-layer chromatography was routinely used for the isolation of target compounds on HSGF-254 (0.20–0.25 mm) plates. The structures of the target compounds were characterized by melting point determination, nuclear magnetic resonance (NMR) spectroscopy, and high-resolution mass spectrometry (HRMS). Melting points were measured on a Büchi B-540 melting point apparatus. NMR spectra were recorded on a Bruker ARX-400 or ARX-600 spectrometer (Bruker Biospin, Karlsruhe, Germany) using DMSO-*d*_6_ as the solvent with tetramethylsilane (TMS) as the internal standard. HRMS data were obtained on a Bruker Micromass mass spectrometer (Bruker Daltonik GmbH, Bremen, Germany). The purity of all target compounds was determined using a Gilson 215 high-performance liquid chromatography (HPLC) system equipped with a YMC ODS3 column (50 mm × 4.6 mm, 5 μm particle size, YMC Co., Ltd., Kyoto, Japan), with the UV detection wavelength set at 254 nm. All synthesized products were confirmed to have a purity higher than 95%. The mobile phase consisted of Eluent A (water) and Eluent B (methanol) using a gradient elution from 20% to 80% B over 15–30 min at a flow rate of 1.0 mL/min. The full sets of ^1^H NMR, ^13^C NMR spectra and HPLC purity profiles of the synthesized target compounds are provided in the Supplementary Materials, which serve as supporting documentation for structural assignment and confirm the purity of all samples for subsequent biological activity assays.

#### 2.1.1. Synthesis of Intermediates

Intermediate 2-amino-4-bromobenzaldehyde (**M2**) [34]

Starting material **M1** (10.0 g, 43.50 mmol, 1.0 equiv) was dissolved in 100.0 mL of a mixed solvent of ethanol/water (4:1, *v*/*v*). Iron powder (24.3 g, 0.44 mol, 10.0 equiv) and concentrated hydrochloric acid (0.4 mL, 4.35 mmol, 0.1 equiv, 12 M) were added sequentially. The reaction mixture was stirred at room temperature for 6 h under an Ar atmosphere. The reaction progress was monitored by TLC using a developing agent of petroleum ether/ethyl acetate (PE: EA = 4:1, *v*/*v*) until complete consumption of the starting material was confirmed. The reaction mixture was filtered through Celite to remove insoluble residues, and the filtrate was extracted with ethyl acetate (EA). The combined organic layers were washed with saturated sodium chloride solution and dried over anhydrous sodium sulfate (Na_2_SO_4_). The organic phase was concentrated under reduced pressure to remove the solvent, and the residue was dried at 60 °C to afford compound **M2** as a white powdery solid (9.1 g, 96.8% yield). m.p. 85.1–85.3 °C. ^1^H NMR (600 MHz, DMSO-*d*6) δ 9.80 (s, 1H), 7.49 (d, *J* = 8.3 Hz, 1H), 7.27 (s, 2H), 7.00 (d, *J* = 1.8 Hz, 1H), 6.79 (dd, *J* = 8.3, 1.9 Hz, 1H).

Intermediate 7-bromoquinazolin-2-ol (**M3**)

Intermediate **M2** (9.1 g, 45.70 mmol, 1.0 equiv) and urea (41.2 g, 0.69 mol, 15.0 equiv) were mixed and heated at 180 °C for 5 h. The reaction progress was monitored by TLC using a developing agent of petroleum ether/ethyl acetate (PE:EA = 6:1, *v*/*v*) until complete consumption of the starting material was confirmed. After cooling to room temperature naturally, 500 mL of distilled water was added to the reaction mixture, which was then stirred at 100 °C for 1 h. Upon cooling to room temperature, the mixture was filtered by suction. The filter cake was dried at 60 °C to afford intermediate **M3** as a yellow powdery solid (10.1 g, 94.5% yield).

Intermediate 7-bromo-2-chloroquinazoline (**M4**) [35]

Intermediate **M3** (10.1 g, 45.10 mmol, 1.0 equiv) was dissolved in POCl_3_ (105.0 mL, 1.13 mol, 25.0 equiv), and the mixture was heated to reflux at 110 °C for 5 h. The reaction progress was monitored by TLC using a developing agent of petroleum ether/ethyl acetate (PE: EA = 6:1, *v*/*v*) until complete consumption of the starting material was confirmed. After cooling to room temperature naturally, the reaction mixture was slowly poured into ice water (400 mL) under vigorous stirring to quench the reaction. The resulting mixture was filtered by suction to afford the crude product as a filter cake. The crude product was purified by column chromatography using a gradient elution of petroleum ether/ethyl acetate (*v*/*v*, ranging from 80:1 to 20:1). The solvent was removed under reduced pressure, and the residue was dried at 60 °C to afford intermediate **M4** as a white powdery solid (3.5 g, 47.3% yield). m.p. 165.2–166.0 °C. ^1^H NMR (600 MHz, DMSO-*d*_6_) δ 9.64 (s, 1H), 8.27 (d, *J* = 1.8 Hz, 1H), 8.20 (d, *J* = 8.7 Hz, 1H), 7.98 (dd, *J* = 8.6, 1.9 Hz, 1H).

Intermediate 7-bromo-*N*-[4-(morpholin-4-yl)phenyl]quinazolin-2-amine (**M5**)

Intermediate **M4** (3.5 g, 14.50 mmol, 1.0 equiv), 4-morpholinylaniline (3.1 g, 17.40 mmol, 1.2 equiv), and anhydrous K_2_CO_3_ (4.0 g, 29.00 mmol, 2.0 equiv) were dissolved in anhydrous DMF (35 mL). The reaction mixture was stirred at 120 °C for 8 h under an argon atmosphere. Complete consumption of the starting material was confirmed by TLC analysis (DCM: MeOH = 30:1, *v*/*v*). After the mixture was allowed to cool to room temperature, the reaction mixture was slowly poured into distilled water (350 mL), upon which a yellow solid precipitated. The resulting suspension was filtered under vacuum, and the filter cake was collected. Methanol (20 mL) was added to the crude filter cake, and the mixture was stirred at room temperature for 15 min. The solid was collected by vacuum filtration, washed with methanol (2 mL × 2), and dried at 60 °C to afford **M5** as a yellow powdery solid (5.0 g, 89.8% yield). m.p. 209.8–210.5 °C. ^1^H NMR (600 MHz, DMSO-*d*_6_) δ 9.80 (s, 1H), 9.25 (s, 1H), 7.84–7.80 (m, 2H), 7.79 (d, *J* = 4.3 Hz, 2H), 7.46 (dd, *J* = 8.5, 1.9 Hz, 1H), 6.94 (d, *J* = 9.1 Hz, 2H), 3.76–3.74 (m, 4H), 3.07–3.05 (m, 4H). HRMS (ESI, *m*/*z*) calcd for C_18_H_17_BrN_4_NaO [M + Na]^+^, 407.0478; found 407.0506.

Intermediate (2-aminophenyl)dimethylphosphine oxide (**M6a**)

Commercially available 2-iodoaniline (120.3 mg, 0.60 mmol, 1.0 equiv), dimethylphosphine oxide (85.0 mg, 1.20 mmol, 2.0 equiv), and potassium phosphate (232.4 mg, 1.20 mmol, 2.0 equiv) were dissolved in anhydrous 1,4-dioxane (5.0 mL). Xantphos (64.4 mg, 0.11 mmol, 0.2 equiv) and Pd(OAc)_2_ (13.2 mg, 0.06 mmol, 0.1 equiv) were added, and the reaction mixture was stirred at 95 °C for 8 h under an argon atmosphere. Complete conversion of the starting material was confirmed by TLC analysis (DCM:MeOH = 30:1, *v*/*v*). The reaction mixture was filtered through a pad of Celite to remove insoluble materials, and the filtrate was collected and concentrated under reduced pressure to remove the solvent. The crude product was purified by preparative thin-layer chromatography (DCM: MeOH = 25:1, *v*/*v*); the product band was collected and concentrated under reduced pressure to remove the solvent. A mixed solvent of PE: EA (1:1, *v*/*v*, 4.5 mL) was added, and the mixture was stirred at room temperature for 15 min. The solid was collected by vacuum filtration, washed with the same mixed solvent (2 mL × 2), and dried at 60 °C to afford **M6a** as a brown powdery solid (47.2 mg, 50.5% yield). m.p. 168.3–169.1 °C.

Intermediate (3-aminophenyl) dimethylphosphine oxide (**M6b**).

Intermediate **M6b** was synthesized under the same conditions as those used for **M6a**. The compound was obtained as a brown powdery solid (33.3 mg, 34.7% yield).

Synthesis of Intermediate 4-Methylbenzenesulfonamide **(M7a**)

4-Methylbenzenesulfonyl chloride (100.0 mg, 0.52 mmol) was dissolved in dichloromethane (2 mL), and 25% aqueous ammonia (2.5 mL, 33.45 mmol) was added. The mixture was stirred vigorously at room temperature for 4 h. The reaction was monitored by TLC (PE:EA = 2:1, *v*/*v*) until complete consumption of the starting material was confirmed. The solvent was removed under reduced pressure, and the residue was dried at 60 °C to afford intermediate **M7a** as a white powdery solid (86.0 mg, 96.6% yield). m.p. 136.3–136.9 °C.

Intermediates **M7a**–**M7p** were prepared following the same general procedure.

#### 2.1.2. Synthesis of Compounds **H1**–**H28**

Dimethyl [2-({2-[(4-morpholin-4-ylphenyl)amino]quinazolin-7-yl}amino)phenyl]phosphine oxide (**H1**).

Intermediate **M5** (54.0 mg, 0.14 mmol, 1.0 equiv), **M6a** (47.1 mg, 0.28 mmol, 2.0 equiv), and Cs_2_CO_3_ (137.5 mg, 0.42 mmol, 3.0 equiv) were dissolved in anhydrous 1,4-dioxane (4 mL). Xantphos (16.3 mg, 0.028 mmol, 0.2 equiv) and Pd_2_(dba)_3_ (13.2 mg, 0.014 mmol, 0.1 equiv) were added, and the reaction mixture was stirred at 80 °C for 12 h under an argon atmosphere. The progress of the reaction was monitored by TLC (DCM:MeOH = 25:1, *v*/*v*), and complete conversion of the starting materials was confirmed. The reaction mixture was filtered through a pad of Celite to remove insoluble materials, and the filtrate was collected and concentrated under reduced pressure. The crude product was purified by column chromatography (DCM:MeOH = 150:1–100:1, *v*/*v*), and the solvent was removed under reduced pressure. Methanol (5 mL) was added to the resulting brownish-yellow solid, and the mixture was stirred at room temperature for 15 min. The solid was collected by vacuum filtration, washed with methanol (2 mL × 2), and dried at 60 °C to afford **H1**. Pale yellow powdery solid (20.3 mg, 30.3% yield). m.p. 186.3–187.1 °C. ^1^H NMR (600 MHz, DMSO-*d*_6_) δ 9.62 (s, 1H), 9.40 (s, 1H), 8.94 (s, 1H), 7.76 (d, *J* = 8.8 Hz, 2H), 7.71 (d, *J* = 8.5 Hz, 1H), 7.66 (dd, *J* = 13.6, 7.3 Hz, 1H), 7.58 (d, *J* = 3.9 Hz, 2H), 7.20–7.16 (m, 1H), 6.95 (d, *J* = 8.7 Hz, 2H), 6.91 (d, *J* = 9.1 Hz, 2H), 3.75–3.72 (m, 4H), 3.04–3.02 (m, 4H), 1.74 (d, *J* = 13.5 Hz, 6H). ^13^C NMR (151 MHz, DMSO-*d*_6_) δ 159.35, 156.96, 152.37, 148.16, 145.40, 144.22, 132.65, 132.17, 131.18 (d, *J* = 10.2 Hz), 128.77, 123.09 (d, *J* = 93.6 Hz), 121.79 (d, *J* = 11.7 Hz), 120.18 (d, *J* = 6.6 Hz), 119.32 (2C), 115.17, 115.04 (2C), 114.72, 104.87, 65.56 (2C), 48.70 (2C), 17.52, 17.04. HRMS (ESI, *m*/*z*) calcd for C_26_H_28_N_5_NaO_2_P [M + Na]^+^, 496.1873; found 496.1924. Retention time = 2.555 min, HPLC purity = 98.4%.

Target compounds **H2**–**H28** were prepared following the synthetic procedure used for **H1**.

The key characterization data for compounds **H2**–**H28**, including melting points, NMR, HRMS, and HPLC purity, have been reorganized and relocated to the Supporting Information.

### 2.2. In Vitro Biological Assays

#### 2.2.1. Plk4 Lanthascreen Eu Kinase Assay

Recombinant human PLK4 protein, LanthaScreen^TM^ Eu-labeled anti-GST antibody, and Kinase Tracer 236 were purchased from Thermo Fisher Scientific (Waltham, MA, USA). The positive control **LCR-263** was obtained from TargetMol (Boston, MA, USA). The LanthaScreen^TM^ Eu Kinase Binding Assay was performed according to the manufacturer’s protocol. Test compounds were dissolved in 100% DMSO to prepare 4 mM stock solutions. Prior to the assay, PLK4 protein, Kinase Tracer 236, and Eu-anti-GST antibody were diluted with 1× kinase buffer to working concentrations, yielding final concentrations of 1 nM, 1 nM, and 2 nM, respectively. For each assay, 4 μL of serially diluted compounds, 8 μL of the kinase/antibody mixture, and 4 μL of the tracer solution were sequentially added to the wells. The microplates were then incubated at 25 °C for 60 min, and data were acquired using an Infinite F500 microplate reader (Tecan, Männedorf, Switzerland). The emission ratio was calculated by dividing the acceptor emission intensity at 670 nm by the donor emission intensity at 612 nm. Data were analyzed using GraphPad Prism 10.1.2, and experiments were performed independently at least twice. For compounds with activity below the reliable lower quantification range of the assay, IC_50_ values are conservatively reported as <0.1 nM.

#### 2.2.2. Aurora A Kinase Assay

The activity of Aurora A was measured using the ADP-Glo Kinase Assay (Promega, Madison, WI, USA). 5 ng/μL Recombinant Human Aurora A, His-Tag (Sf9-derived, BPS Bioscience #40004A, San Diego, CA, USA) in 1× Kinase Assay Buffer (BPS Bioscience #79334, supplemented with optional 10 mM DTT) was incubated with serially diluted compound (in 10% DMSO/1× Kinase Buffer, 0.4% *v*/*v* final DMSO) for 30 min at room temperature. Next, 0.2 mg/mL Kemptide substrate (MedChemExpress, Monmouth Junction, NJ, USA) and 10 μM ATP (~Km) were added and incubated for 45 min at 30 °C in an assay volume of 25 μL. 25 μL of ADP-Glo Reagent was then added to stop the reaction and incubated for 45 min at room temperature. Next, 50 μL of Kinase Detection Reagent was added and incubated for 45 min at room temperature before detecting luminescence using a microplate reader capable of reading luminescence. Inhibition of Aurora A Kinase activity was normalized to DMSO (neutral control = 0% inhibition) and 1 µM Staurosporine (Millipore Sigma, St. Louis, MO, USA) or 1 µM Alisertib (positive control = 100% inhibition).

#### 2.2.3. Cell Antiproliferation Assay

Cell lines were cultured for at least three passages in their corresponding culture media: high-glucose DMEM was used for MCF-7 and HEK-293T cells, while IMR-32-specific medium was applied for the IMR-32 cell line. A total of 100 μL of cell suspension, adjusted to a density ranging from 1000 to 5000 cells/mL, was seeded into each well of a 96-well plate. After allowing the cells to adhere for 24 h in a humidified incubator supplemented with 5% CO_2_ at 37 °C, 100 μL of culture medium containing the test compound at the designated concentrations was added to each well, and the plates were further incubated for 5 d. Subsequently, the supernatant medium was carefully discarded, and 100 μL of fresh culture medium together with 10 μL of CCK-8 reagent was introduced into each well, followed by an additional incubation period ranging from 0.5 h to 5 h. The absorbance of each well was measured at a wavelength of 450 nm using an Infinite F500 microplate reader (Tecan, Switzerland). All experimental data were statistically analyzed using GraphPad Prism 10 software, and the half-maximal inhibitory concentration (IC_50_) values were calculated via non-linear regression fitting of the concentration-response curves.

#### 2.2.4. Colony Formation Assay

MCF-7 cells were seeded into 6-well plates at a density of 2000 cells per well and cultured in a humidified incubator at 37 °C under 5% CO_2_ for 1 day. After the medium was removed, cells were treated with compound **H24** at concentrations of 0.625, 1.25, 2.5, 5.0, and 10.0 μM, respectively, with medium containing 0.1% DMSO used as the vehicle control. The medium containing **H24** or DMSO was refreshed every three days. After 14 days of culture, cells were washed twice with phosphate-buffered saline (PBS) and fixed with 4% paraformaldehyde solution for 0.5 h. After the fixative was removed, cells were washed twice with PBS and stained with crystal violet solution (Beyotime Biotechnology, Shanghai, China) for 20 min. Following thorough rinsing with distilled water, colonies were imaged, and quantitative analysis was performed using ImageJ 1.54 software.

#### 2.2.5. Cell Apoptosis Assay

MCF-7 cells were initially seeded into 6-well plates at a density of 4 × 10^5^ cells per well and cultured in a humidified incubator at 37 °C under 5% CO_2_ for 1 day. After the culture medium was removed, cells were incubated with compound **H24** at various concentrations or DMSO for 2 days. Following treatment, the resulting cell pellets were collected and washed twice with pre-cooled PBS buffer. Subsequently, cells were stained according to the manufacturer’s protocol of the Annexin V-FITC/PI Apoptosis Detection Kit (Beyotime Biotechnology). Immediately after staining, samples were analyzed using a Guava easyCyte flow cytometer (Merck, Darmstadt, Germany) to quantify the proportion of apoptotic cells.

#### 2.2.6. Cell Cycle Analysis

MCF-7 cells were seeded into 6-well plates at a density of 4 × 10^5^ cells per well and cultured in a humidified incubator at 37 °C under 5% CO_2_ for 1 day. The supernatant was removed, and cells were incubated with compound **H24** at various concentrations or DMSO for 2 days. After treatment, cells were collected and washed twice with pre-cooled PBS. Subsequently, pre-cooled 70% ethanol was slowly added, and cells were fixed overnight at 4 °C. The following day, the fixed cells were collected by centrifugation, and the supernatant was discarded. Cells were washed twice with pre-cooled PBS and then stained with propidium iodide (PI) in the dark at 37 °C for 0.5 h. Immediately after staining, samples were analyzed using a Guava easyCyte flow cytometer (Merck).

#### 2.2.7. Molecular Docking

The PLK4 kinase crystal structure (PDB code: 4YUR) was downloaded from the RCSB Protein Data Bank (https://www.rcsb.org/, accessed on 15 March 2026). The structure was preprocessed using the Protein Preparation Wizard tool in Schrödinger Maestro 13.5, which included the addition of hydrogen atoms, removal of excess water molecules, and energy minimization. All hydrogen bonds were optimized using the OPLS_2005 force field. The optimized protein structure was used as the receptor, and the docking grid was generated using the Receptor Grid Generation module. The docking box was centered on the co-crystallized ligand with dimensions of 20 Å × 20 Å × 20 Å. The compound structures were optimized using the LigPrep module. Molecular docking was performed using the Ligand Docking module in Standard Precision (SP) mode, and the visualization and analysis of the docking results were carried out using PyMOL 3.0.3 software.

#### 2.2.8. Human Liver Microsomal Stability Assay

The incubation buffer consisted of 3 mM MgCl_2_-PB, with final concentrations of 0.5 mg/mL liver microsomes, 0.2 μM midazolam, 1 μM test compound, and 1 mM *β*-NADPH. Liver microsomes were pre-warmed at 37 °C for 5 min, and a 2 mM *β*-NADPH working solution was prepared in MgCl_2_-PB buffer. The cofactor-free incubation mixture was prepared and dispensed into designated tubes. Zero-minute samples were generated by spiking aliquots of the basal mixture with *β*-NADPH working solution. Reactions were initiated by the addition of *β*-NADPH working solution to all tubes, whereas an equivalent volume of MgCl_2_-PB buffer was added to the negative control group in place of *β*-NADPH. The test compound group was incubated at 37 °C for 5, 15, 30, and 60 min, while the positive control group was incubated for 5 and 15 min only. At each predetermined time point, 60 μL of the reaction mixture was withdrawn and quenched with 300 μL of precipitation solution containing an internal standard. Neat samples were prepared in parallel. Following centrifugation, 150 μL of the supernatant was collected, diluted with 150 μL of deionized water, and subjected to LC-MS/MS quantification.

## 3. Results and Discussion

### 3.1. Chemical Synthesis

**Scheme 1:**
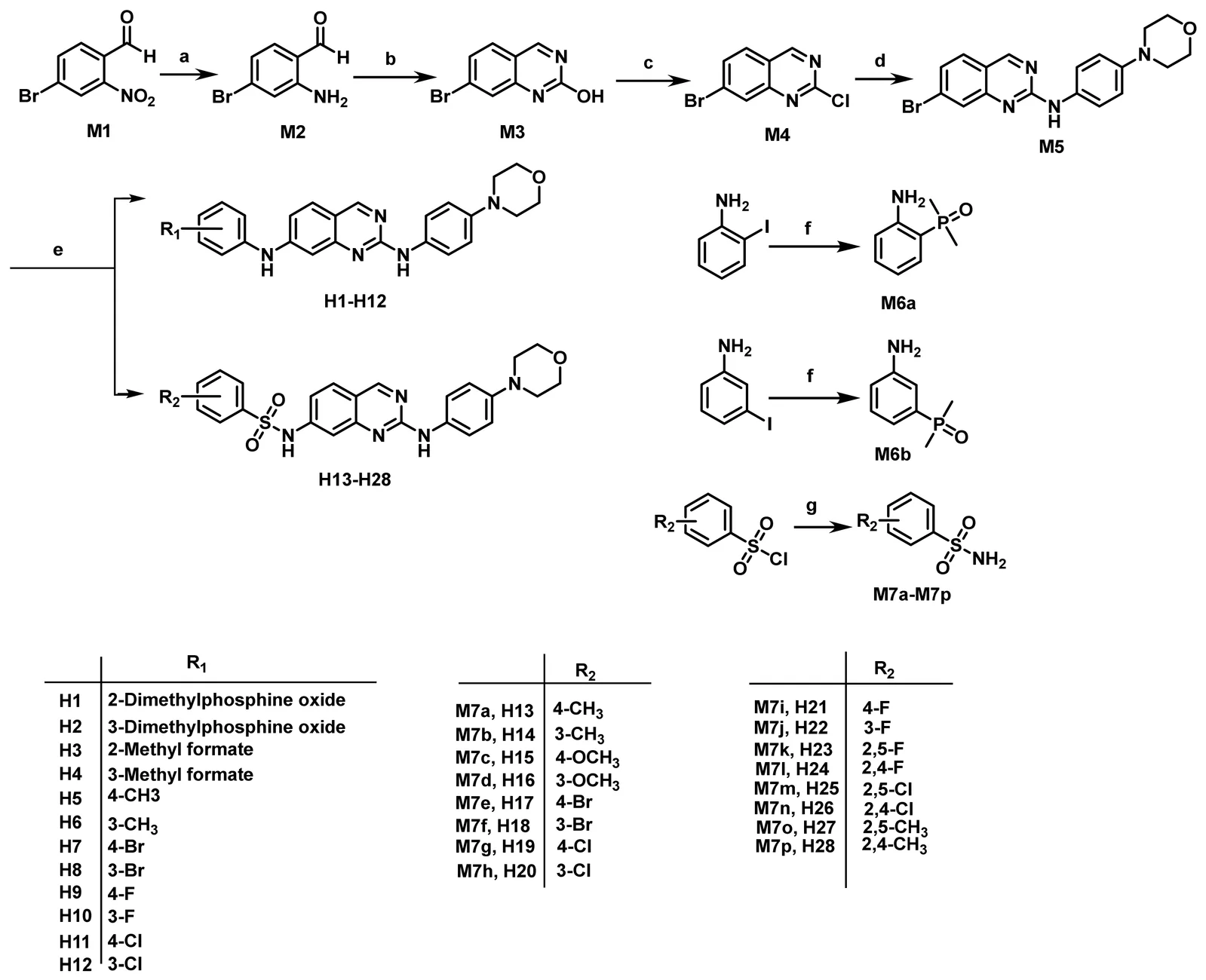
Synthesis of compounds **M6a**, **M6b**, **M7a**–**p**, and **H1**–**H28**. Reagents and conditions: (a) Fe, HCl, V(EtOH):V(H_2_O) = 4:1, Ar, r.t.; (b) Urea, 180 °C, reflux; (c) POCl_3_, 110 °C, reflux; (d) 4-Morpholinoaniline, K_2_CO_3_, DMF, Ar, 120 °C; (e) Cs_2_CO_3_, Pd_2_(dba)_3_, Xantphos, dry 1,4-dioxane, Ar, 80 °C; (f) Dimethylphosphine oxide, K_3_PO_4_, Pd(OAc)_2_, Xantphos, dry 1,4-dioxane, Ar, 95 °C. (g) Ammonia (25%), DCM, r.t.

The synthetic routes of target compounds **H1**–**H28**, intermediates **M6a**, **M6b**, and intermediates **M7a–p** are outlined in Scheme 1.

The synthesis commenced with commercially available 4-bromo-2-nitrobenzaldehyde (**M1**), which was reduced to 4-bromo-2-aminobenzaldehyde (**M2**) via an Fe/HCl reduction system. Intermediate **M2** was then subjected to a cyclocondensation reaction with urea to afford the intermediate 7-bromoquinazolin-2-ol (**M3**). Chlorination of **M3** was carried out with POCl_3_ to yield 7-bromo-2-chloroquinazoline (**M4**). A nucleophilic aromatic substitution reaction between **M4** and 4-morpholinoaniline was then performed to furnish the key intermediate 7-bromo-*N*-[4-(morpholin-4-yl)phenyl]quinazolin-2-amine (**M5**). Finally, compound **M5** was coupled with various intermediates via Buchwald–Hartwig cross-coupling reactions to afford the target compounds **H1**–**H28**. Intermediates **M6a** and **M6b** were prepared from 2-iodoaniline and 3-iodoaniline, respectively, through Pd-catalyzed Hirao C–P coupling reactions. A series of benzenesulfonamide intermediates **M7a–p** were obtained by ammonolysis of commercially available benzenesulfonyl chloride derivatives with aqueous ammonia.

### 3.2. Design of Target Compounds and In Vitro Plk4 Kinase Inhibitory Activity

**Figure 3:**
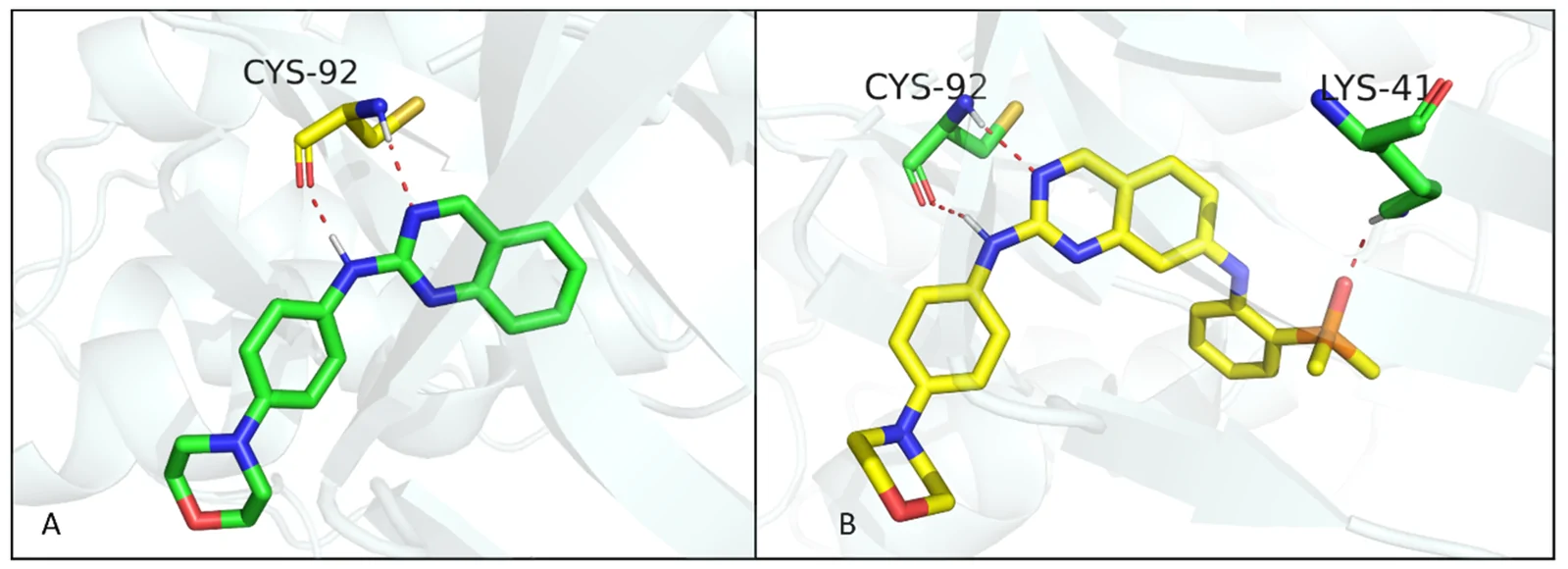
(**A**) Predicted binding mode of compound **ZSL-M001** (depicted in green model) in the ATP-binding site of PLK4. (**B**) Predicted binding mode of compound **H1** (depicted in yellow model) in the ATP-binding site of PLK4 (PDB code: 4YUR).

The docking model predicted two potential hydrogen-bond interactions for **ZSL-M001** bound to the crystal structure of PLK4 (PDB code: 4YUR) within the ATP-binding pocket (Figure 3A): one is formed between the quinazoline nitrogen and the backbone N-H of hinge residue Cys-92, and the other is formed between the aniline substituent on the parent scaffold and the oxygen atom of Cys-92. This dual hydrogen-bond network anchors **ZSL-M001** in the kinase domain. However, the docking model also suggested that the hydrophobic region on the right side was not sufficiently occupied within the hydrophobic pocket [36], indicating room for further structural modification. Meanwhile, the 4-morpholinylaniline moiety extending toward the solvent-exposed region on the left side was retained based on our previous structure-activity relationship studies. Therefore, in the first round of structural optimization, a side-chain extension strategy was adopted to target the unexploited hydrophobic pocket. Notably, these molecular docking-based observations applied to guide compound design should be interpreted as hypotheses rather than experimentally verified binding mechanisms.

On the one hand, preferred hydrogen-bond acceptors such as dimethylphosphine oxide and the methoxycarbonyl group were introduced to potentially enable hydrogen-bond interactions with Lys-41, and based on this rationale, compounds **H1**–**H4** were designed and synthesized with improved PLK4 inhibitory activity. However, the potencies remained inferior to that of the positive control **LCR-263**, which fell short of our expectations. On the other hand, hydrophobic substituents, including methyl, fluoro, bromo, and chloro substituents, were introduced at the 3- or 4-position of the phenyl ring to further occupy the hydrophobic cavity, affording compounds **H5**–**H12** (Table 1), but the PLK4 inhibitory activity did not improve. Based on our previous structure-activity relationship studies and the molecular docking model of compound **H1** (Figure 3B), we speculated that potential hydrogen-bond formation with Lys-41 and the introduction of hydrophobic groups of appropriate size may contribute to improved activity. Accordingly, the aniline fragment was replaced with a benzenesulfonamide moiety capable of adopting a folded conformation in the docking model. This design was intended to maintain possible hydrogen-bond interactions while positioning the hydrophobic moiety into the cavity, thereby improving pocket occupancy and inhibitory activity.

To further occupy the hydrophobic cavity, methoxy, methyl, bromo, chloro, and fluoro substituents were individually introduced at the 4- or 3-position of the benzene ring on the basis of the sulfonamide scaffold, yielding compounds **H13**–**H22** (Table 2). Preliminary activity evaluation demonstrated that all compounds in this series exhibited superior potency compared to the aniline-based compounds obtained from the first round of optimization. When methyl, chloro, fluoro, or bromo substituents were introduced at the 4-position, or chloro or fluoro substituents were introduced at the 3-position, the resulting compounds displayed PLK4 inhibitory activity superior to both the lead compound **ZSL-M001** and the positive control **LCR-263**. Notably, the 4-bromo-substituted compound exhibited excellent potency (IC_50_ < 0.1 nM), whereas the 3-bromo-substituted analog showed a marked decrease in activity (IC_50_ = 6.2 nM). This activity difference may be related to steric incompatibility predicted by the docking model, which could compromise ligand-protein interactions. The activity trend observed for methoxy-substituted compounds was similar to that of the bromo series; however, both the 4-methoxy and 3-methoxy derivatives exhibited reduced activity (IC_50_ = 9.2 nM and 73.0 nM, respectively). In particular, the pronounced activity reduction observed for the 3-methoxy derivative may reflect the combined influence of polarity and steric fit within the pocket. These mechanistic explanations remain docking-guided hypotheses and require further structural or biophysical validation.

Based on the potent inhibitory activities exhibited by the monosubstituted compounds, further exploration of disubstituted analogs was undertaken, primarily focusing on the introduction of a second substituent at the 2-position to further fill the protein cavity, while retaining the 3- or 4-position fluoro, chloro, or methyl substituents. Given that the bromine substituents are less frequently employed in drug design, it was not extensively explored in the present optimization. Ultimately, compounds **H23**–**H28** were obtained. The results obtained from in vitro kinase activity assays (Table 3) indicated that the kinase inhibitory activities of the disubstituted compounds were largely maintained.

### 3.3. In Vitro Cell Antiproliferative Activity Assay

As reported by Meitinger et al. [37,38], PLK4 inhibition induces centrosome loss and exerts synthetic lethality in *TRIM37*-amplified cancer cells. Based on this rationale, we selected compounds with potent PLK4 kinase inhibitory activity (<10.0 nM) for in vitro anti-proliferative evaluation in the *TRIM37*-amplified breast cancer cell line MCF-7, using **LCR-263** as a positive control (Table 4).

A notable discrepancy was observed between the PLK4 kinase inhibitory potency and the in vitro antiproliferative activity of the compounds. In this context, the enzymatic inhibition data obtained from the PLK4 kinase assay are interpreted as a quantitative measure of direct target engagement under standardized cell-free reaction conditions. In contrast, the cellular antiproliferative activity is considered to be modulated by a complex interplay of multiple interfering factors, including cell membrane permeability (as reflected by the cLogP values predicted using ChemDraw 20.0 (Table S2), which were in the range of 4–5 for our compounds, whereas values of 1–3 aregenerally considered optimal for cellular penetration. It should be noted that these ChemDraw-generated cLogP values only serve as a rough qualitative reference for compound hydrophobicity and are not intended for precise quantitative analysis; they are also not intended for use in predicting intracellular compound accumulation, plasma protein binding, drug efflux transporter activity, intracellular ATP competition, dependency on intact oncogenic signaling pathways, the intrinsic genetic background of different cell lines, and potential off-target inhibition of other mitotic kinases. Consequently, the cellular antiproliferative activity cannot be directly equated with the enzymatic inhibitory potency on a numerical basis, and further in-depth mechanistic investigations into its cellular pharmacodynamics are warranted.

To further investigate whether the compounds exhibit *TRIM37*-amplification-dependent cellular selectivity, the antiproliferative activities of **H22**–**H25**, which were the most potent analogs identified in the MCF-7 in vitro assay, were additionally evaluated against three cell lines: IMR-32 cells, which have high *TRIM37* amplification, A549 cells, which are not *TRIM37*-highly amplified, and HEK-293T control cells (Table 5).

The additional cell-line evaluation did not show a clear selective activity pattern for *TRIM37*-high cells over non-*TRIM37*-high or normal control cells. Taken together, among the tested compounds, compound **H24**, which exhibited superior overall properties, was selected as the lead candidate for subsequent testing and evaluation.

Given that compound **H24** did not exhibit obvious selectivity across the cell lines tested, we proceeded to further evaluate it in a preliminary Aurora A counter-screen. Aurora A kinase, which shares a high degree of homology with PLK4, was selected for the activity assay. The results showed that **H24** inhibited Aurora A with an IC_50_ value of 151.2 nM (Figure S2). Although **H24** remains more potent against PLK4 in the biochemical assay, this measurable off-target activity nonetheless indicates that the kinase selectivity of the compound has not yet been optimized to a satisfactory level. This observation may, to some extent, explain why no clear *TRIM37*-expression-dependent differential antiproliferative activity was observed across the multiple cell lines screened.

### 3.4. Effects of Compound ***H24*** on Colony Formation

**Figure 4:**
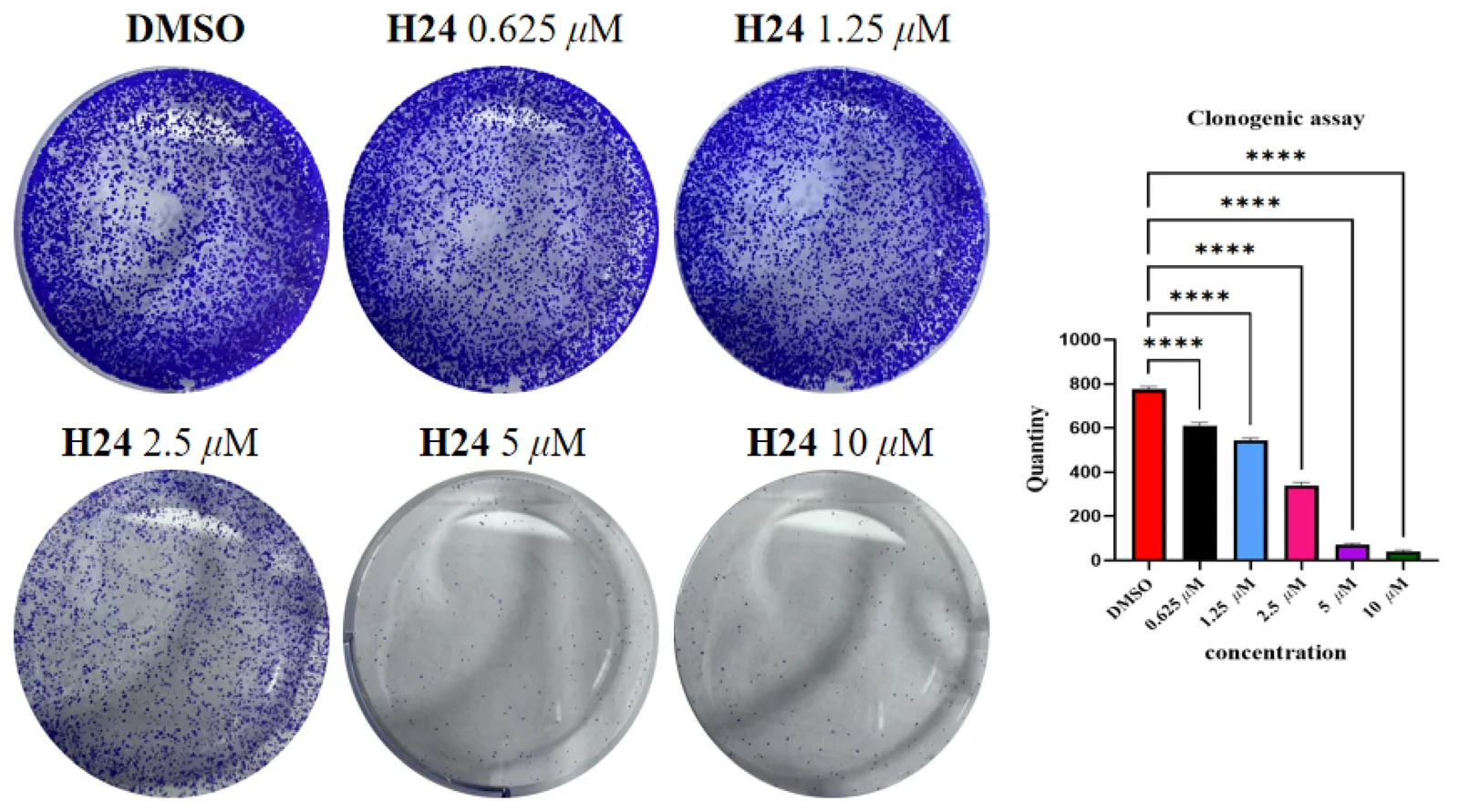
Colony formation in MCF-7 cells following treatment with DMSO or compound **H24**. The significance of the differences between the vehicle group and the administration groups was determined by a one-way ANOVA test. Columns, mean; bars, standard deviation. **** *p* < 0.0001.

The results of the colony formation assay (Figure 4) indicated that compound **H24** inhibited the colony formation of breast cancer MCF-7 cells in a concentration-dependent manner, with a marked inhibitory effect observed at a concentration of 5.0 μM. This result supports the cellular antiproliferative activity of **H24** in MCF-7 cells.

### 3.5. Flow Cytometry Experiment

**Figure 5:**
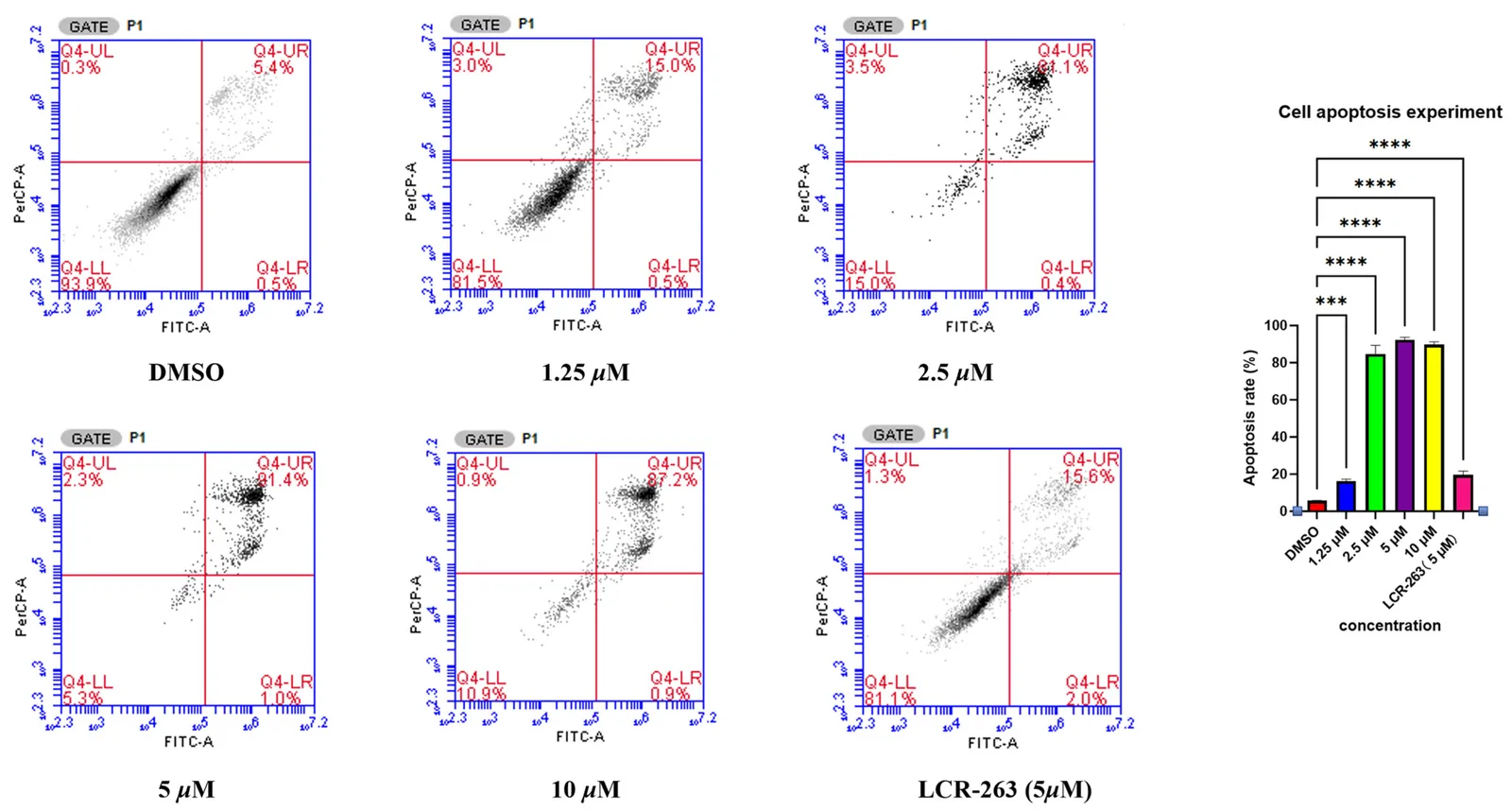
Effects of compound **H24** on apoptosis in MCF-7 cells. The significance of the differences between the vehicle group and the administration groups was determined by a one-way ANOVA test. Columns, mean; bars, standard deviation. *** *p* < 0.001; **** *p* < 0.0001.

Flow cytometry was used to evaluate the ability of compound **H24** to induce apoptosis in MCF-7 cells. The results (Figure 5) showed that **H24** induced apoptosis in MCF-7 cells in a concentration-dependent manner. The apoptosis rates were 15.5%, 81.5%, and 92.4% at concentrations of 1.25 μM, 2.5 μM, and 5.0 μM, respectively. Notably, the apoptosis rate induced by **H24** at 5.0 μM was higher than that of the positive control **LCR-263** (17.6% at 5.0 μM).

**Figure 6:**
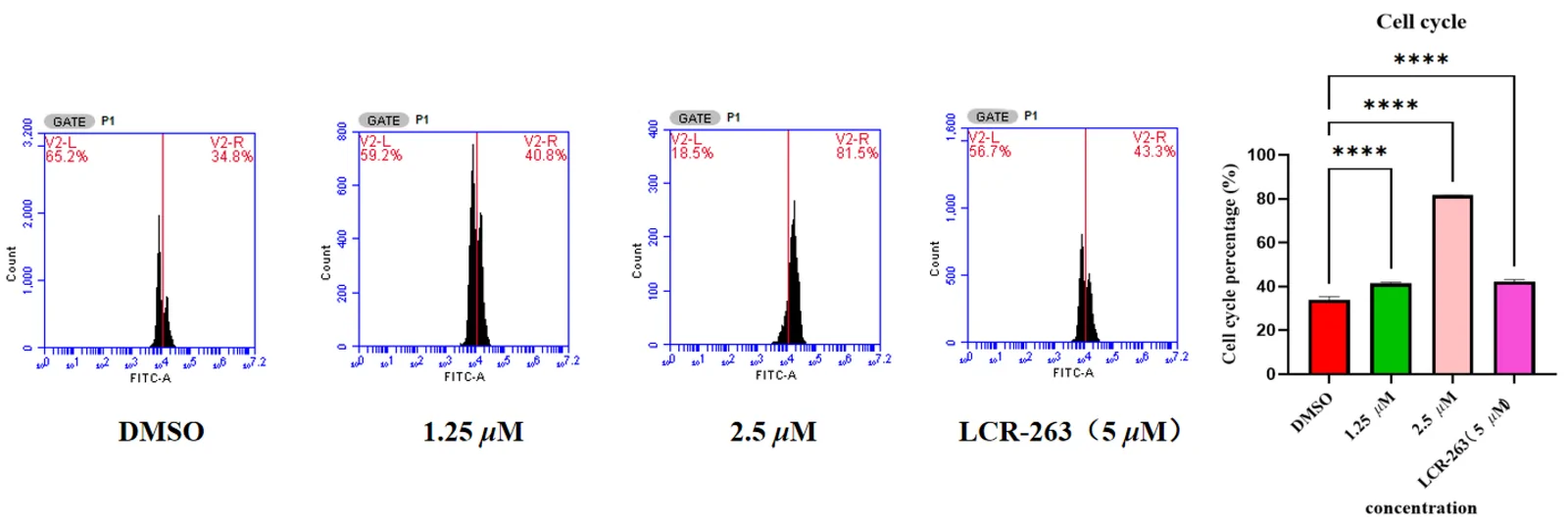
Effects of compound **H24** on the cell-cycle distribution of MCF-7 cells. The significance of the differences between the vehicle group and the administration groups was determined by a one-way ANOVA test. Columns, mean; bars, standard deviation. **** *p* < 0.0001.

The effect of compound **H24** on the cell cycle of MCF-7 cells was analyzed by flow cytometry. The experimental results (Figure 6) revealed that with increasing concentrations of **H24**, the proportion of MCF-7 cells in the S/G2 phase increased, while the proportion of cells in the G1 phase decreased. The S/G2 phase arrest ratios at concentrations of 1.25 μM and 2.5 μM were 40.8% and 81.5%, respectively. These results indicate that **H24** concentration-dependently arrested MCF-7 cells in the S/G2 phase, and its effect was stronger than that of the positive control **LCR-263** (5.0 μM, 43.3%).

### 3.6. Human Liver Microsome Stability Assay

To evaluate the rate of hepatic metabolic degradation of **H24**, a human liver microsome (HLM) incubation model was established. The results (Table 6) demonstrated that compound **H24** exhibited a half-life (t_1/2_) of 61.3 min and an intrinsic clearance (CL_int_) of 0.0226 mL/min/mg. These findings indicate that **H24** possesses favorable hepatic metabolic stability with a moderate hepatic clearance rate in this in vitro system. Nevertheless, microsomal stability alone cannot predict in vivo pharmacokinetics, systemic exposure, tissue distribution, or safety. Therefore, further in vivo pharmacokinetic studies are required to further evaluate **H24**.

## 4. Conclusions

Based on the PLK4 kinase inhibitor **ZSL-M001** (IC_50_ = 3.0 μM) identified in our previous work, 28 novel PLK4 inhibitors featuring a quinazoline core scaffold were designed and synthesized through side-chain extension strategies, followed by biological evaluation. Among these compounds, **H24** (PLK4 IC_50_ < 0.1 nM) exhibited potent in vitro antiproliferative activity against the *TRIM37*-amplified breast cancer cell line MCF-7 (IC_50_ = 2.37 ± 0.26 μM) and IMR-32 (IC_50_ = 1.28 ± 0.04 μM), which was superior to that of the positive control **LCR-263** (MCF-7 IC_50_ > 4 μM; IMR-32 IC_50_ = 1.82 ± 0.06 μM). **H24** also inhibited colony formation of MCF-7 cells in a concentration-dependent manner, induced S/G2-phase cell-cycle arrest, triggered apoptosis, and showed favorable human liver microsomal stability with a half-life of 61.3 min. However, additional testing in *TRIM37* non-highly amplified A549 cells and HEK-293T cells did not establish a clear *TRIM37*-dependent cellular selectivity pattern, and the Aurora A counter-screen (IC_50_ = 151.2 nM) indicated that kinase selectivity remains to be improved. Meanwhile, molecular docking suggested possible interactions with Cys-92 and Lys-41 and improved occupancy of the hydrophobic pocket; however, these binding hypotheses require structural or biophysical validation. Therefore, **H24** should be regarded as a potent biochemical PLK4 inhibitor and a useful quinazoline-based lead scaffold for further optimization, rather than as a fully selective cellular or in vivo candidate. Future work will focus on improving kinase selectivity, confirming cellular PLK4 target engagement, and evaluating broader drug-like properties and in vivo pharmacokinetic behavior.

## Figures and Tables

**Table 1:** In vitro PLK4 inhibition activities (IC_50_) of compounds **H1**–**H12**.

Entry	R	PLK4 IC_50_ (μM) ^a^	Entry	R	PLK4 IC_50_ (μM) ^a^
**H1**	2-P(CH_3_)_2_O	0.25	**H8**	3-Br	0.70
**H2**	3-P(CH_3_)_2_O	0.74	**H9**	4-F	2.66
**H3**	2-COOCH_3_	0.72	**H10**	3-F	4.05
**H4**	3-COOCH_3_	0.67	**H11**	4-Cl	1.17
**H5**	4-CH_3_	>50	**H12**	3-Cl	1.35
**H6**	3-CH_3_	>50	**ZSL-M001**	-	3.0
**H7**	4-Br	0.75	**LCR-263** ^b^	-	0.003

^a^ Values exceeding the reliable upper quantification limit of the assay are conservatively reported as >50 μM. ^b^ **LCR-263** is a positive control.

**Table 2:** In vitro PLK4 inhibition activity (IC_50_) of compounds **H13**–**H22**.

Entry	R	PLK4 IC_50_ (nM) ^a^	Entry	R	PLK4 IC_50_ (nM) ^a^
**H13**	4-CH_3_	0.3	**H19**	4-Cl	<0.1
**H14**	3-CH_3_	<0.1	**H20**	3-Cl	<0.1
**H15**	4-OCH_3_	9.2	**H21**	4-F	1.3
**H16**	3-OCH_3_	73.0	**H22**	3-F	0.3
**H17**	4-Br	<0.1	**ZSL-M001**	-	3000
**H18**	3-Br	6.2	**LCR-263** ^b^	-	3.0

^a^ Values below the reliable lower quantification range of the assay are conservatively reported as <0.1 nM. ^b^ **LCR-263** is a positive control.

**Table 3:** In vitro PLK4 inhibition activity (IC_50_) of compounds **H23**–**H28**.

Entry	R	PLK4 IC_50_ (nM) ^a^	Entry	R	PLK4 IC_50_ (nM) ^a^
**H23**	2,5-F	1.0	**H27**	2,5-CH_3_	<0.1
**H24**	2,4-F	<0.1	**H28**	2,4-CH_3_	<0.1
**H25**	2,5-Cl	0.1	**ZSL-M001**	-	3000
**H26**	2,4-Cl	<0.1	**LCR-263** ^b^	-	3.0

^a^ Values below the reliable lower quantification range of the assay are conservatively reported as <0.1 nM. ^b^ **LCR-263** is a positive control.

**Table 4:** Antiproliferative activity of compounds against MCF-7 cells.

Entry	MCF-7 IC_50_ (μM) ^a^	Entry	MCF-7 IC_50_ (μM) ^a^	Entry	MCF-7 IC_50_ (μM) ^a^
**H13**	>4	**H19**	>4	**H25**	2.98 ± 0.35
**H14**	>4	**H20**	>4	**H26**	>4
**H15**	>4	**H21**	>4	**H27**	>4
**H16**	>4	**H22**	3.35 ± 0.62	**H28**	>4
**H17**	>4	**H23**	2.97 ± 0.26	**LCR-263** ^b^	>4
**H18**	>4	**H24**	2.37 ± 0.26	**-**	-

^a^ IC_50_ values represent mean ± standard error of the mean (SEM) with *n* ≥ 2 independent biological replicates, and the corresponding 95% confidence intervals are listed in Supporting Information Table S1. Values labeled > 4 μM indicate less than 50% cell growth inhibition at the maximum tested concentration of 4 μM. ^b^ **LCR-263** is a positive control.

**Table 5:** Antiproliferative activity of compounds against IMR-32, A549, and HEK-293T cells.

Entry	IMR-32 IC_50_ (μM) ^a^	A549 IC_50_ (μM) ^a^	HEK-293T IC_50_ (μM) ^a^
**H22**	1.17 ± 0.10	1.60 ± 0.14	1.32 ± 0.24
**H23**	1.30 ± 0.18	1.19 ± 0.07	1.37 ± 0.05
**H24**	1.28 ± 0.04	1.47 ± 0.04	1.48 ± 0.30
**H25**	2.05 ± 0.08	1.55 ± 0.03	1.07 ± 0.10
**LCR-263** ^b^	1.82 ± 0.06	1.40 ± 0.38	0.58 ± 0.10

^a^ IC_50_ values represent mean ± standard error of the mean (SEM) with *n* ≥ 2 independent biological replicates, and the corresponding 95% confidence intervals are listed in Supporting Information Table S1. ^b^ **LCR-263** is a positive control.

**Table 6:** Liver Microsomal Stability of Compound **H24**.

Compound	Species	R^2^	T_1/2_ (min)	In Vitro CL_int_ (mL/min/mg)
**H24**	Human	0.9870	61.3	0.0226

R^2^: Linear correlation coefficient; CL_int_: intrinsic clearance.

## Data Availability

The data presented in this study are available upon reasonable request from the corresponding author.

## Supplementary Materials

The following supporting information can be downloaded at: https://www.mdpi.com/article/10.3390/biomedicines14081811/s1. Section S1. Kinase inhibitory activity of Compound **H24**. Figure S1. Dose–response curve of **H24** for PLK4 kinase inhibition. Figure S2. Dose–response curve of **H24** for Aurora A kinase inhibitory activity. Section S2. *In vitro* anti-proliferative activity of Compound **H24**. Figure S3. Dose–response curve of **H24** for in vitro antiproliferative activity in MCF-7 cells. Figure S4. Dose–response curve of **H24** for in vitro antiproliferative activity in IMR-32 cellsa. Figure S5. Dose–response curve of **H24** for in vitro antiproliferative activity in A549 cellsa. Figure S6. Dose–response curve of **H24** for in vitro antiproliferative activity in HEK-293T cellsa. Section S3. Mean IC_50_ (95% CI) of compounds. Table S1. IC50 values and 95% confidence intervals from independent experiments. Section S4. cLogP data of the compound. Table S2. Predicted cLogP data for compounds **H13**–**H28**. Section S5. Key characterization data for compounds **H1–H28**. Section S6. Spectroscopic data of key compounds.
