# Supplementary material for: Design, Synthesis, and Biological Activity Evaluation of Quinazoline-Based PLK4 Inhibitors
Source: Biomedicines. 2026 Aug 12;14(8):1811. doi: 10.3390/biomedicines14081811 (PMC13509253; doi:10.3390/biomedicines14081811)
Supplement: Supplementary file 1 [file biomedicines-14-01811-s001.zip › biomedicines-4395147-supplementary.pdf]

Design, Synthesis, and Biological Activity Evaluation of Quinazoline-Based PLK4 Inhibitors

Wenqiang Sun †, Zehui Qi †, Ningyuan Hu †, Nian Liu †, Shuyi Mu, Haoyu Zhang, Zixuan Gao, Zirui Luo, Yin Sun, Dongmei Zhao * and Maosheng Cheng

Key Laboratory of Structure-Based Drug Design and Discovery, Ministry of Education, School of Pharmaceutical Engineering, Shenyang Pharmaceutical University, 103 Wenhua Road, Shenhe District, Shenyang 110016, China; yinsun@stanford.edu (Y.S.)

* Correspondence: medchemzhao@163.com

† These authors contributed equally to this work.

Contents

Design, Synthesis, and Biological Activity Evaluation of Quinazoline-based PLK4 Inhibitors 1

S1. Kinase inhibitory activity of Compound **H24**.. 3

S2. *In vitro* anti-proliferative activity of Compound **H24**.. 4

S3. Mean IC_{50} (95% CI) of compounds 6

S4. cLogP data of the compound. 7

S5. Key characterization data for compounds **H1–H28**.. 8

S6. Spectroscopic data of key compounds 18

S1. Kinase inhibitory activity of Compound **H24**.

Figure S1. Dose–response curve of **H24** for PLK4 kinase inhibition.

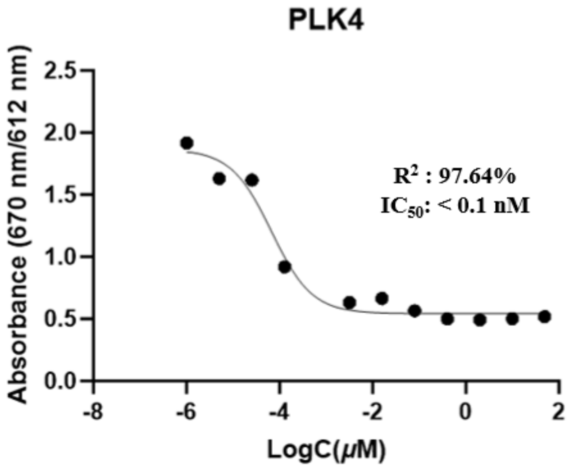

Figure S2. Dose–response curve of **H24** for Aurora A kinase inhibitory activity.

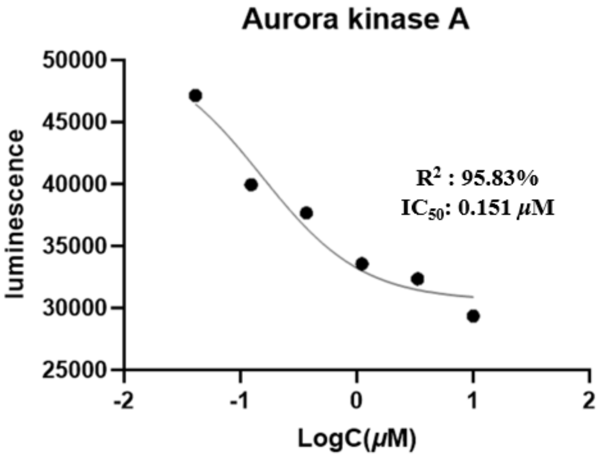

S2. *In vitro* anti-proliferative activity of Compound **H24**.

Figure S3. Dose–response curve of **H24** for in vitro antiproliferative activity in MCF-7 cells.

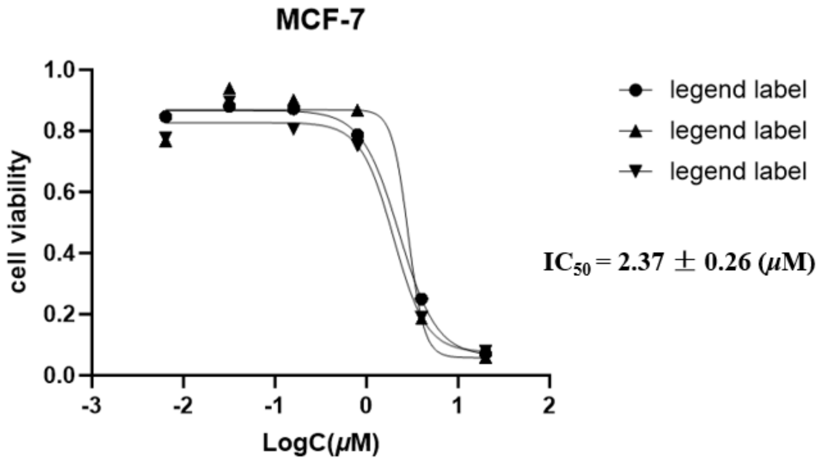

[a]IC_{50} values represent mean ± standard error of the mean (SEM) with n ≥ 2 independent biological replicates, and the corresponding 95% confidence intervals are listed in Supporting Information **Table S1**.

Figure S4. Dose–response curve of **H24** for in vitro antiproliferative activity in IMR-32 cells[a].

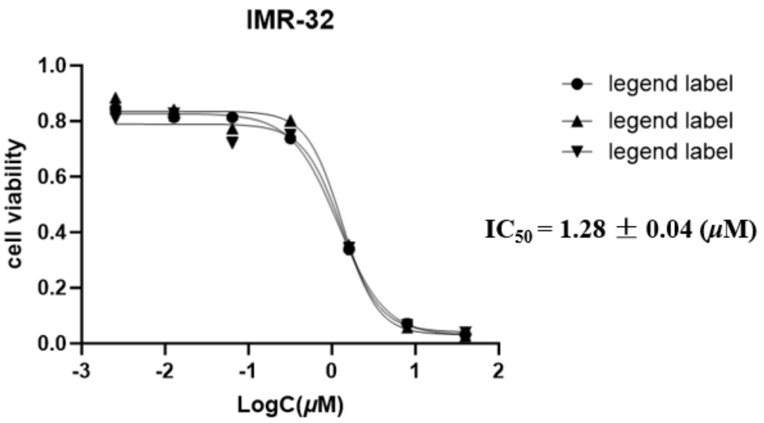

[a]IC_{50} values represent mean ± standard error of the mean (SEM) with n ≥ 2 independent biological replicates, and the corresponding 95% confidence intervals are listed in Supporting Information **Table S1**.

Figure S5. Dose–response curve of **H24** for in vitro antiproliferative activity in A549 cells[a].

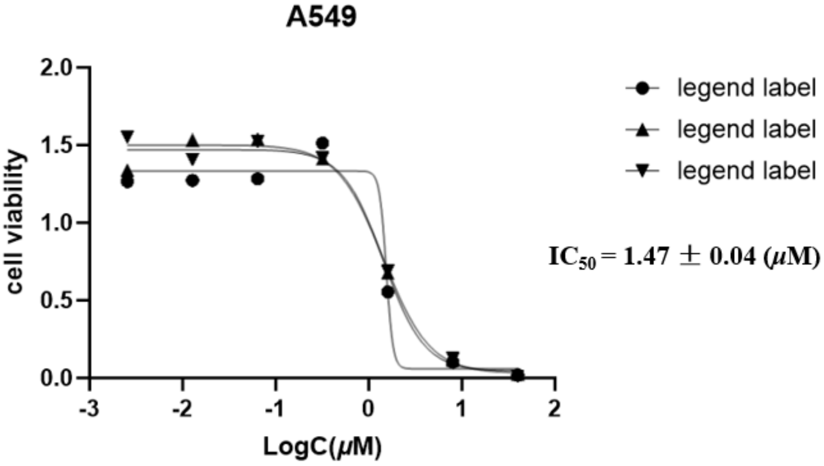

[a]IC_{50} values represent mean ± standard error of the mean (SEM) with $n \geq 2$ independent biological replicates, and the corresponding 95% confidence intervals are listed in Supporting Information **Table S1**.

Figure S6. Dose–response curve of **H24** for in vitro antiproliferative activity in HEK-293T cells[a].

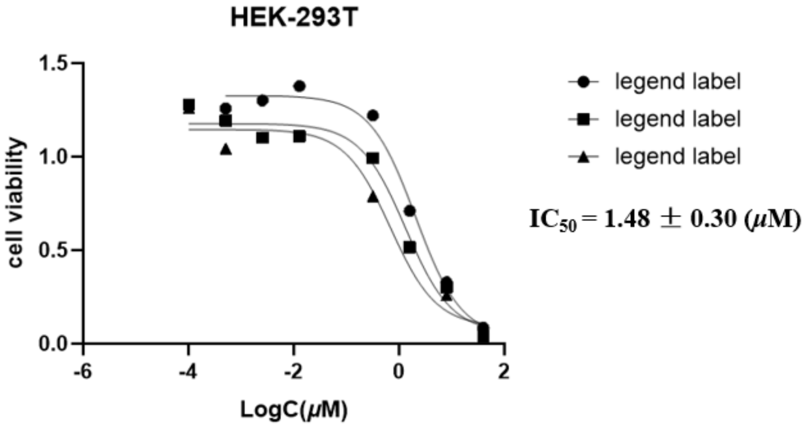

[a]IC_{50} values represent mean ± standard error of the mean (SEM) with $n \geq 2$ independent biological replicates, and the corresponding 95% confidence intervals are listed in Supporting Information **Table S1**.

S3. Mean IC_{50} (95% CI) of compounds

Table S1. IC_{50} values and 95% confidence intervals from independent experiments.

Entry	**MCF-7 (*μ*M)[a]**		**IMR-32 (*μ*M)[a]**		**A549 (*μ*M)[a]**		**HEK-293T (*μ*M)[a]**	
	IC_{50}	95% CI of IC_{50}	IC_{50}	95% CI of IC_{50}	IC_{50}	95% CI of IC_{50}	IC_{50}	95% CI of IC_{50}
**H22**	3.35 ± 0.62	0.70, 6.01	1.17 ± 0.10	0.73, 1.62	1.60 ± 0.14	1.02, 2.18	1.32 ± 0.24	0.30, 2.34
**H23**	2.97 ± 0.26	1.87, 4.07	1.30 ± 0.18	0.51, 2.10	1.19 ± 0.07	0.88, 1.49	1.37 ± 0.05	0.77, 1.97
**H24**	2.37 ± 0.26	1.26, 3.47	1.28 ± 0.04	1.10, 1.45	1.47 ± 0.04	1.32, 1.62	1.48 ± 0.30	0.18, 2.77
**H25**	2.98 ± 0.35	1.47, 4.49	2.05 ± 0.08	1.69, 2.42	1.55 ± 0.03	1.41, 1.68	1.07 ± 0.10	0.65, 1.49

[a]IC_{50} values represent mean ± standard error of the mean (SEM) with $n \geq 2$ independent biological replicates.

S4. cLogP data of the compound.

Table S2. Predicted cLogP data for compounds **H13–H28**.

Entry	PLK4 IC_{50} (nM)	MCF-7 IC_{50} (μM)[a]	cLogP[b]
**H13**	0.3	> 4	4.7
**H14**	< 0.1	> 4	4.7
**H15**	9.2	> 4	4.4
**H16**	73.0	> 4	4.4
**H17**	< 0.1	> 4	5.1
**H18**	6.2	> 4	5.1
**H19**	< 0.1	> 4	5.0
**H20**	< 0.1	> 4	5.0
**H21**	1.3	> 4	4.4
**H22**	0.3	3.35±0.62	4.4
**H23**	1.0	2.97±0.26	4.6
**H24**	< 0.1	2.37±0.26	4.6
**H25**	0.1	2.98±0.35	5.4
**H26**	< 0.1	> 4	5.4
**H27**	< 0.1	> 4	5.2
**H28**	< 0.1	> 4	5.2

[a] IC_{50} values are shown as the mean from two or three separate experiments. [b] cLogP values were calculated using ChemDraw software and are subject to an inherent uncertainty of approximately ± 0.5 log units; therefore, values are reported with one decimal place.

Section S1. Kinase inhibitory activity of Compound **H24**. Figure S1. Dose–response curve of **H24** for PLK4 kinase inhibition. Figure S2. Dose–response curve of **H24** for Aurora A kinase inhibitory activity.

Section S2. *In vitro* anti-proliferative activity of Compound **H24**. Figure S3. Dose–response curve of **H24** for *in vitro* antiproliferative activity in MCF-7 cells. Figure S4. Dose–response curve of **H24** for *in vitro* antiproliferative activity in IMR-32 cells. Figure S5. Dose–response curve of **H24** for *in vitro* antiproliferative activity in A549 cells. Figure S6. Dose–response curve of **H24** for *in vitro* antiproliferative activity in HEK-293T cells.

Section S3. Mean IC_{50} (95% CI) of compounds. Table S1. IC_{50} values and 95% confidence intervals from independent experiments.

Section S4. cLogP data of the compound. Table S2. Predicted cLogP data for compounds **H13–H28**.

Section S5. Key characterization data for compounds **H1–H28**.

Section S6. Spectroscopic data of key compounds.

S5. Key characterization data for compounds **H1–H28**.

Dimethyl[2-({2-[(4-morpholin-4-ylphenyl)amino]quinazolin-7-yl}amino)phenyl]phosphine oxide (**H1**).

Pale yellow powdery solid (20.3 mg, 30.3% yield). m.p. 186.3 - 187.1 °C. ^{1}H NMR (600 MHz, DMSO-d_6) δ 9.62 (s, 1H), 9.40 (s, 1H), 8.94 (s, 1H), 7.76 (d, *J* = 8.8 Hz, 2H), 7.71 (d, *J* = 8.5 Hz, 1H), 7.66 (dd, *J* = 13.6, 7.3 Hz, 1H), 7.58 (d, *J* = 3.9 Hz, 2H), 7.20 – 7.16 (m, 1H), 6.95 (d, *J* = 8.7 Hz, 2H), 6.91 (d, *J* = 9.1 Hz, 2H), 3.75 – 3.72 (m, 4H), 3.04 – 3.02 (m, 4H), 1.74 (d, *J* = 13.5 Hz, 6H). ^{13}C NMR (151 MHz, DMSO-d_6) δ 159.35, 156.96, 152.37, 148.16, 145.40, 144.22, 132.65, 132.17, 131.18 (d, *J* = 10.2 Hz), 128.77, 123.09 (d, *J* = 93.6 Hz), 121.79 (d, *J* = 11.7 Hz), 120.18 (d, *J* = 6.6 Hz), 119.32 (2C), 115.17, 115.04 (2C), 114.72, 104.87, 65.56 (2C), 48.70 (2C), 17.52, 17.04. HRMS (ESI, *m/z*) calcd for $C_{26}H_{28}N_5NaO_2P$ [M + Na]$^+$, 496.1873; found 496.1924. Retention time = 2.555 min, HPLC purity = 98.4%.

Dimethyl[3-({2-[(4-morpholin-4-ylphenyl)amino]quinazolin-7-yl}amino)phenyl]phosphine oxide (**H2**).

Pale yellow powdery solid (22.0 mg, 33.3% yield). m.p. 171.6 - 173.3 °C. ^{1}H NMR (600 MHz, DMSO-d_6) δ 9.38 (s, 1H), 9.05 (s, 1H), 8.90 (s, 1H), 7.75 (d, *J* = 8.6 Hz, 2H), 7.69 (d, *J* = 8.6 Hz, 1H), 7.64 (d, *J* = 12.5 Hz, 1H), 7.53 – 7.50 (m, 1H), 7.46 (d, *J* = 8.2 Hz, 1H), 7.39 (dd, *J* = 11.1, 7.4 Hz, 1H), 7.03 – 7.01 (m, 2H), 6.90 (d, *J* = 9.1 Hz, 2H), 3.75 – 3.73 (m, 4H), 3.04 – 3.02 (m, 4H), 1.67 (d, *J* = 13.3 Hz, 6H). ^{13}C NMR (151 MHz, DMSO-d_6) δ 160.20, 158.05, 153.56, 149.46, 146.43, 141.95 (d, *J* = 12.9 Hz), 137.54, 133.84, 130.19 (d, *J* = 13.3 Hz), 129.62, 123.50, 122.57, 121.43, 120.39 (2C), 116.11 (2C), 115.76, 115.51, 104.74, 66.66 (2C), 49.82 (2C), 18.42, 17.95. HRMS (ESI, *m/z*) calcd for $C_{26}H_{28}N_5NaO_2P$ [M + Na]$^+$, 496.1873; found 496.1896. Retention time = 4.017 min, HPLC purity = 95.1%.

Methyl 2-((2-((4-morpholinophenyl)amino)quinazolin-6-yl)amino)benzoate (**H3**).

Pale yellow powdery solid (20 mg, 54.3% yield). m.p. 196.7 - 199.0 °C. ^{1}H NMR (600 MHz, DMSO-d_6) δ 9.46 (s, 1H), 9.42 (s, 1H), 8.99 (s, 1H), 7.95 (dd, *J* = 8.0, 1.1 Hz, 1H), 7.76 (dd, *J* = 12.5, 8.7 Hz, 3H), 7.65 – 7.56 (m, 2H), 7.18 (d, *J* = 1.8 Hz, 1H), 7.14 (dd, *J* = 8.6, 2.1 Hz, 1H), 7.07

(ddd, J = 8.1, 6.7, 1.7 Hz, 1H), 6.91 (d, J = 9.1 Hz, 2H), 3.85 (s, 3H), 3.77 – 3.69 (m, 4H), 3.08 – 2.99 (m, 4H). ^{13}C NMR (151 MHz, DMSO-d_6) δ 166.89, 159.55, 156.93, 152.19, 146.80, 145.44, 142.60, 133.55, 132.59, 130.85, 128.64, 120.34, 119.37 (2C), 118.24, 116.29, 115.99, 115.24, 115.03 (2C), 107.29, 65.57 (2C), 51.60, 48.69 (2C). HRMS (ESI, *m/z*) calcd for $C_{26}H_{24}N_5O_3$ [M - H]$^-$, 454.1879; found 454.1891. Retention time = 2.615 min, HPLC purity = 95.4%.

Methyl 3-((2-((4-morpholinophenyl)amino)quinazolin-6-yl)amino)benzoate (**H4**).

Pale yellow powdery solid (23 mg, 48.7% yield). m.p. 203.2 - 204.5 °C. ^{1}H NMR (600 MHz, DMSO-d_6) δ 9.39 (s, 1H), 9.08 (s, 1H), 8.92 (s, 1H), 7.83 (t, J = 2.1 Hz, 1H), 7.75 (d, J = 8.5 Hz, 2H), 7.71 (d, J = 8.5 Hz, 1H), 7.61 (dt, J = 7.5, 1.5 Hz, 1H), 7.57 (dt, J = 8.4, 1.5 Hz, 1H), 7.52 (t, J = 7.8 Hz, 1H), 7.04 – 7.00 (m, 2H), 6.93 – 6.88 (m, 2H), 3.87 (s, 3H), 3.74 (dd, J = 6.0, 3.6 Hz, 4H), 3.07 – 2.99 (m, 4H). ^{13}C NMR (151 MHz, DMSO-d_6) δ 168.05, 160.71, 158.08, 153.34, 147.94, 146.59, 143.76, 134.70, 133.74, 132.00, 129.79, 121.49, 120.51 (2C), 119.38, 117.44, 117.14, 116.39, 116.17 (2C), 108.44, 66.71 (2C), 52.75, 49.84 (2C). HRMS (ESI, *m/z*) calcd for $C_{26}H_{24}N_5O_3$ [M - H]$^-$, 454.1879; found 454.1884. Retention time = 2.330 min, HPLC purity = 98.9%.

N^2-[4-(Morpholin-4-yl)phenyl]-N^7-(4-methylphenyl)quinazoline-2,7-diamine (**H5**)

Pale yellow powdery solid (26.3 mg, 48.0% yield). m.p. 228.2 - 229.4 °C. ^{1}H NMR (400 MHz, DMSO-d_6) δ 9.29 (s, 1H), 8.83 (s, 1H), 8.72 (s, 1H), 7.75 (d, J = 8.6 Hz, 2H), 7.62 (d, J = 8.7 Hz, 1H), 7.20 (d, J = 8.4 Hz, 2H), 7.17 (d, J = 8.5 Hz, 2H), 6.94 (dd, J = 8.7, 2.2 Hz, 1H), 6.91 (s, 1H), 6.90 – 6.88 (m, 2H), 3.75 – 3.71 (m, 4H), 3.05 – 3.00 (m, 4H), 2.30 (s, 3H). ^{13}C NMR (151 MHz, DMSO-d_6) δ 158.80, 156.94, 152.61, 149.55, 145.26, 137.75, 132.84, 131.18, 129.24 (2C), 128.35, 120.35 (2C), 119.21 (2C), 115.05 (2C), 114.49, 113.93, 102.18, 65.57 (2C), 48.74 (2C), 19.86. HRMS (ESI, *m/z*) calcd for $C_{25}H_{24}N_5O$ [M - H]$^-$, 410.2059; found 410.2006. Retention time = 9.867 min, HPLC purity = 97.1%.

N^2-[4-(Morpholin-4-yl)phenyl]-N^7-(3-methylphenyl)quinazoline-2,7-diamine (**H6**)

Pale yellow powdery solid (46.4 mg, 37.5% yield). m.p. 190.6 - 191.3 °C. ^{1}H NMR (600 MHz, DMSO-d_6) δ 9.32 (s, 1H), 8.86 (s, 1H), 8.78 (s, 1H), 7.74 (d, J = 8.6 Hz, 2H), 7.65 (d, J = 8.7 Hz, 1H), 7.26 (m, 1H), 7.09 – 7.06 (m, 2H), 6.98 (dd, J = 8.7, 2.2 Hz, 1H), 6.95 (d, J = 2.1 Hz, 1H), 6.90 (d, J = 9.0 Hz, 2H), 6.88 (d, J = 7.5 Hz, 1H), 3.75 – 3.72 (m, 4H), 3.04 – 3.02 (m, 4H), 2.32 (s, 3H). ^{13}C NMR (151 MHz, DMSO-d_6) δ 160.00, 158.02, 153.62, 150.19, 146.39, 141.50, 139.19,

133.86, 129.69, 129.47, 123.77, 121.37 (2C), 120.34, 117.87, 116.14 (2C), 115.72, 115.17, 104.05, 66.65 (2C), 49.82 (2C), 21.63. HRMS (ESI, *m/z*) calcd for $C_{25}H_{24}N_5O$ [M - H]$^-$, 410.2059; found 410.1994. Retention time = 10.093 min, HPLC purity = 100.0%.

N^7-(4-Bromophenyl)-N^2-[4-(morpholin-4-yl)phenyl]quinazoline-2,7-diamine (**H7**)

Pale yellow powdery solid (23.0 mg, 24.5% yield). m.p. 206.0 - 207.5 °C. ^{1}H NMR (400 MHz, DMSO-d_6) δ 9.36 (s, 1H), 8.94 (s, 1H), 8.89 (s, 1H), 7.76 (d, J = 9.0 Hz, 2H), 7.68 (d, J = 8.6 Hz, 1H), 7.53 (d, J = 8.8 Hz, 2H), 7.23 (d, J = 8.8 Hz, 2H), 6.99 (dd, J = 11.2, 2.6 Hz, 2H), 6.91 (d, J = 9.1 Hz, 2H), 3.75 – 3.72 (m, 4H), 3.05 – 3.02 (m, 4H). ^{13}C NMR (151 MHz, DMSO-d_6) δ 159.11, 156.96, 152.47, 148.19, 145.35, 140.07, 132.73, 131.55 (2C), 128.53, 121.03 (2C), 119.29 (2C), 115.04 (2C), 114.78, 114.41, 112.72, 103.68, 65.57 (2C), 48.72 (2C). HRMS (ESI, *m/z*) calcd for $C_{24}H_{22}BrN_5NaO$ [M + Na]$^+$, 498.0900; found 498.0923. Retention time = 16.133 min, HPLC purity = 99.5%.

N^7-(3-Bromophenyl)-N^2-[4-(morpholin-4-yl)phenyl]quinazoline-2,7-diamine (**H8**)

Pale yellow powdery solid (27.3 mg, 28.8% yield). m.p. 210.5 - 212.3 °C. ^{1}H NMR (600 MHz, DMSO-d_6) δ 9.40 (s, 1H), 9.01 (s, 1H), 8.92 (s, 1H), 7.75 (d, J = 8.5 Hz, 2H), 7.70 (d, J = 8.6 Hz, 1H), 7.39 (s, 1H), 7.34 – 7.28 (m, 2H), 7.19 (d, J = 7.5 Hz, 1H), 7.02 (s, 1H), 7.01 (dd, J = 8.6, 2.2 Hz, 1H), 6.91 (d, J = 9.1 Hz, 2H), 3.76 – 3.71 (m, 4H), 3.06 – 3.01 (m, 4H). ^{13}C NMR (151 MHz, DMSO-d_6) δ 160.34, 158.05, 153.48, 148.93, 146.50, 143.65, 133.69, 131.81, 129.72, 124.94, 122.64, 122.10, 120.47 (2C), 118.54, 116.13 (2C), 115.89, 115.67, 105.47, 66.64 (2C), 49.78 (2C). HRMS (ESI, *m/z*) calcd for $C_{24}H_{22}BrN_5NaO$ [M + Na]$^+$, 498.0900; found 498.0916. Retention time = 16.992 min, HPLC purity = 99.3%.

N^7-(4-Fluorophenyl)-N^2-[4-(morpholin-4-yl)phenyl]quinazoline-2,7-diamine (**H9**)

Yellow powdery solid (67.3 mg, 52.3% yield). m.p. 194.8 - 195.8 °C. ^{1}H NMR (600 MHz, DMSO-d_6) δ 9.32 (s, 1H), 8.86 (s, 1H), 8.79 (s, 1H), 7.76 - 7.72 (m, 2H). 7.65 (d. J = 8.7 Hz, 1H), 7.29 (ddt, J= 8.4, 5.8, 2.9 Hz 2H), 7.26 - 7.20 (m, 2H), 6.96 - 6.85 (m, 4H), 3.76 - 3.70 (m, 4H), 3.06 - 2.99 (m, 4H). ^{13}C NMR (151 MHz, DMSO-d_6) δ 160.00, 158.39 (d, J = 239.4 Hz), 158.04, 153.67, 150.52, 146.37, 137.83 (d, J = 2.9 Hz), 133.86, 129.55, 123.24 (d, J = 8.0 Hz, 2C), 120.33 (2C), 116.54 (d, J = 22.6 Hz, 2C), 116.12 (2C), 115.43, 115.14, 103.38, 66.65 (2C), 49.81 (2C). HRMS (ESI, *m/z*) calcd for $C_{24}H_{21}FN_5O$ [M - H]$^-$, 414.1730; found 414.1769. Retention time =

8.747 min, HPLC purity = 95.8%.

N^7-(3- fluorophenyl)- *N^2*-[4-(morpholin-4-yl)phenyl]quinazoline-2,7-diamine (**H10**)

Pale yellow powdery solid (25.0 mg, 45.7% yield). m.p. 214.5 - 216.5 °C. ^{1}H NMR (600 MHz, DMSO-d_6) δ 9.39 (s, 1H), 9.04 (s, 1H), 8.92 (s, 1H), 7.76 (d, *J* = 8.8 Hz, 2H), 7.70 (d, *J* = 8.7 Hz, 1H), 7.39(m, 1H), 7.14 - 7.08 (m, 1H), 7.04 (ddd, *J* = 18.3, 8.1, 2.2 Hz, 3H), 6.93 - 6.89 (m, 2H), 6.85 - 6.80 (m, 1H) 3.79 - 3.69 (m, 4H), 3.08 - 3.00 (m, 4H). ^{13}C NMR (151 MHz, DMSO-d_6) δ 162.27 (d, *J* = 242.5 Hz), 159.23, 156.99, 152.45, 147.91, 145.39, 142.79 (d, *J* = 10.4 Hz), 132.71, 130.41 (d, *J* = 9.9 Hz), 128.57, 119.32 (2C), 115.07 (2C), 114.88, 114.58, 114.55 (d, *J* = 2.6 Hz), 107.71 (d, *J* = 21.0 Hz), 105.20 (d, *J* = 24.2 Hz), 104.50, 65.59 (2C), 48.73 (2C). HRMS (ESI, *m/z*) calcd for $C_{24}H_{21}FN_5O$ [M - H]$^-$, 414.1730; found 414.1778. Retention time = 9.907 min, HPLC purity = 96.6%.

N^7-(4-Chlorophenyl)- *N^2*-[4-(morpholin-4-yl)phenyl]quinazoline-2,7-diamine (**H11**)

Pale yellow powdery solid (19.1 mg, 19.7% yield). m.p. 195.0 - 196.0 °C. ^{1}H NMR (400 MHz, DMSO-d_6) δ 9.35 (s, 1H), 8.94 (s, 1H), 8.89 (s, 1H), 7.76 (d, *J* = 9.0 Hz, 2H), 7.68 (d, *J* = 9.4 Hz, 1H), 7.41 (d, *J* = 8.8 Hz, 2H), 7.28 (d, *J* = 8.8 Hz, 2H), 6.98 (d, *J* = 7.7 Hz, 2H), 6.91 (d, *J* = 9.1 Hz, 2H), 3.76 – 3.72 (m, 4H), 3.05 – 3.01 (m, 4H). ^{13}C NMR (151 MHz, DMSO-d_6) δ 159.07, 156.94, 152.45, 148.33, 145.32, 139.61, 132.70, 128.64 (2C), 128.50, 124.94, 120.74 (2C), 119.27 (2C), 115.02 (2C), 114.69, 114.34, 103.49, 65.55 (2C), 48.70 (2C). HRMS (ESI, *m/z*) calcd for $C_{24}H_{22}ClN_5NaO$ [M + Na]$^+$, 454.1405; found 454.1436. Retention time = 6.907 min, HPLC purity = 95.9%.

N^7-(3-Chlorophenyl)- *N^2*-[4-(morpholin-4-yl)phenyl]quinazoline-2,7-diamine (**H12**)

Pale yellow powdery solid (18.0 mg, 18.5% yield). m.p. 211.6 - 213.0 °C. ^{1}H NMR (400 MHz, DMSO-d_6) δ 9.39 (s, 1H), 9.01 (s, 1H), 8.92 (s, 1H), 7.76 (d, *J* = 8.7 Hz, 2H), 7.71 (d, *J* = 8.6 Hz, 1H), 7.38 (m, 1H), 7.27 – 7.23 (m, 2H), 7.07 – 7.03 (m, 2H), 7.03 – 7.00 (m, 1H), 6.91 (d, *J* = 9.0 Hz, 2H), 3.75 – 3.71 (m, 4H), 3.06 – 3.02 (m, 4H). ^{13}C NMR (151 MHz, DMSO-d_6) δ 159.25, 156.99, 152.43, 147.85, 145.40, 142.47, 133.10, 132.69, 130.44, 128.61, 120.93, 119.33 (2C), 118.11, 117.08, 115.05 (2C), 114.84, 114.60, 104.48, 65.58 (2C), 48.72 (2C). HRMS (ESI, *m/z*) calcd for $C_{24}H_{22}ClN_5NaO$ [M + Na]$^+$, 454.1405; found 454.1426. Retention time = 14.735 min, HPLC purity = 96.2%.

4-Methyl-*N*-[2-({4-(morpholin-4-yl)phenyl}amino)quinazolin-7-yl]benzenesulfonamide (**H13**)

pale yellow powdery solid (47.1 mg, 37.8% yield). m.p. 182.6 - 183.8 °C. ^{1}H NMR (400 MHz, DMSO-d_6) δ 10.86 (s, 1H), 9.54 (s, 1H), 9.01 (s, 1H), 7.76 (s, 1H), 7.74 (s, 2H), 7.73 – 7.69 (m, 2H), 7.37 (d, *J* = 8.1 Hz, 2H), 7.20 (d, *J* = 2.1 Hz, 1H), 7.09 (dd, *J* = 8.6, 2.1 Hz, 1H), 6.94 (d, *J* = 9.1 Hz, 2H), 3.78 – 3.72 (m, 4H), 3.09 – 3.03 (m, 4H), 2.31 (s, 3H). ^{13}C NMR (151 MHz, DMSO-d_6) δ 161.52, 157.92, 152.35, 146.78, 144.28, 143.75, 136.82, 133.21, 130.39 (2C), 129.77, 127.16 (2C), 120.70 (2C), 117.14, 116.10 (2C), 115.86, 111.58, 66.63 (2C), 49.70 (2C), 21.40. HRMS (ESI, *m/z*) calcd for $C_{25}H_{25}N_5NaO_3S$ $[M + Na]^+$, 498.1570; found 498.1552. Retention time = 8.331 min, HPLC purity = 97.3%.

3-Methyl-*N*-[2-({4-(morpholin-4-yl)phenyl}amino)quinazolin-7-yl]benzenesulfonamide (**H14**)

Pale yellow powdery solid (47.4 mg, 37.5% yield). m.p. 227.9 - 229.5 °C. ^{1}H NMR (400 MHz, DMSO-d_6) δ 10.90 (s, 1H), 9.55 (s, 1H), 9.02 (s, 1H), 7.74 (d, *J* = 4.0 Hz, 2H), 7.71 (s, 2H), 7.66 (d, *J* = 7.1 Hz, 1H), 7.45 (d, *J* = 7.5 Hz, 2H), 7.21 (s, 1H), 7.11 (d, *J* = 8.6 Hz, 1H), 6.94 (d, *J* = 8.6 Hz, 2H), 3.77 – 3.73 (m, 4H), 3.08 – 3.03 (m, 4H), 2.35 (s, 3H). ^{13}C NMR (151 MHz, DMSO-d_6) δ 161.54, 157.91, 152.32, 146.76, 143.68, 139.76, 139.71, 134.45, 133.23, 129.81 (2C), 127.28, 124.30, 120.70 (2C), 117.14, 116.11 (2C), 115.82, 111.57, 66.62 (2C), 49.70 (2C), 21.26. HRMS (ESI, *m/z*) calcd for $C_{25}H_{24}N_5O_3S$ $[M - H]^-$, 474.1678; found 474.1617. Retention time = 8.487 min, HPLC purity = 99.6%.

4-Methoxy-*N*-[2-({4-(morpholin-4-yl)phenyl}amino)quinazolin-7-yl]benzenesulfonamide (**H15**)

Pale yellow powdery solid (63.3 mg, 49.2% yield). m.p. 204.8 - 205.8 °C. ^{1}H NMR (400 MHz, DMSO-d_6) δ 10.81 (s, 1H), 9.56 (s, 1H), 9.02 (s, 1H), 7.81 (d, *J* = 8.8 Hz, 2H), 7.75 (s, 1H), 7.72 (d, *J* = 8.9 Hz, 2H), 7.22 (s, 1H), 7.11 (d, *J* = 6.2 Hz, 2H), 7.08 (s, 1H), 6.94 (d, *J* = 9.0 Hz, 2H), 3.77 (s, 3H), 3.76 – 3.73 (m, 4H), 3.08 – 3.04 (m, 4H). ^{13}C NMR (151 MHz, DMSO-d_6) δ 163.17, 161.49, 157.92, 152.38, 146.74, 143.87, 133.27, 131.26, 129.73, 129.40 (2C), 120.67 (2C), 117.10, 116.10 (2C), 115.85, 115.08 (2C), 111.46, 66.63 (2C), 56.10, 49.71 (2C). HRMS (ESI, *m/z*) calcd for $C_{25}H_{25}N_5NaO_4S$ $[M + Na]^+$, 514.1519; found 514.1540. Retention time = 6.664 min, HPLC purity = 99.5%.

3-Methoxy-*N*-[2-({4-(morpholin-4-yl)phenyl}amino)quinazolin-7-yl]benzenesulfonamide (**H16**)

Pale yellow powdery solid (102.0 mg, 53.1% yield). m.p. 233.9 - 235.2 °C. ^{1}H NMR (400 MHz,

DMSO-d_6) δ 10.93 (s, 1H), 9.57 (s, 1H), 9.03 (s, 1H), 7.76 (s, 1H), 7.75 – 7.72 (m, 2H), 7.50 (m, 1H), 7.44 (d, J = 7.9 Hz, 1H), 7.38 (m, 1H), 7.24 (d, J = 2.1 Hz, 1H), 7.21 – 7.17 (m, 1H), 7.12 (dd, J = 8.7, 2.1 Hz, 1H), 6.94 (d, J = 9.0 Hz, 2H), 3.78 (s, 3H), 3.77 – 3.73 (m, 4H), 3.08 – 3.04 (m, 4H). ^{13}C NMR (151 MHz, DMSO-d_6) δ 161.56, 159.88, 157.92, 152.31, 146.74, 143.63, 140.92, 133.25, 131.23, 129.81, 120.68, 119.43, 119.19 (2C), 117.22, 116.10, 115.93, 112.26 (2C), 111.80, 66.62 (2C), 56.06, 49.70 (2C). HRMS (ESI, *m/z*) calcd for $C_{25}H_{24}N_5O_4S$ [M - H]$^-$, 490.1627; found 490.1569. Retention time = 7.616 min, HPLC purity = 99.4%.

4-Bromo-*N*-[2-({4-(morpholin-4-yl)phenyl}amino)quinazolin-7-yl]benzenesulfonamide (**H17**)

Pale yellow powdery solid (35.8 mg, 12.5% yield). m.p. 224.5 - 226.1 °C. ^{1}H NMR (600 MHz, DMSO-d_6) δ 11.02 (s, 1H), 9.59 (s, 1H), 9.04 (s, 1H), 7.81 (d, J = 8.8 Hz, 2H), 7.78 (d, J = 8.8 Hz, 2H), 7.75 (s, 1H), 7.74 (t, J = 4.3 Hz, 2H), 7.22 (d, J = 2.0 Hz, 1H), 7.09 (dd, J = 8.6, 2.1 Hz, 1H), 6.95 (d, J = 9.1 Hz, 2H), 3.77 – 3.73 (m, 4H), 3.08 – 3.04 (m, 4H). ^{13}C NMR (151 MHz, DMSO-d_6) δ 161.62, 157.94, 152.34, 146.80, 143.31, 138.94, 133.18, 133.11 (2C), 129.94, 129.12 (2C), 127.76, 120.75 (2C), 116.10 (2C), 115.94, 112.08, 66.62 (2C), 49.69 (2C). HRMS (ESI, *m/z*) calcd for $C_{24}H_{21}BrN_5O_3S$ [M - H]$^-$, 538.0627; found 538.0579. Retention time = 12.827 min, HPLC purity = 99.4%.

3-Bromo-*N*-[2-({4-(morpholin-4-yl)phenyl}amino)quinazolin-7-yl]benzenesulfonamide (**H18**)

Pale yellow powdery solid (80.3 mg, 28.5% yield). m.p. 233.2 - 234.0 °C. ^{1}H NMR (600 MHz, DMSO-d_6) δ 11.05 (s, 1H), 9.61 (s, 1H), 9.06 (s, 1H), 8.02 (s, 1H), 7.86 (t, J = 6.3 Hz, 2H), 7.77 (d, J = 8.8 Hz, 2H), 7.74 (s, 1H), 7.55 (m, 1H), 7.23 (d, J = 2.1 Hz, 1H), 7.12 (dd, J = 8.7, 2.2 Hz, 1H), 6.95 (d, J = 8.6 Hz, 2H), 3.77 – 3.73 (m, 4H), 3.08 – 3.04 (m, 4H). ^{13}C NMR (151 MHz, DMSO-d_6) δ 161.70, 157.90, 152.22, 146.71, 143.20, 141.63, 136.71, 133.21, 132.29, 130.00, 129.46, 126.20, 122.75 (2C), 120.76, 117.37, 116.15 (2C), 115.98, 112.12, 66.60 (2C), 49.73 (2C). HRMS (ESI, *m/z*) calcd for $C_{24}H_{21}BrN_5O_3S$ [M - H]$^-$, 538.0627; found 538.0582. Retention time = 12.047 min, HPLC purity = 99.6%.

4-Chloro-*N*-[2-({4-(morpholin-4-yl)phenyl}amino)quinazolin-7-yl]benzenesulfonamide (**H19**)

Pale yellow powdery solid (35.4 mg, 27.4% yield). m.p. 228.4 - 229.1 °C. ^{1}H NMR (600 MHz, DMSO-d_6) δ 11.04 (s, 1H), 9.58 (s, 1H), 9.04 (s, 1H), 7.75 (d, J = 8.8 Hz, 2H), 7.73 – 7.69 (m, 2H), 7.67 – 7.63 (m, 2H), 7.54 – 7.50 (m, 1H), 7.22 (d, J = 2.1 Hz, 1H), 7.11 (dd, J = 8.6, 2.1 Hz, 1H),

6.94 (d, *J* = 8.9 Hz, 2H), 3.76 – 3.74 (m, 4H), 3.07 – 3.05 (m, 4H). ^{13}C NMR (151 MHz, DMSO-d_6) δ 160.51, 156.87, 151.29, 145.70, 142.26, 137.61, 137.49, 132.14, 129.10 (2C), 128.83, 127.97 (2C), 119.60 (2C), 116.27, 115.00 (2C), 114.87, 111.04, 65.56 (2C), 48.61 (2C). HRMS (ESI, *m/z*) calcd for $C_{24}H_{21}ClN_5O_3S$ [M - H]$^-$, 494.1132; found 494.1079. Retention time = 11.527 min, HPLC purity = 99.5%.

3-Chloro-*N*-[2-({4-(morpholin-4-yl)phenyl}amino)quinazolin-7-yl]benzenesulfonamide (**H20**)

Pale yellow powdery solid (27.0 mg, 21.1% yield). m.p. 231.4 - 232.2 °C. ^{1}H NMR (600 MHz, DMSO-d_6) δ 11.04 (s, 1H), 9.59 (s, 1H), 9.05 (s, 1H), 7.87 (s, 1H), 7.81 (d, *J* = 7.9 Hz, 1H), 7.75 (d, *J* = 8.6 Hz, 2H), 7.73 (d, *J* = 5.5 Hz, 2H), 7.62 (m, 1H), 7.21 (s, 1H), 7.11 (d, *J* = 6.5 Hz, 1H), 6.94 (d, *J* = 8.4 Hz, 2H), 3.76 – 3.73 (m, 4H), 3.07 – 3.04 (m, 4H). ^{13}C NMR (151 MHz, DMSO-d_6) δ 161.66, 157.98, 152.36, 146.82, 143.22, 141.60, 134.52, 133.86, 133.24, 132.15, 130.00, 126.69, 125.90, 120.68 (2C), 117.41, 116.10 (2C), 115.98, 112.27, 66.66 (2C), 49.70 (2C). HRMS (ESI, *m/z*) calcd for $C_{24}H_{22}ClN_5NaO_3S$ [M + Na]$^+$, 518.1024; found 518.1024. Retention time = 11.095 min, HPLC purity = 99.0%.

4-Fluoro-*N*-[2-({4-(morpholin-4-yl)phenyl}amino)quinazolin-7-yl]benzenesulfonamide (**H21**)

Pale yellow powdery solid (27.1 mg, 21.6% yield). m.p. 213.7 - 214.7 °C. 1H NMR (400 MHz, DMSO-d6) δ 10.95 (s, 1H), 9.57 (s, 1H), 9.03 (s, 1H), 7.96 – 7.90 (m, 2H), 7.74 (d, J = 8.7 Hz, 3H), 7.45 – 7.40 (m, 2H), 7.22 (d, J = 2.0 Hz, 1H), 7.10 (dd, J = 8.6, 2.1 Hz, 1H), 6.94 (d, J = 9.1 Hz, 2H), 3.77 – 3.73 (m, 4H), 3.09 – 3.03 (m, 4H). ^{13}C NMR (151 MHz, DMSO-d_6) δ 164.96 (d, *J* = 252.3 Hz), 161.58, 157.94, 152.37, 146.78, 143.45, 136.09 (d, *J* = 3.0 Hz), 133.22, 130.23 (d, *J* = 9.7 Hz, 2C), 129.87, 120.68 (2C), 117.30, 117.24 (d, *J* = 22.9 Hz, 2C), 116.09 (2C), 115.94, 112.00, 66.63 (2C), 49.69 (2C). HRMS (ESI, m/z) calcd for $C_{24}H_{21}FN_5O_3S$ [M - H]-, 478.1427; found 478.1363. Retention time = 7.587 min, HPLC purity = 99.2%.

3-Fluoro-*N*-[2-({4-(morpholin-4-yl)phenyl}amino)quinazolin-7-yl]benzenesulfonamide (**H22**)

Pale yellow powdery solid (46.8 mg, 36.9% yield). m.p. 223.5 - 224.9 °C. ^{1}H NMR (600 MHz, DMSO-d_6) δ 11.05 (s, 1H), 9.60 (s, 1H), 9.05 (s, 1H), 7.76 (d, *J* = 8.4 Hz, 2H), 7.74 – 7.70 (m, 2H), 7.69 – 7.63 (m, 2H), 7.54 – 7.50 (m, 1H), 7.23 (s, 1H), 7.13 (dd, *J* = 8.6, 2.1 Hz, 1H), 6.95 (d, *J* = 8.5 Hz, 2H), 3.77 – 3.73 (m, 4H), 3.09 – 3.03 (m, 4H). ^{13}C NMR (151 MHz, DMSO-d_6) δ 162.31 (d, *J* = 200.3 Hz), 161.32, 157.93, 152.31, 146.80, 143.24, 141.70 (d, *J* = 6.8 Hz), 133.17, 132.49

(d, J = 8.0 Hz), 129.95, 123.45 (d, J = 3.0 Hz), 121.08 (d, J = 21.0 Hz), 120.73 (2C), 117.37, 116.10 (2C), 115.98, 114.14 (d, J = 24.5 Hz), 112.21, 66.62 (2C), 49.69 (2C). HRMS (ESI, *m/z*) calcd for $C_{24}H_{21}FN_5O_3S$ [M - H]$^-$, 478.1427; found 478.1369. Retention time = 7.927 min, HPLC purity = 99.5%.

2,5-Difluoro-*N*-[2-({4-(morpholin-4-yl)phenyl}amino)quinazolin-7-yl]benzenesulfonamide (**H23**)

Pale yellow powdery solid (36.6 mg, 14.0% yield). m.p. 139.4 - 140.8 °C. ^{1}H NMR (600 MHz, DMSO-d_6) δ 11.40 (s, 1H), 9.59 (s, 1H), 9.05 (s, 1H), 7.81 – 7.78 (m, 1H), 7.76 (d, J = 8.7 Hz, 1H), 7.73 (d, J = 8.6 Hz, 2H), 7.63 – 7.59 (m, 1H), 7.55 – 7.51 (m, 1H), 7.20 (d, J = 2.1 Hz, 1H), 7.13 (dd, J = 8.6, 2.1 Hz, 1H), 6.94 (d, J = 9.1 Hz, 2H), 3.76 – 3.74 (m, 4H), 3.07 – 3.05 (m, 4H). ^{13}C NMR (151 MHz, DMSO-d_6) δ 161.67, 157.95, 157.69 (dd, J = 248.4, 5.0 Hz), 154.85 (dd, J = 253.2, 3.9 Hz), 152.28, 146.83, 142.73, 133.13, 130.00, 128.37 (dd, J = 15.8, 7.5 Hz), 125.06, 123.52 (dd, J = 23.6, 9.1 Hz), 120.77 (2C), 120.04 (dd, J = 22.9, 9.3 Hz), 117.61 – 117.25 (m), 116.09 (2C), 115.66, 111.88, 66.61 (2C), 49.68 (2C). HRMS (ESI, *m/z*) calcd for $C_{24}H_{20}F_2N_5O_3S$ [M - H]$^-$, 496.1333; found 496.1292. Retention time = 7.902 min, HPLC purity = 99.6%.

2,4-Difluoro-*N*-[2-({4-(morpholin-4-yl)phenyl}amino)quinazolin-7-yl]benzenesulfonamide (**H24**)

Pale yellow powdery solid (56.7 mg, 21.6% yield). m.p. 205.2 - 207.1 °C. ^{1}H NMR (600 MHz, DMSO-d6) δ 11.32 (s, 1H), 9.58 (s, 1H), 9.04 (s, 1H), 8.07 – 8.02 (m, 1H), 7.75 (d, J = 9.1 Hz, 2H), 7.72 (s, 1H), 7.57 – 7.52 (m, 1H), 7.33 – 7.29 (m, 1H), 7.20 (d, J = 2.2 Hz, 1H), 7.11 (dd, J = 8.7, 2.1 Hz, 1H), 6.95 (d, J = 9.0 Hz, 2H), 3.77 – 3.73 (m, 4H), 3.08 – 3.04 (m, 4H). ^{13}C NMR (151 MHz, DMSO-d_6) δ 165.71, 165.64, 164.02, 163.94, 160.54, 159.31, 159.22, 157.60, 157.51, 156.90, 151.29, 145.73, 141.87, 132.16, 132.02, 131.95, 128.85, 122.99, 122.96, 122.90, 122.87, 119.63, 116.22, 115.02, 114.54, 112.13, 112.11, 111.98, 111.96, 110.56, 105.93, 105.76, 105.59, 65.58, 48.63.HRMS (ESI, *m/z*) calcd for $C_{24}H_{20}F_2N_5O_3S$ [M - H]$^-$, 496.1333; found 496.1282. Retention time = 7.830 min, HPLC purity = 99.3%.

2,5-Dichloro-*N*-[2-({4-(morpholin-4-yl)phenyl}amino)quinazolin-7-yl]benzenesulfonamide (**H25**)

Pale yellow powdery solid (30.3 mg, 14.6% yield). m.p. 152.7 - 153.9 °C. ^{1}H NMR (400 MHz, DMSO-d_6) δ 11.44 (s, 1H), 9.60 (s, 1H), 9.04 (s, 1H), 8.15 (d, J = 2.5 Hz, 1H), 7.77 (d, J = 2.3 Hz, 1H), 7.75 (d, J = 2.6 Hz, 2H), 7.70 (m, 2H), 7.17 – 7.08 (m, 2H), 6.94 (d, J = 8.6 Hz, 2H), 3.78 – 3.73 (m, 4H), 3.10 – 3.02 (m, 4H). ^{13}C NMR (151 MHz, DMSO-d_6) δ 161.69, 157.90, 152.15,

146.70, 142.54, 138.08, 135.34, 134.33, 133.22, 131.37, 130.09, 130.06, 120.75 (2C), 117.23, 116.14 (2C), 115.39, 111.37, 66.59 (2C), 49.74 (2C). HRMS (ESI, *m/z*) calcd for $C_{24}H_{20}Cl_2N_5O_3S$ $[M - H]^-$, 528.0742; found 528.0671. Retention time = 14.293 min, HPLC purity = 99.0%.

2,4-Dichloro-*N*-[2-({4-(morpholin-4-yl)phenyl}amino)quinazolin-7-yl]benzenesulfonamide (**H26**)

Pale yellow powdery solid (35.1 mg, 12.7% yield). m.p. 246.6 - 247.1 °C. ^1H NMR (600 MHz, DMSO-d_6) δ 11.37 (s, 1H), 9.58 (s, 1H), 9.03 (s, 1H), 8.17 (d, *J* = 8.6 Hz, 1H), 7.87 (d, *J* = 2.1 Hz, 1H), 7.74 (d, *J* = 8.7 Hz, 2H), 7.72 (s, 1H), 7.67 (dd, *J* = 8.6, 2.1 Hz, 1H), 7.16 (s, 1H), 7.10 (dd, *J* = 8.7, 2.2 Hz, 1H), 6.94 (d, *J* = 9.0 Hz, 2H), 3.76 – 3.74 (m, 4H), 3.07 – 3.05 (m, 4H). ^{13}C NMR (151 MHz, DMSO-d_6) δ 161.67, 157.93, 152.24, 146.72, 142.73, 139.61, 135.61, 133.48, 133.24, 132.51, 132.10, 130.00, 128.61, 120.77 (2C), 117.21, 116.17 (2C), 115.36, 111.31, 66.61 (2C), 49.75 (2C). HRMS (ESI, *m/z*) calcd for $C_{24}H_{20}Cl_2N_5O_3S$ $[M - H]^-$, 528.0742; found 528.0697. Retention time = 16.753 min, HPLC purity = 99.0%.

2,5-Dimethyl-*N*-[2-({4-(morpholin-4-yl)phenyl}amino)quinazolin-7-yl]benzenesulfonamide (**H27**)

Pale yellow powdery solid (124.1 mg, 48.8% yield). m.p. 260.8 - 262.4 °C. ^1H NMR (600 MHz, DMSO-d_6) δ 11.04 (s, 1H), 9.56 (s, 1H), 9.00 (s, 1H), 7.89 (s, 1H), 7.75 (d, *J* = 8.4 Hz, 2H), 7.72 (d, *J* = 8.6 Hz, 1H), 7.32 (d, *J* = 7.8 Hz, 1H), 7.25 (d, *J* = 7.7 Hz, 1H), 7.14 (d, *J* = 2.0 Hz, 1H), 7.09 (dd, *J* = 8.6, 2.1 Hz, 1H), 6.93 (d, *J* = 9.1 Hz, 2H), 3.76 – 3.74 (m, 4H), 3.07 – 3.04 (m, 4H), 2.57 (s, 3H), 2.35 (s, 3H). ^{13}C NMR (151 MHz, DMSO-d_6) δ 161.44, 157.87, 152.27, 146.70, 143.47, 137.36, 136.38, 134.48, 134.19, 133.32, 133.18, 130.27, 129.77, 120.64 (2C), 116.83, 116.10 (2C), 115.15, 110.42, 66.61 (2C), 49.73 (2C), 20.79, 19.63. HRMS (ESI, *m/z*) calcd for $C_{26}H_{27}N_5NaO_3S$ $[M + Na]^+$, 512.1727; found 512.1765. Retention time = 12.234 min, HPLC purity = 99.8%.

2,4-Dimethyl-*N*-[2-({4-(morpholin-4-yl)phenyl}amino)quinazolin-7-yl]benzenesulfonamide (**H28**)

Pale yellow powdery solid (94.2 mg, 37.0% yield). m.p. 225.4 - 227.0 °C. ^1H NMR (600 MHz, DMSO-d_6) δ 11.01 (s, 1H), 9.55 (s, 1H), 9.00 (s, 1H), 7.90 (d, *J* = 8.1 Hz, 1H), 7.73 (d, *J* = 8.6 Hz, 2H), 7.71 (d, *J* = 8.7 Hz, 1H), 7.20 (d, *J* = 8.2 Hz, 1H), 7.18 (s, 1H), 7.14 (s, 1H), 7.09 (dd, *J* = 8.7, 2.1 Hz, 1H), 6.94 (d, *J* = 8.5 Hz, 2H), 3.76 – 3.73 (m, 4H), 3.07 – 3.04 (m, 4H), 2.59 (s, 3H), 2.26 (s, 3H). ^{13}C NMR (151 MHz, DMSO-d_6) δ 161.44, 157.92, 152.36, 146.71, 144.29, 143.58, 137.14,

134.73, 133.80, 133.26, 130.11, 129.78, 127.33, 120.70 (2C), 116.86, 116.11 (2C), 115.17, 110.43, 66.60 (2C), 49.70 (2C), 21.12, 20.01. HRMS (ESI, *m/z*) calcd for $C_{26}H_{26}N_5O_3S$ $[M - H]^-$, 488.1835; found 488.1787. Retention time = 11.760 min, HPLC purity = 95.6%.

S6. Spectroscopic data of key compounds

^{1}H-NMR and HRMS spectra of compound **M2**.

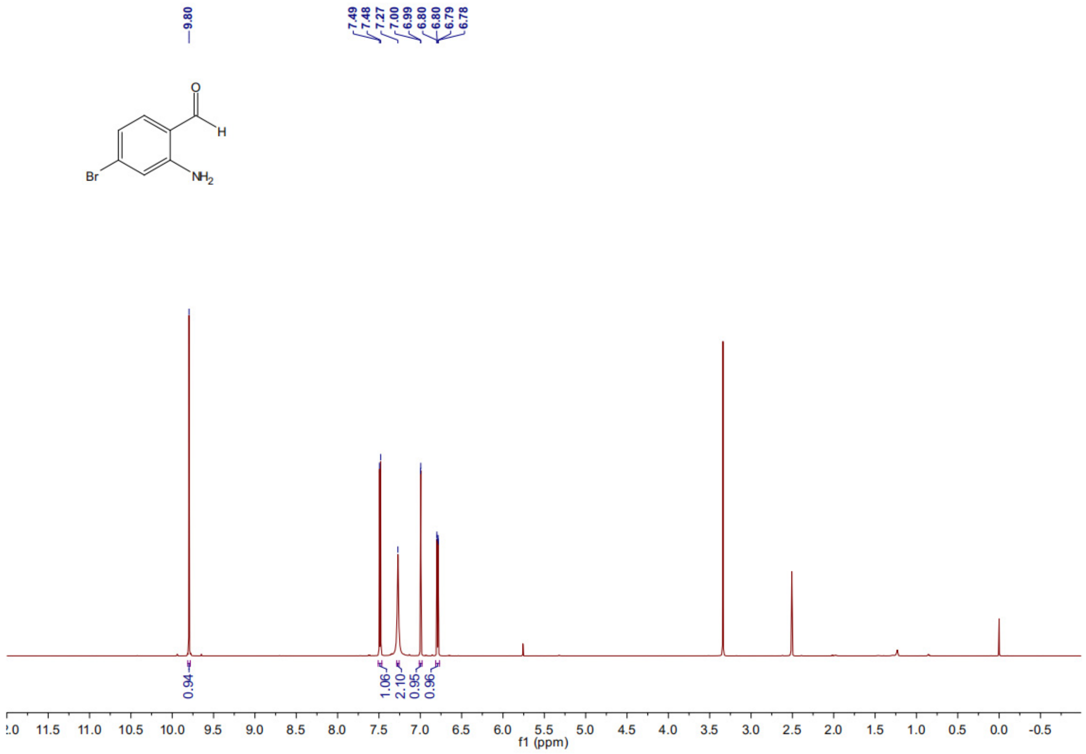

^{1}H-NMR spectrum of intermediate **M4**.

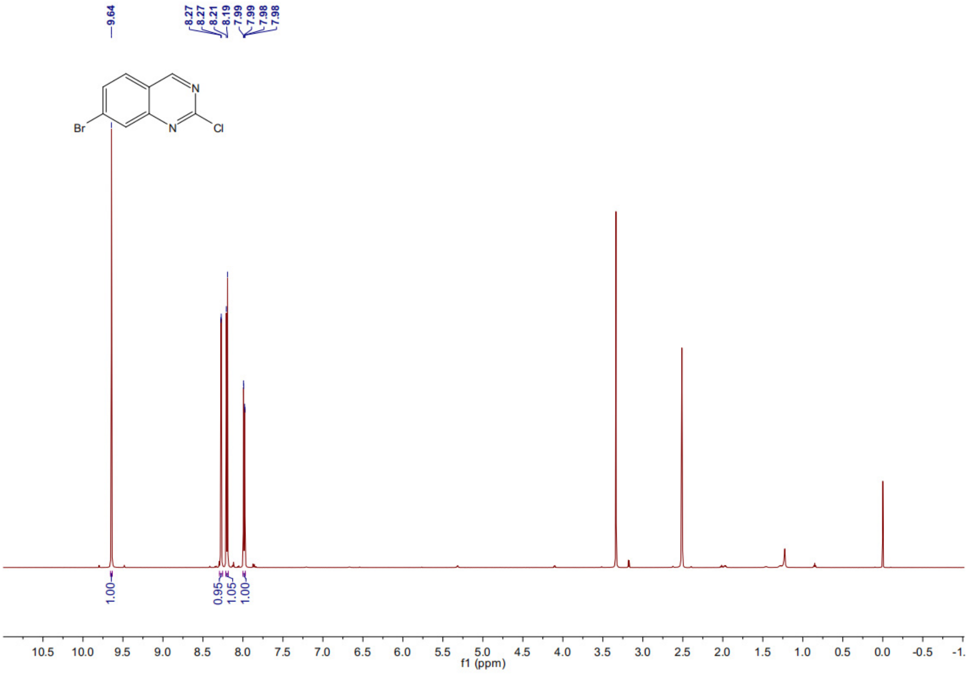

^{1}H-NMR and HRMS spectra of intermediate **M5**.

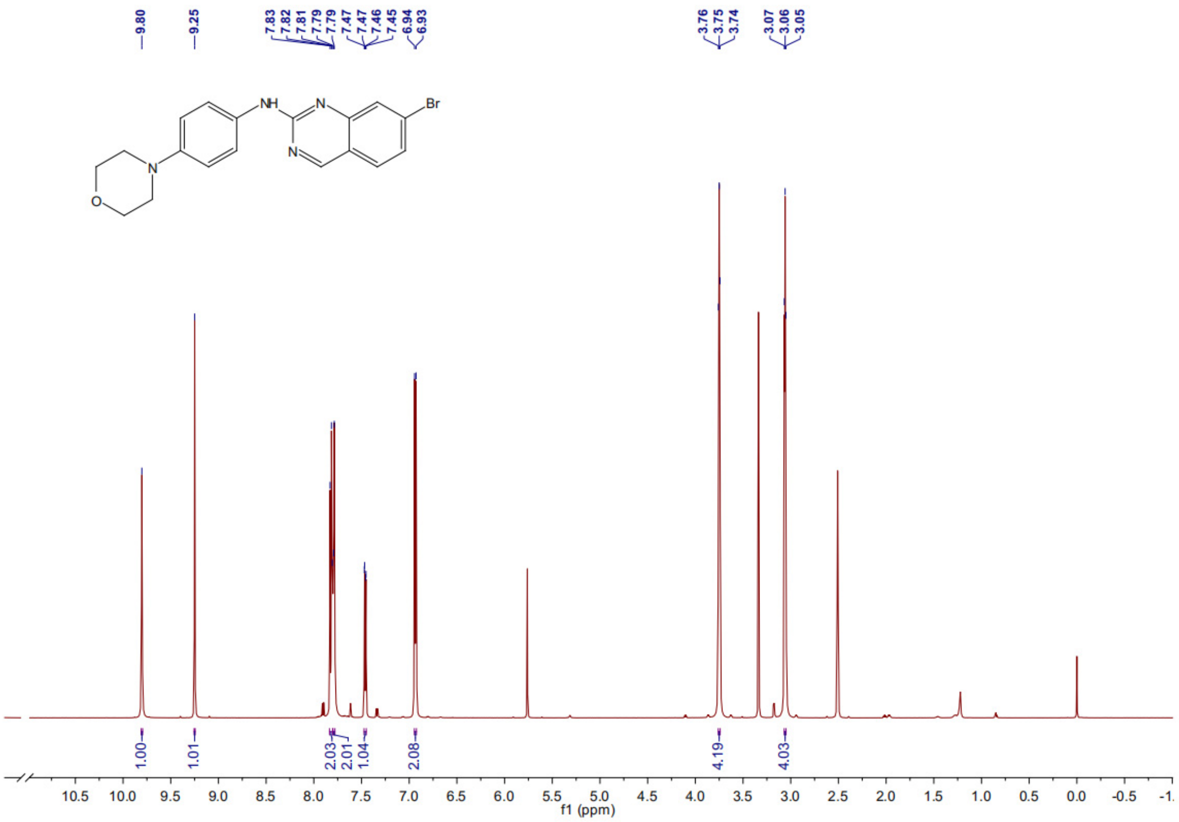

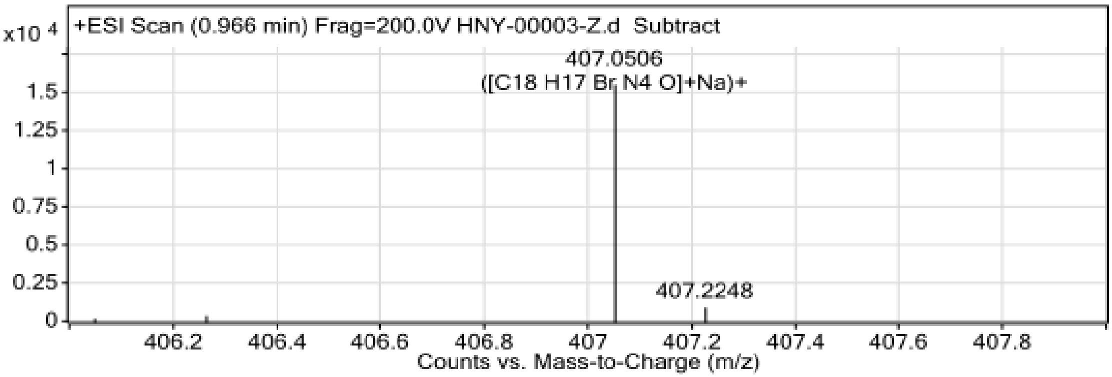

^{1}H-NMR, ^{13}C-NMR, HRMS and HPLC spectra of compound **H1**.

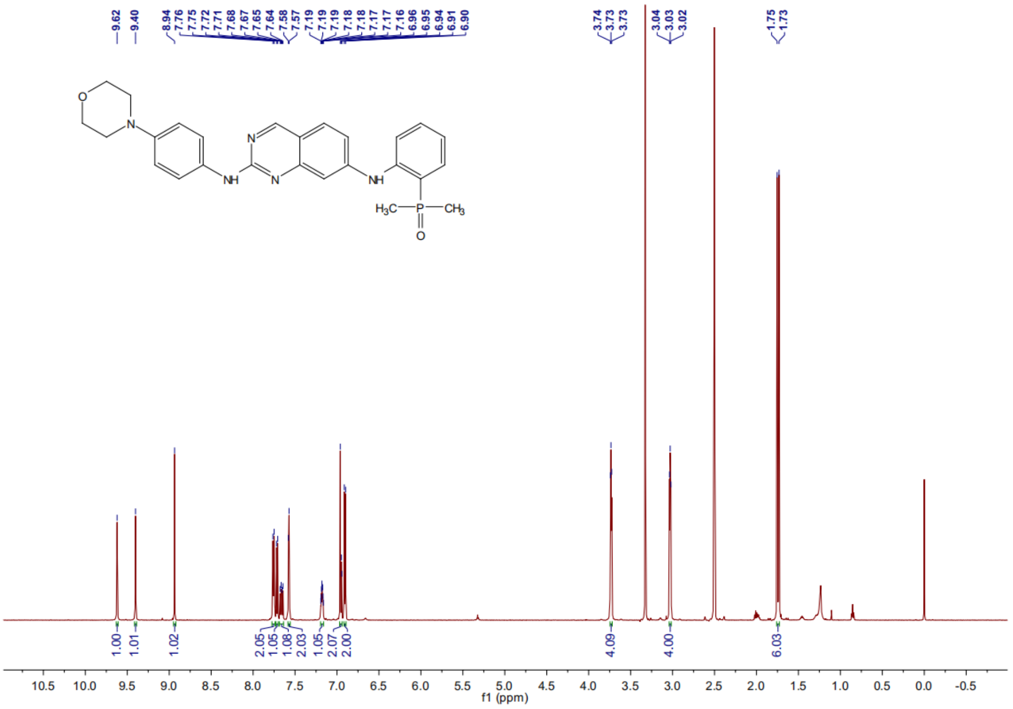

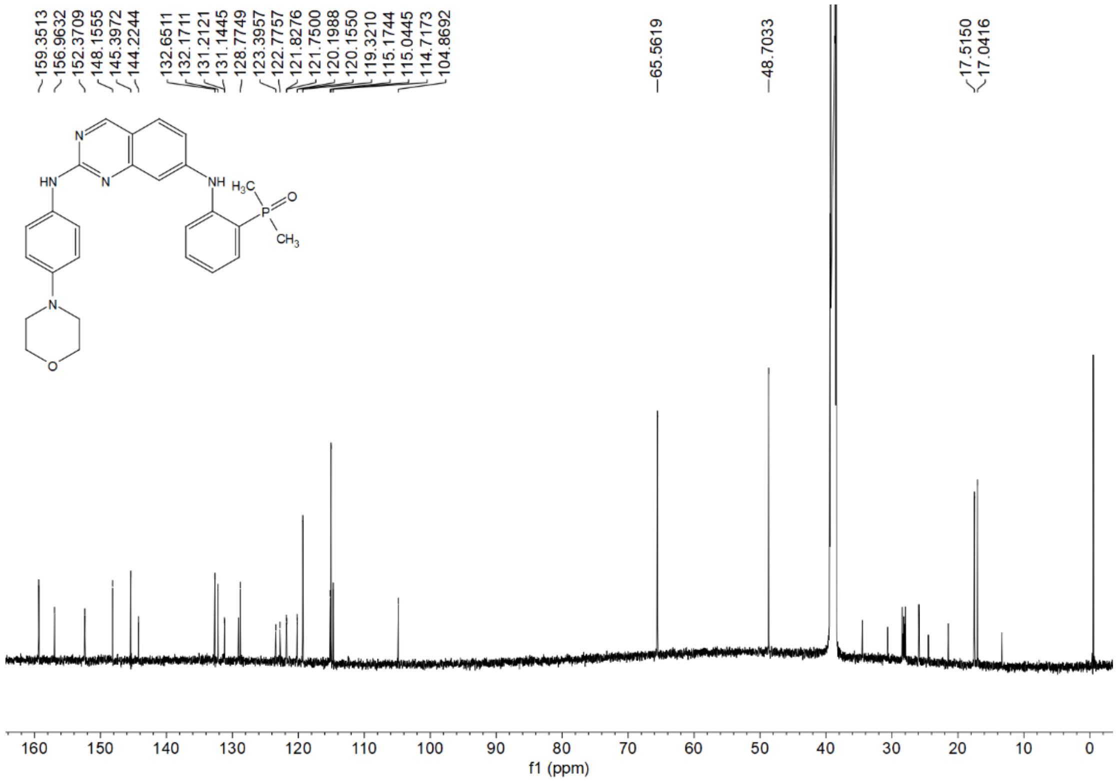

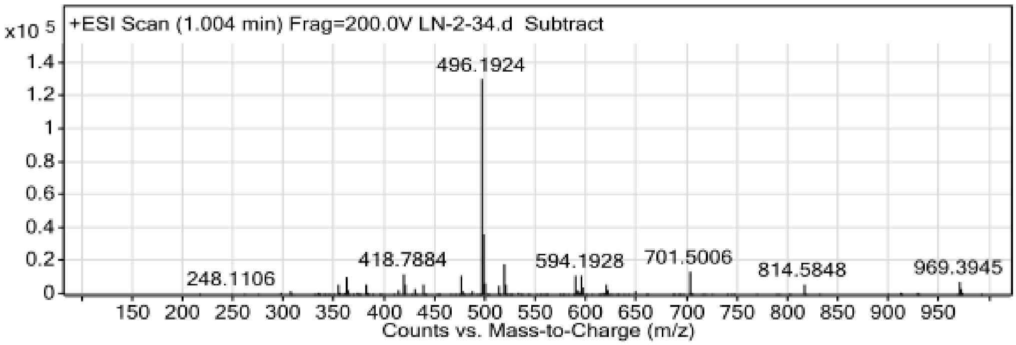

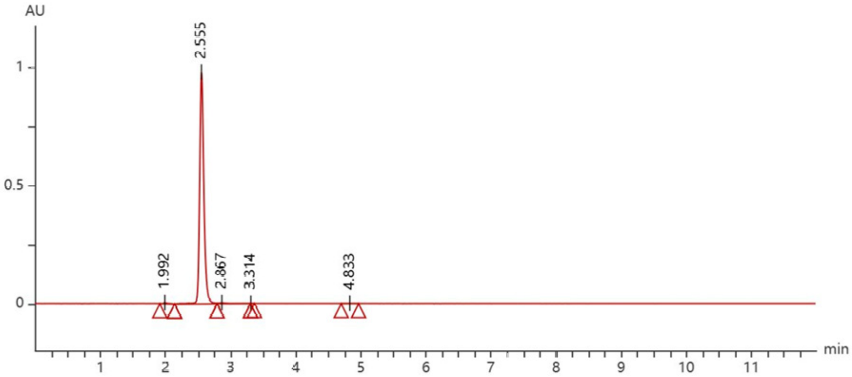

Object List

#	RT (min)	Area (µV*s)	% Area	Height (µV)	% Height
1	1.992	22235.200	0.496	3903.942	0.394
2	2.555	4414472.020	98.375	981811.395	99.147
3	2.867	45094.267	1.005	3623.386	0.366
4	3.314	609.504	0.014	243.331	0.025
5	4.833	4980.267	0.111	680.750	0.069

^{1}H-NMR, ^{13}C-NMR, HRMS and HPLC spectra of compound **H2**.

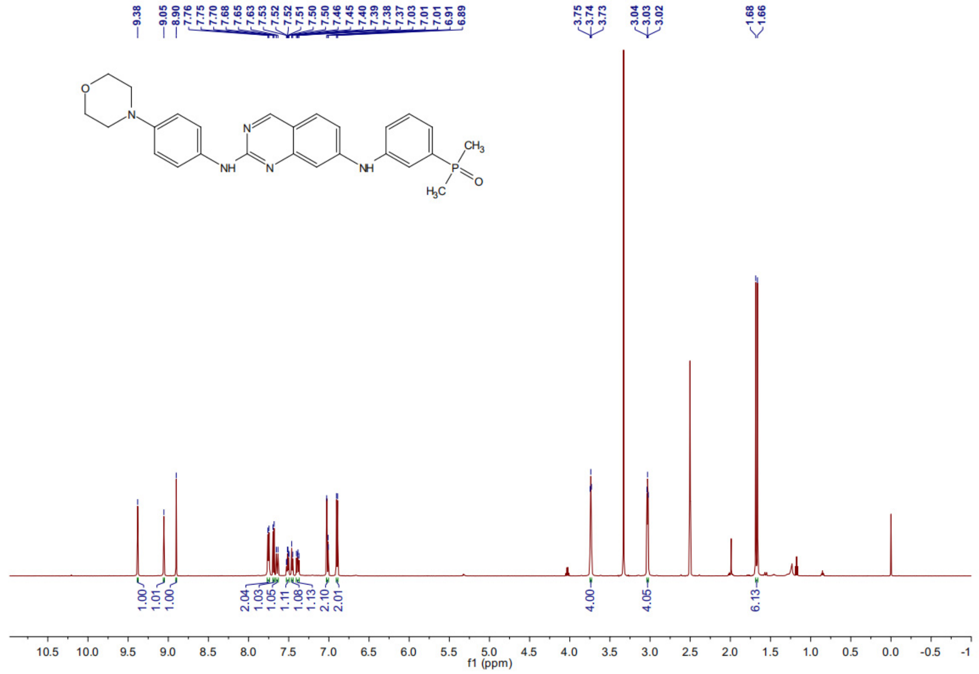

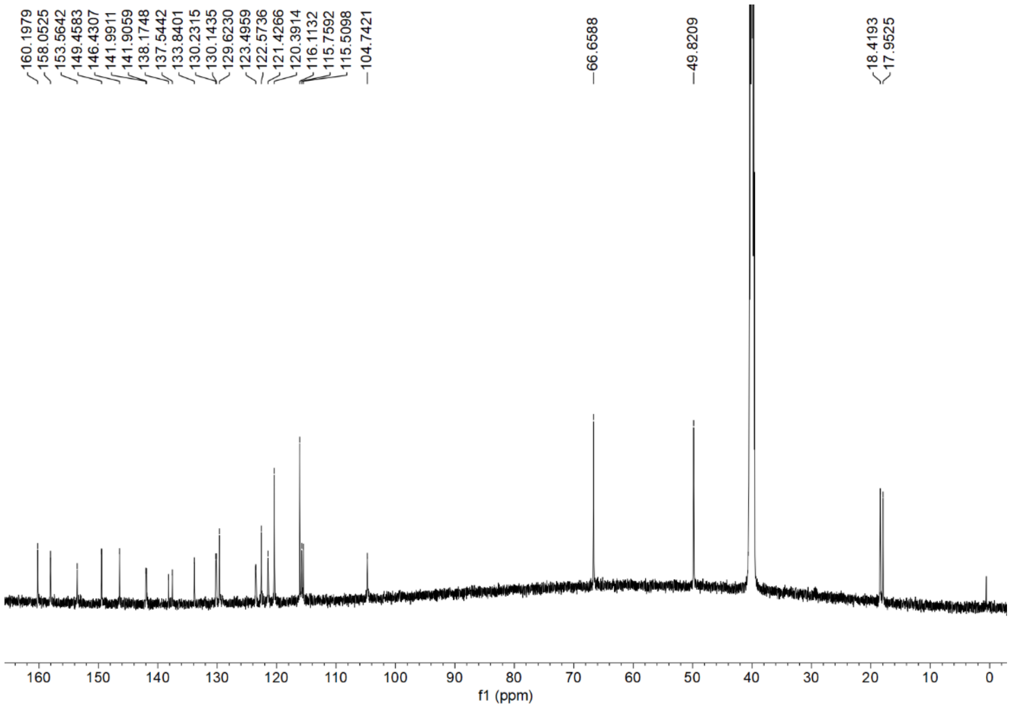

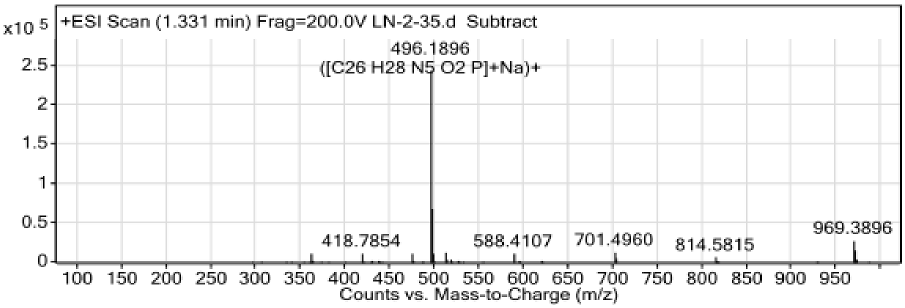

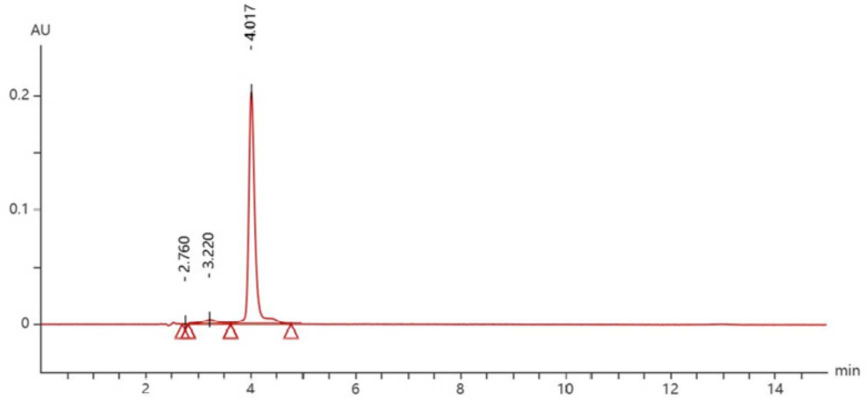

#	RT (min)	Area(μV*s)	High(μV)	Area(%)	High(%)
1	2.760	2203.650	499.688	0.126	0.241
2	3.220	83924.147	3688.130	4.789	1.778
3	4.017	1666413.462	203287.418	95.086	97.982

^{1}H-NMR, ^{13}C-NMR, HRMS and HPLC spectra of compound **H3**.

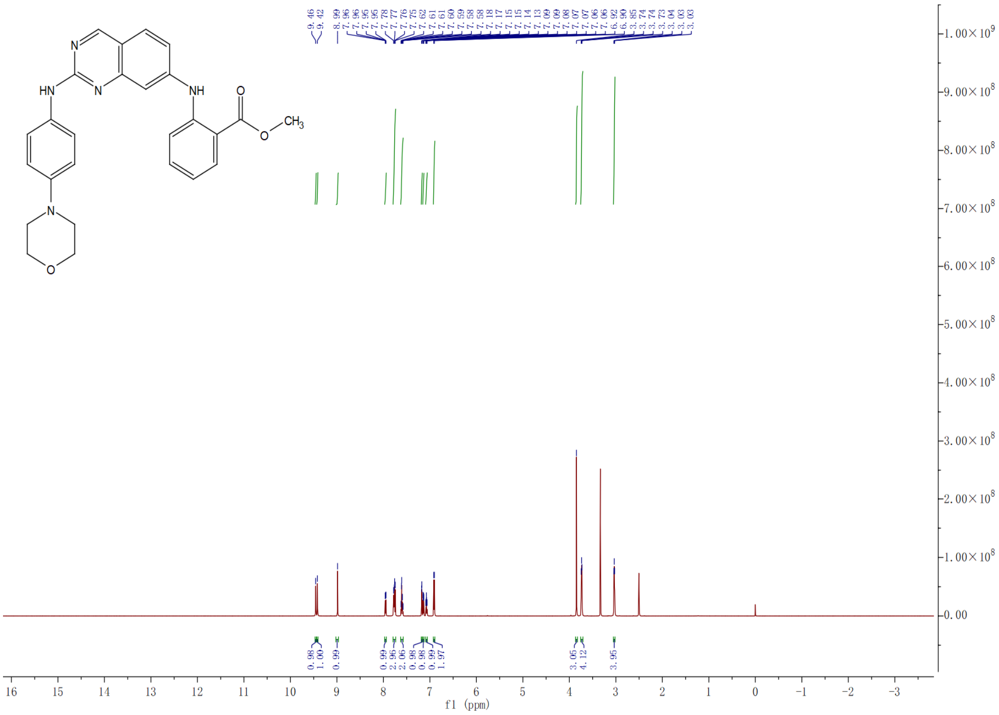

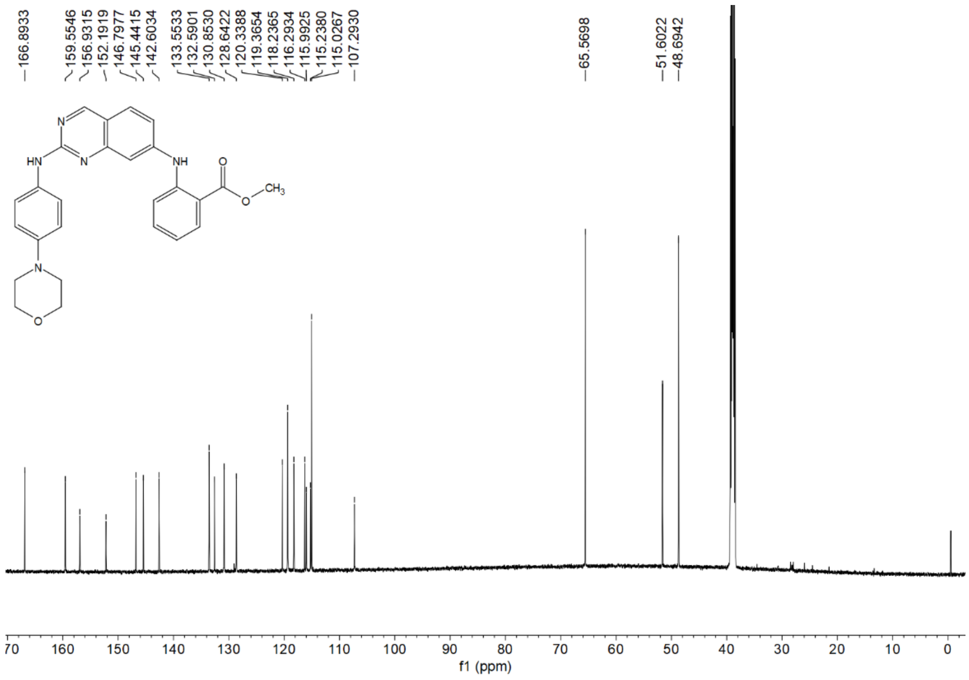

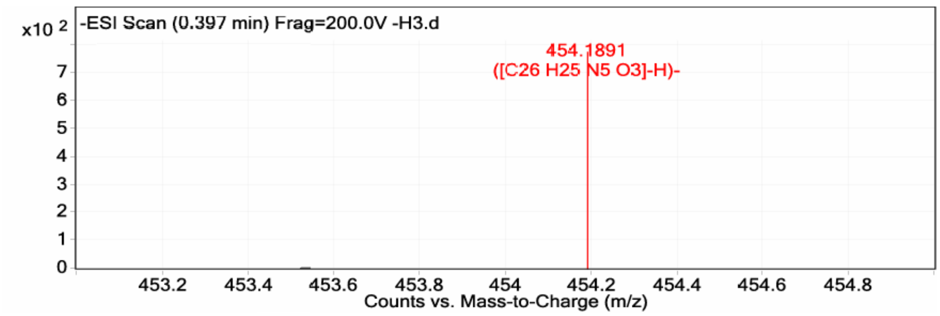

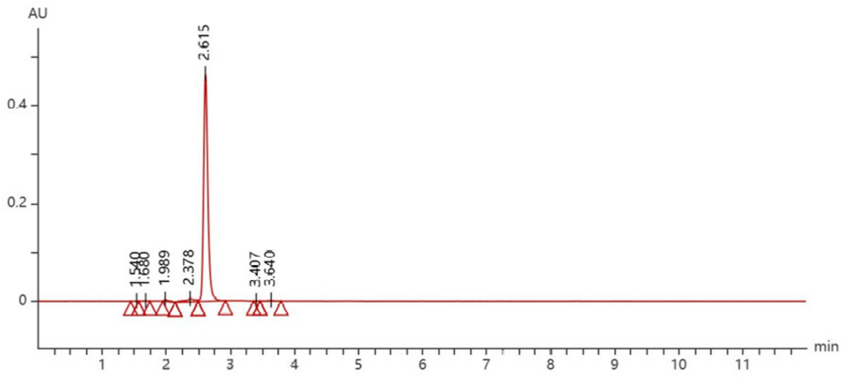

Object List

#	RT (min)	Area (µV*s)	% Area	Height (µV)	% Height
1	1.540	1159.179	0.052	254.856	0.054
2	1.680	2182.938	0.099	536.639	0.113
3	1.989	20024.473	0.905	3565.516	0.749
4	2.378	69159.194	3.124	5948.601	1.249
5	2.615	2112489.606	95.427	464604.012	97.535
6	3.407	339.392	0.015	131.700	0.028
7	3.640	8376.008	0.378	1303.592	0.274

^{1}H-NMR, ^{13}C-NMR, HRMS and HPLC spectra of compound **H4**.

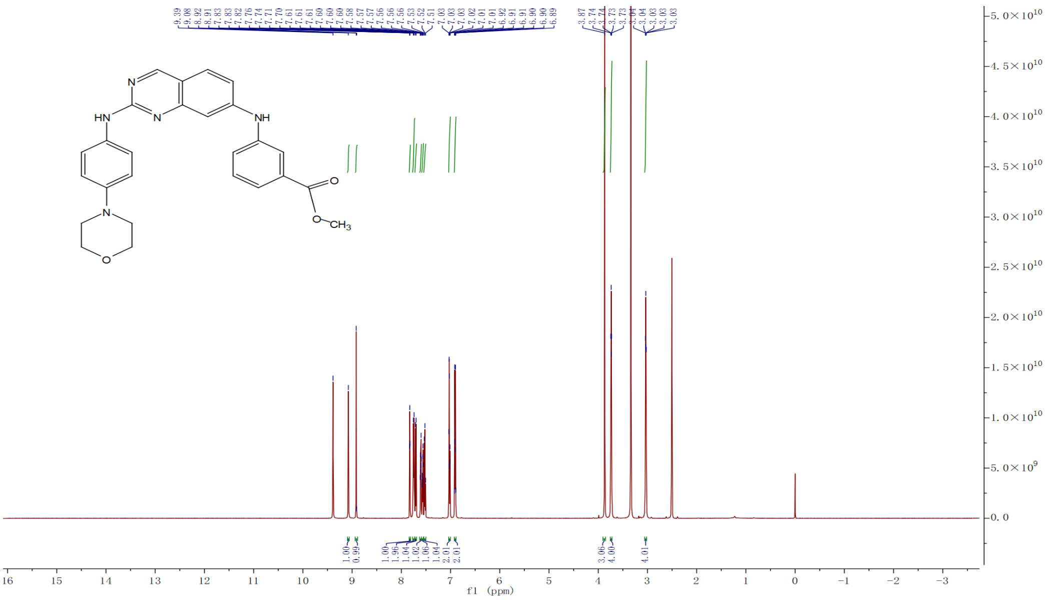

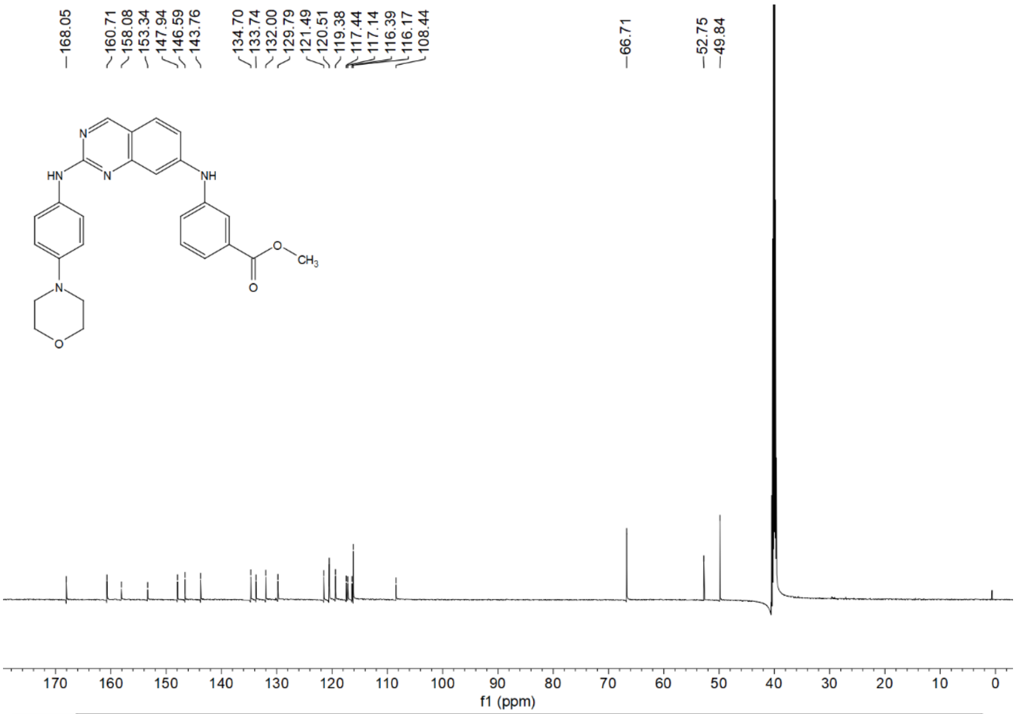

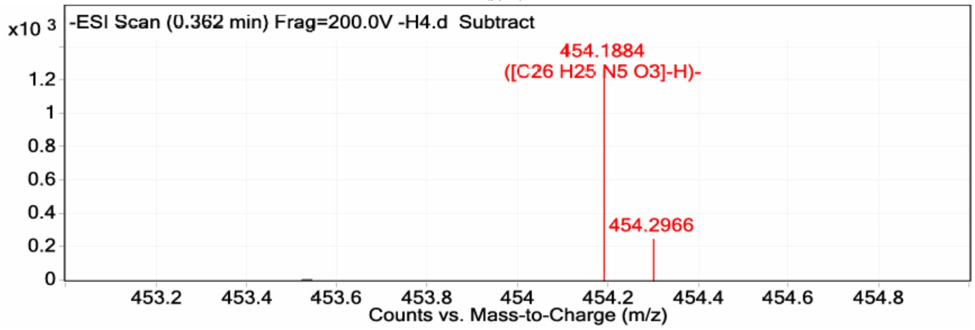

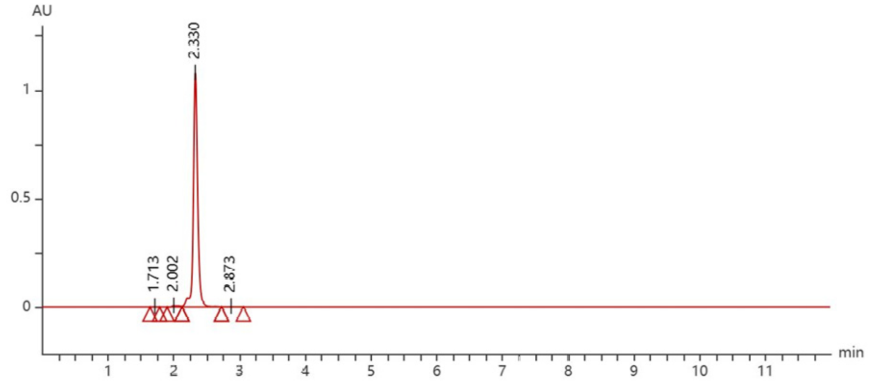

Object List

#	RT (min)	Area (µV*s)	% Area	Height (µV)	% Height
1	1.713	1792.600	0.036	586.000	0.054
2	2.002	42019.749	0.851	4897.891	0.449
3	2.330	4883020.793	98.900	1084001.228	99.416
4	2.873	10481.724	0.212	882.397	0.081

^{1}H-NMR, ^{13}C-NMR, HRMS and HPLC spectra of compound **H5**.

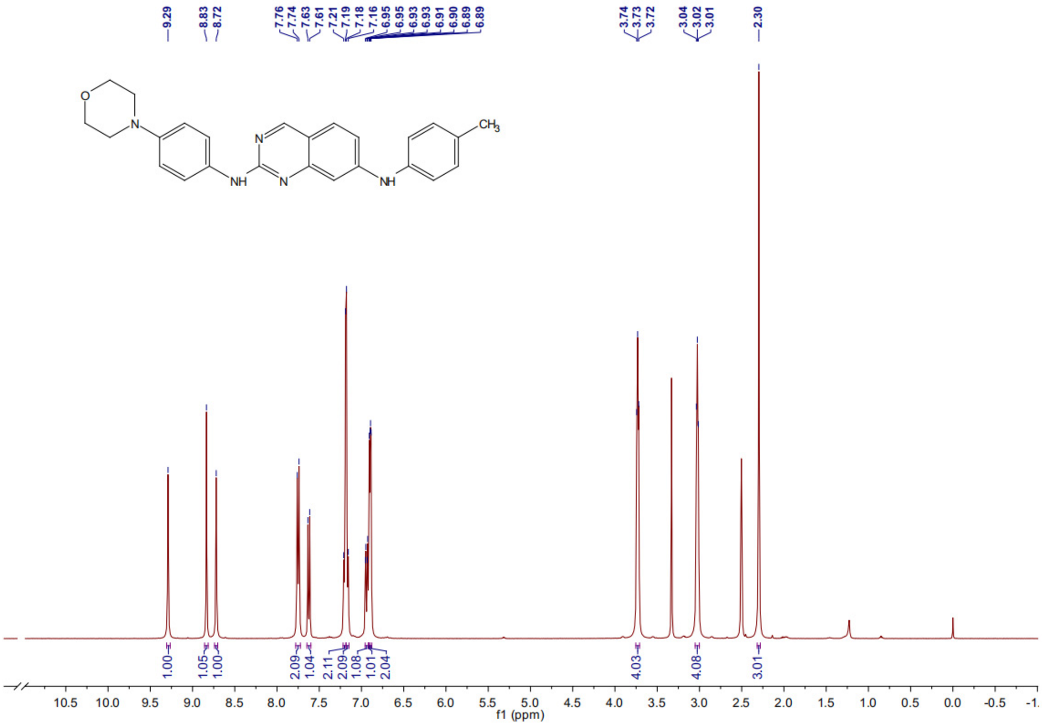

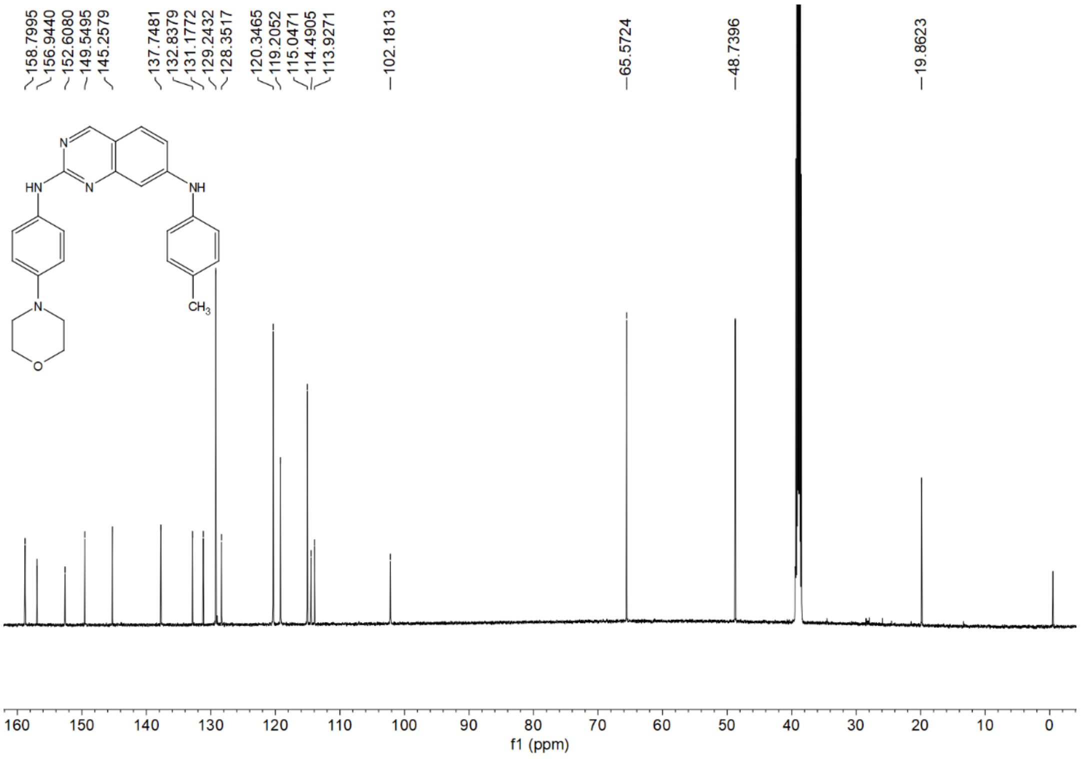

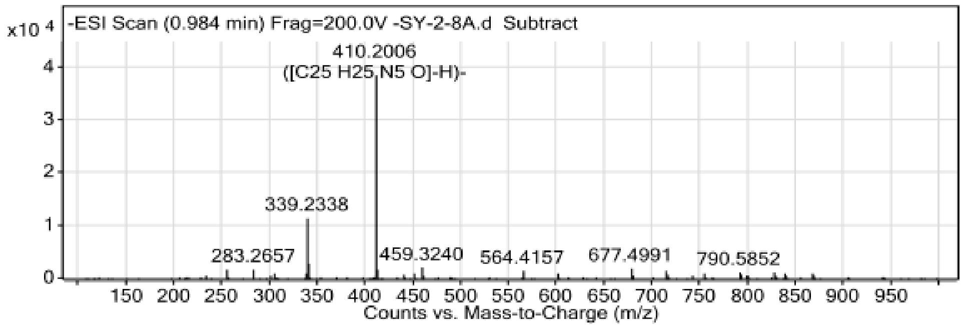

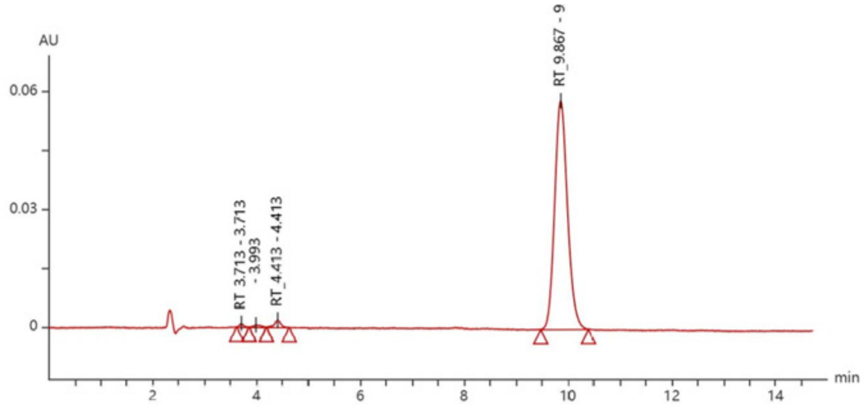

#	RT (min)	Area(μV*s)	High(μV)	Area(%)	High(%)
1	2.760	-	-	-	-
2	3.220	-	-	-	-
3	3.713	5944.812	892.694	0.576	1.449
4	3.993	7278.470	647.776	0.705	1.051
5	4.413	17037.018	1897.025	1.650	3.078
6	9.867	1002598.533	58187.185	97.070	94.422

^{1}H-NMR, ^{13}C-NMR, HRMS and HPLC spectra of compound **H6**.

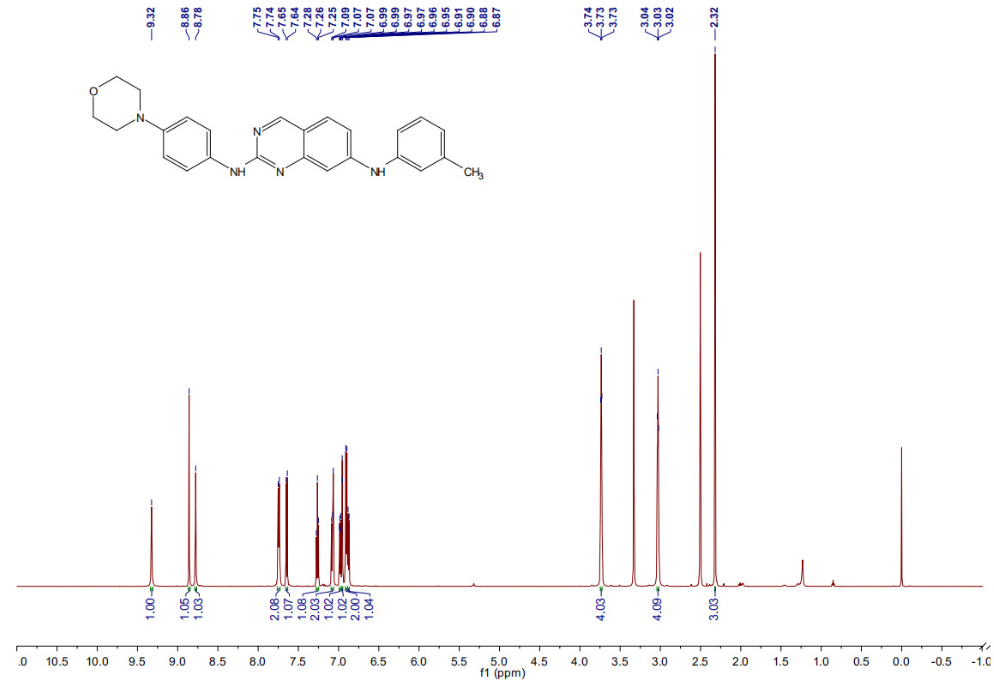

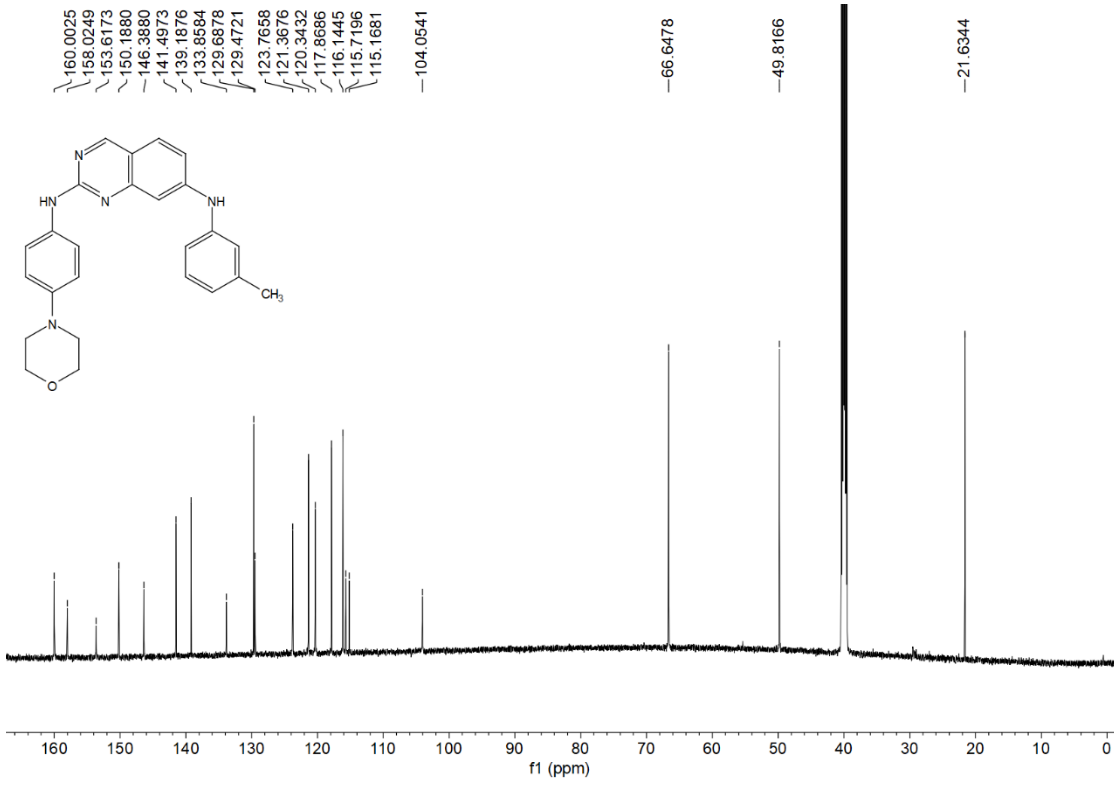

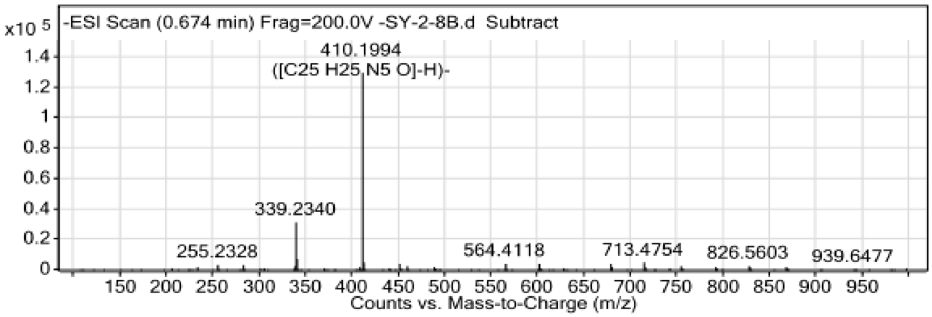

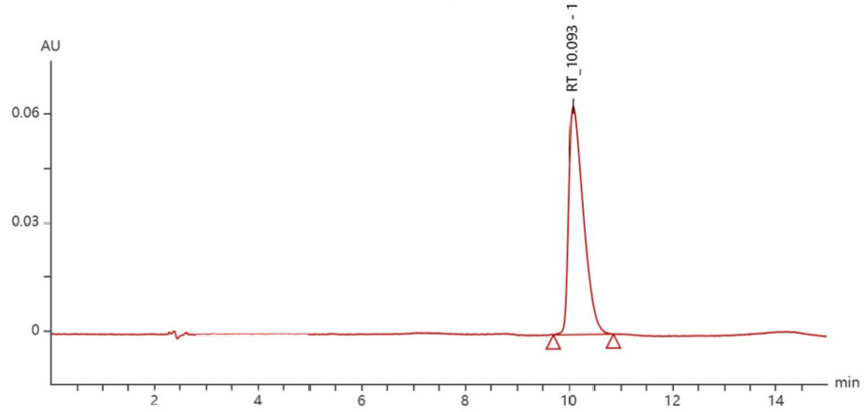

#	RT (min)	Area(μV*s)	High(μV)	Area(%)	High(%)
1	2.760	-	-	-	-
2	3.220	-	-	-	-
3	4.017	-	-	-	-
4	10.093	1239794.133	63047.000	100.000	100.000

^{1}H-NMR, ^{13}C-NMR, HRMS and HPLC spectra of compound **H7**.

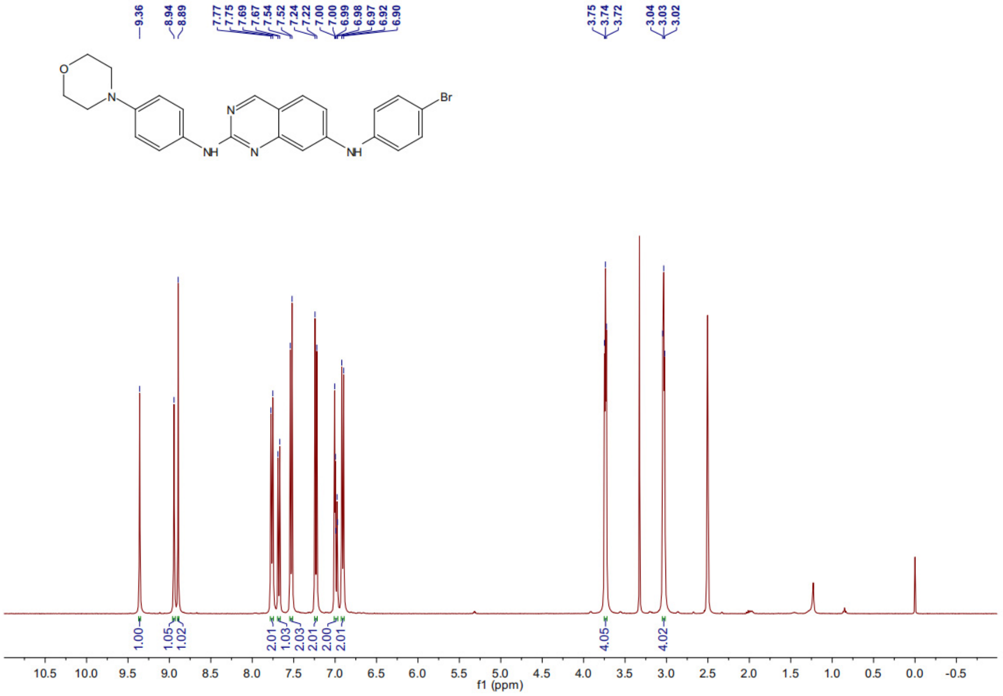

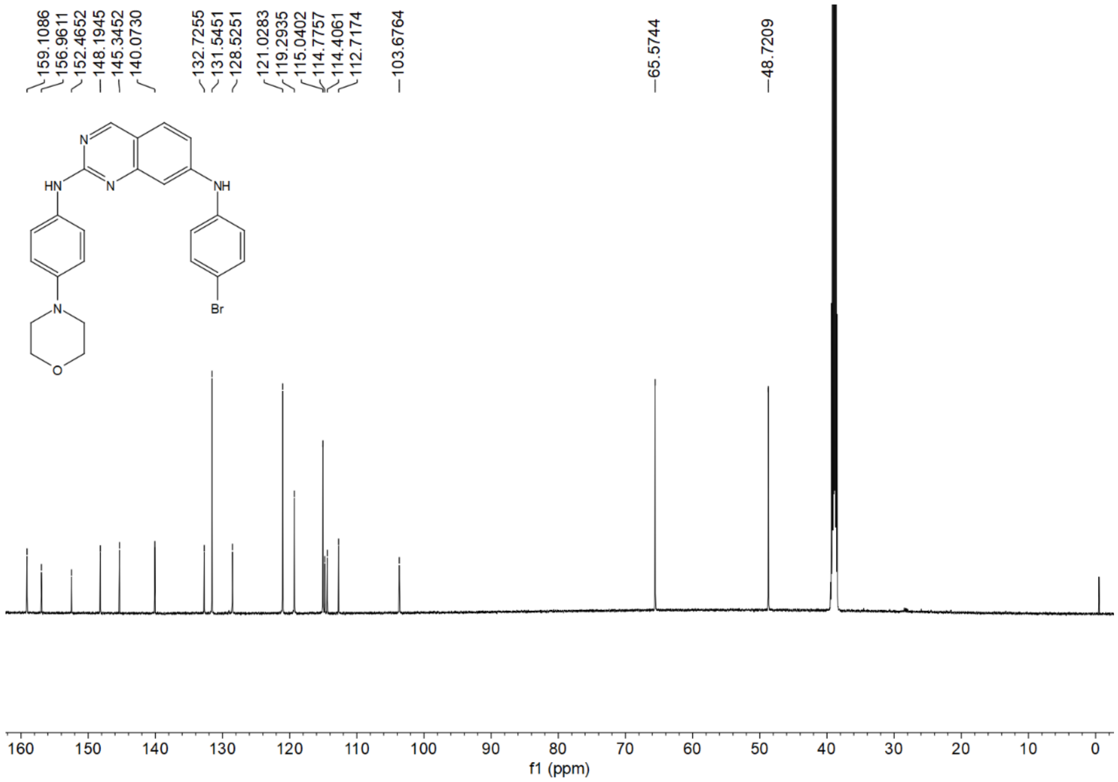

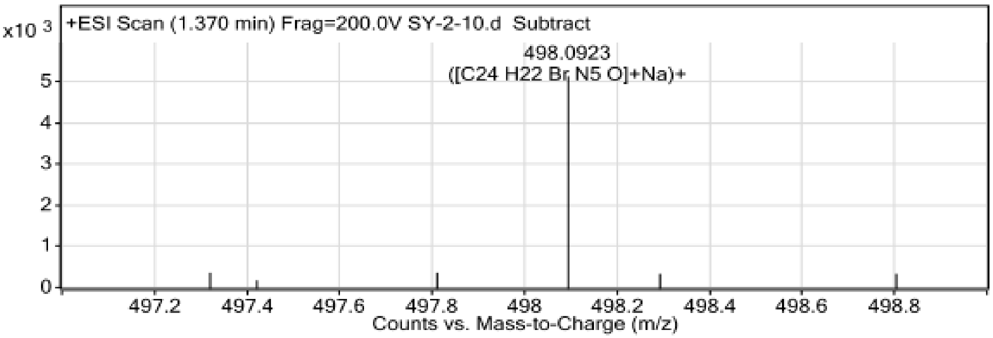

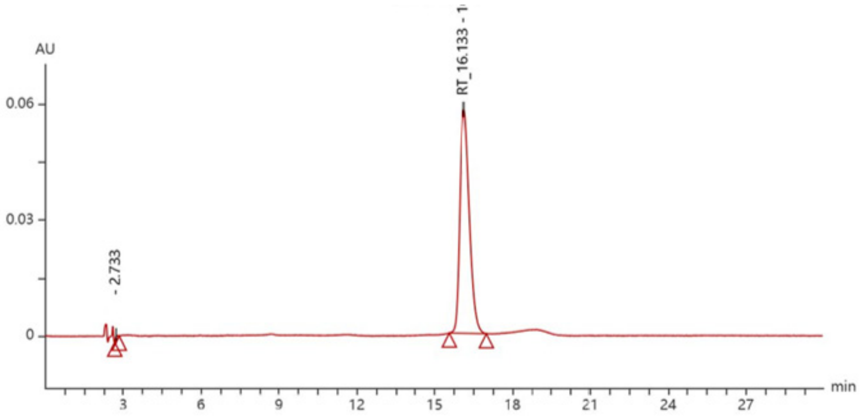

#	RT (min)	Area(μV*s)	High(μV)	Area(%)	High(%)
1	2.733	6548.400	1083.923	0.452	1.835
2	3.220	-	-	-	-
3	4.017	-	-	-	-
4	16.133	1442184.267	57978.528	99.548	98.165

^{1}H-NMR, ^{13}C-NMR, HRMS and HPLC spectra of compound **H8**.

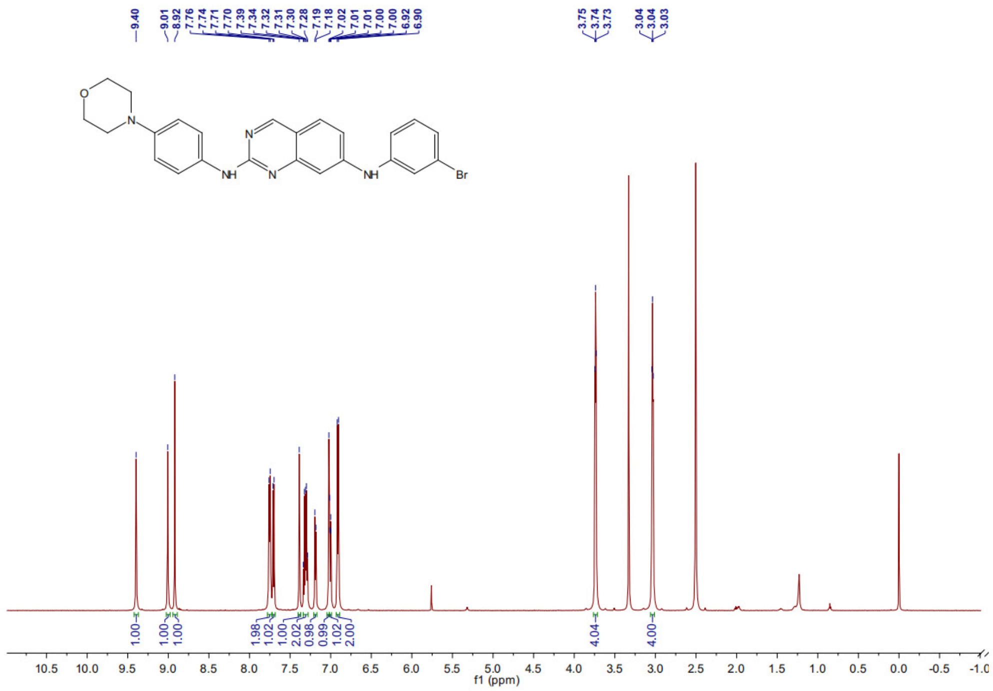

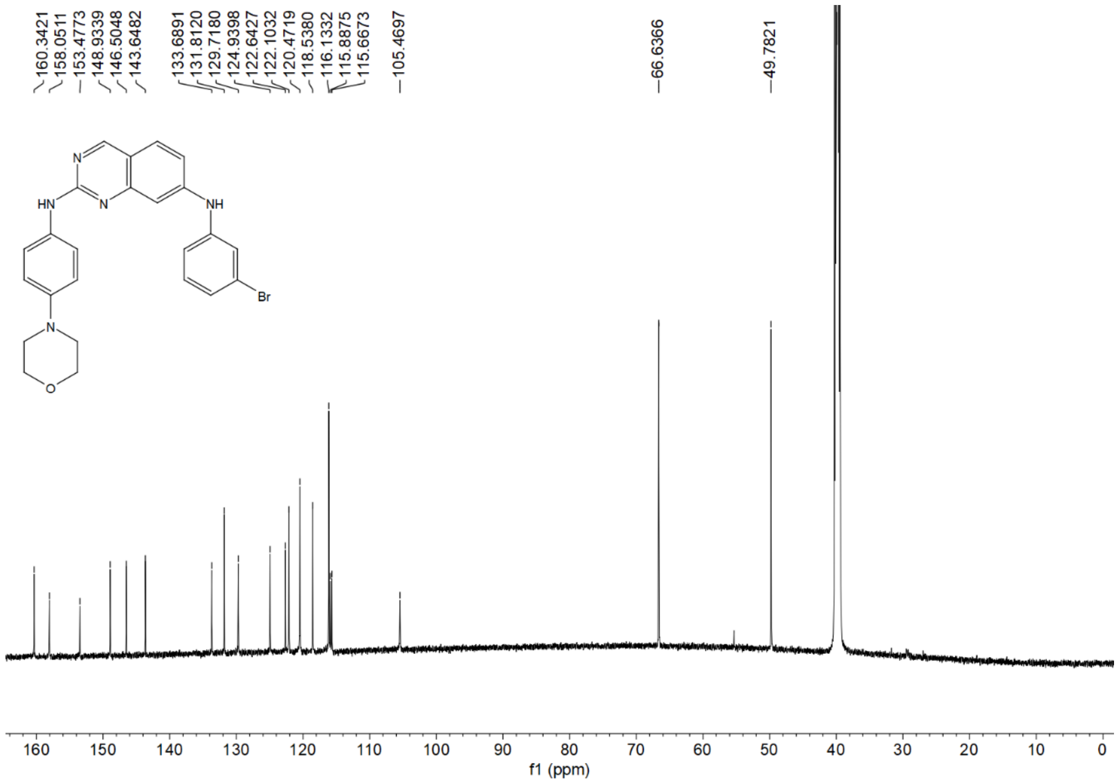

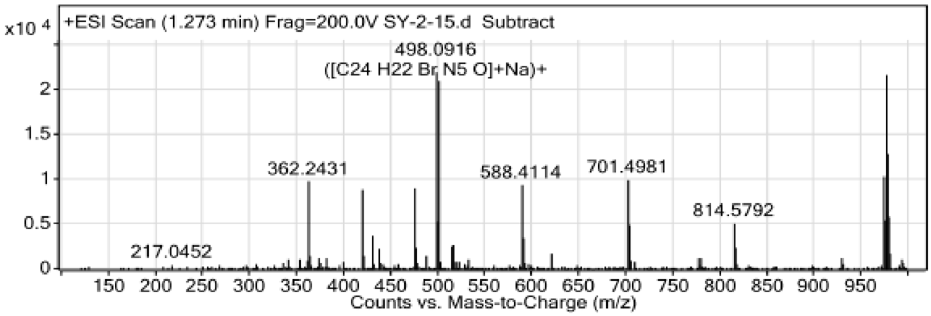

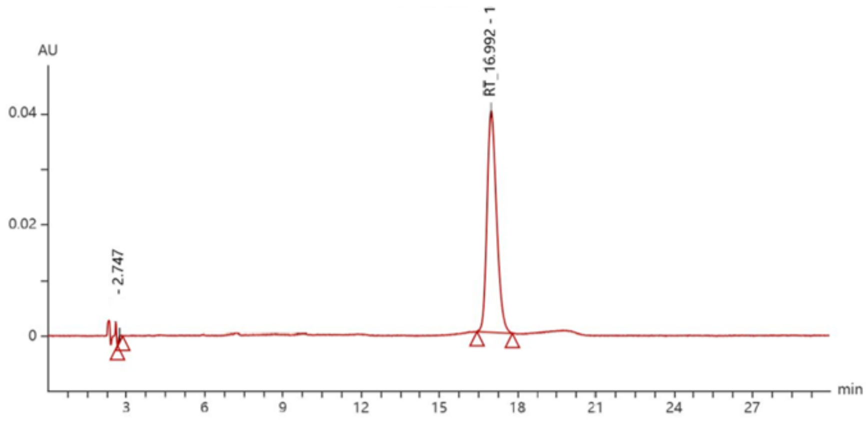

#	RT (min)	Area(μV*s)	High(μV)	Area(%)	High(%)
1	2.747	6975.667	1053.850	0.684	2.581
2	3.220	-	-	-	-
3	4.017	-	-	-	-
4	16.992	1012409.250	39774.492	99.316	97.419

^{1}H-NMR, ^{13}C-NMR, HRMS and HPLC spectra of compound **H9**.

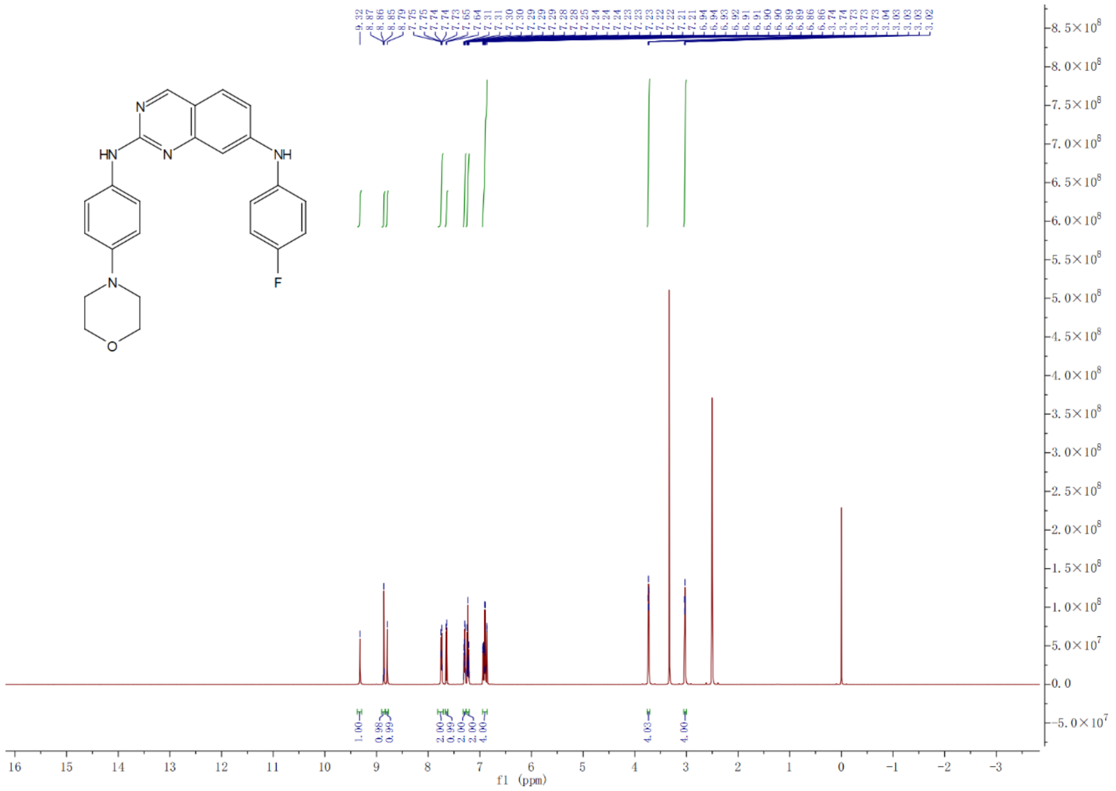

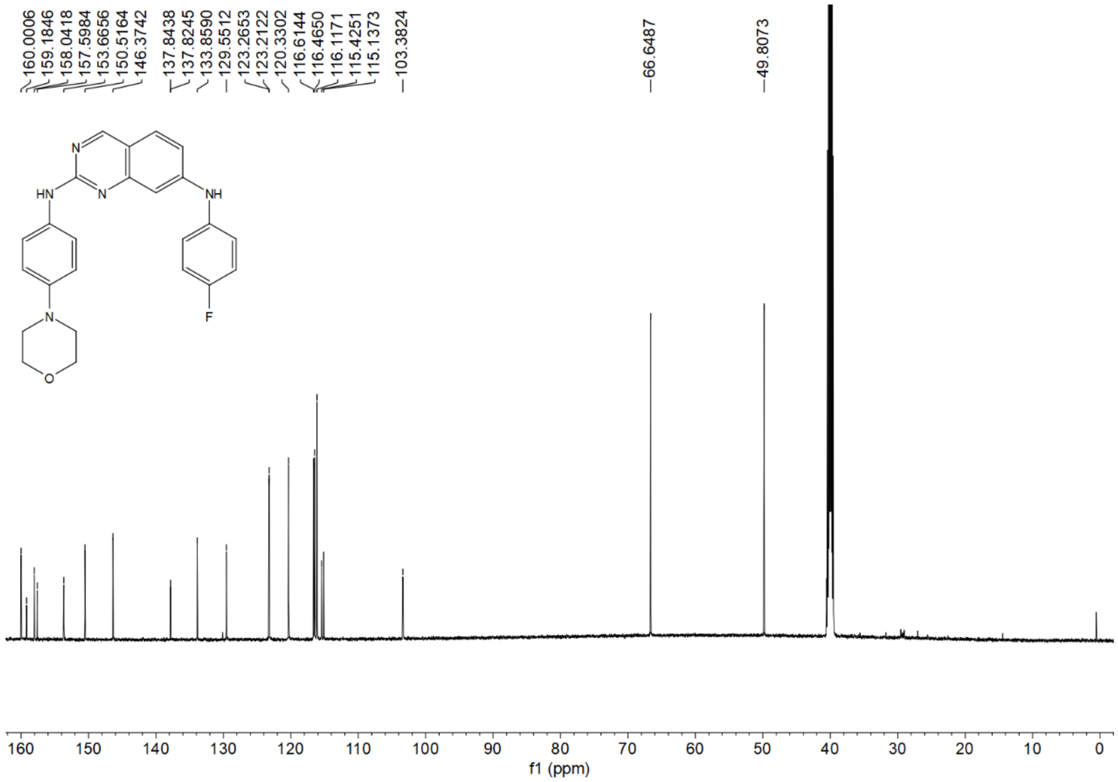

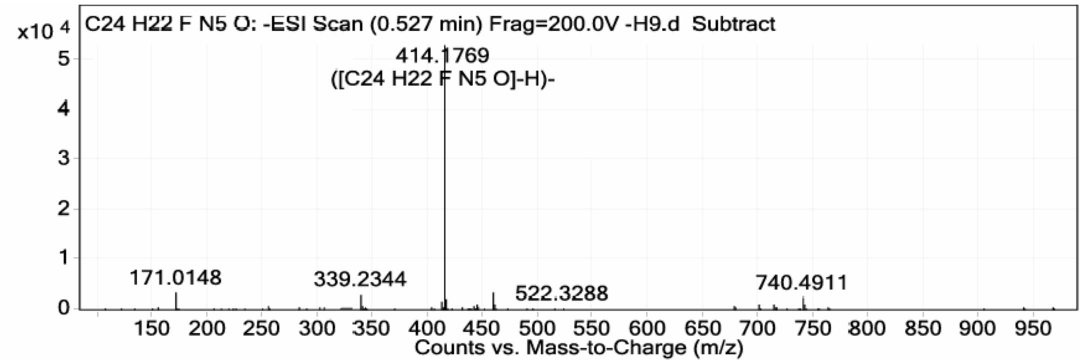

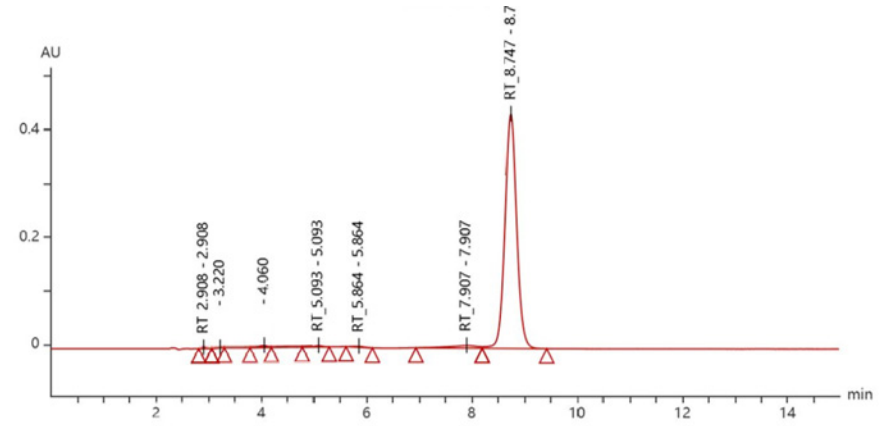

#	RT (min)	Area(μV*s)	High(μV)	Area(%)	High(%)
1	2.760	-	-	-	-
2	2.908	10692.392	1958.149	0.152	0.434
3	3.220	3716.400	635.417	0.053	0.141
4	4.060	22961.583	2726.934	0.325	0.604
5	5.093	31380.433	2711.536	0.445	0.601
6	5.864	28209.183	1756.986	0.400	0.389
7	7.907	196672.138	5014.182	2.787	1.111
8	8.747	6763018.145	436625.483	95.839	96.721

^{1}H-NMR, ^{13}C-NMR, HRMS and HPLC spectra of compound **H10**.

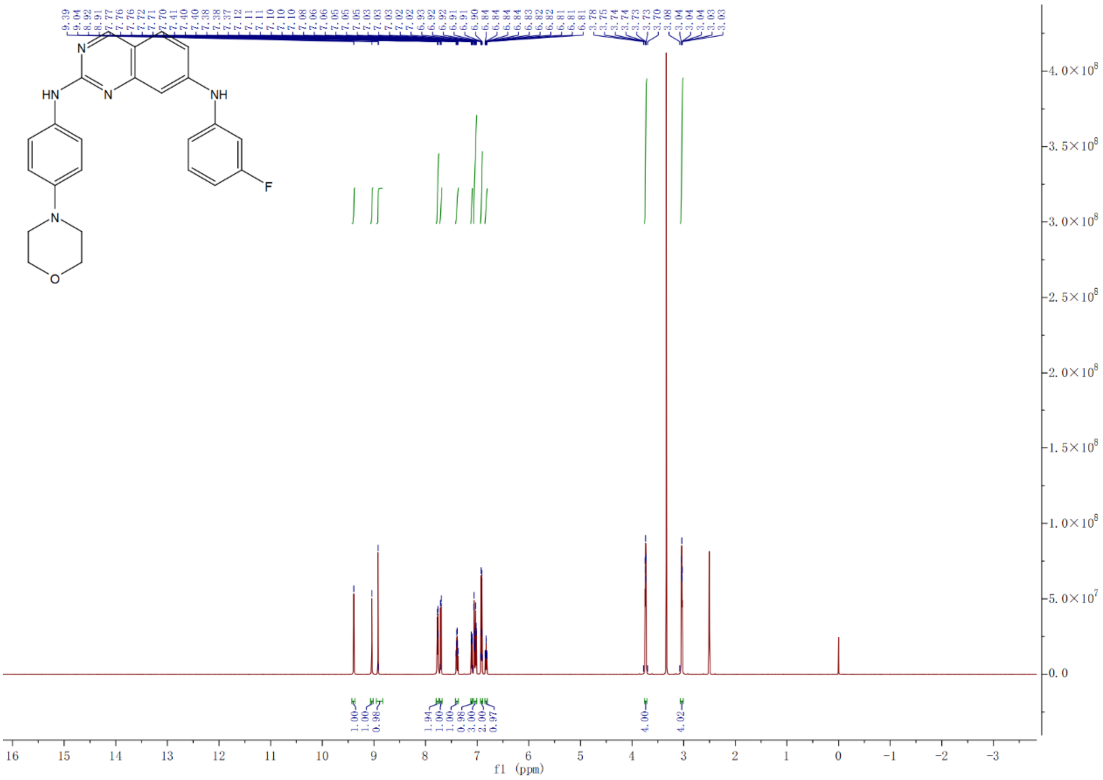

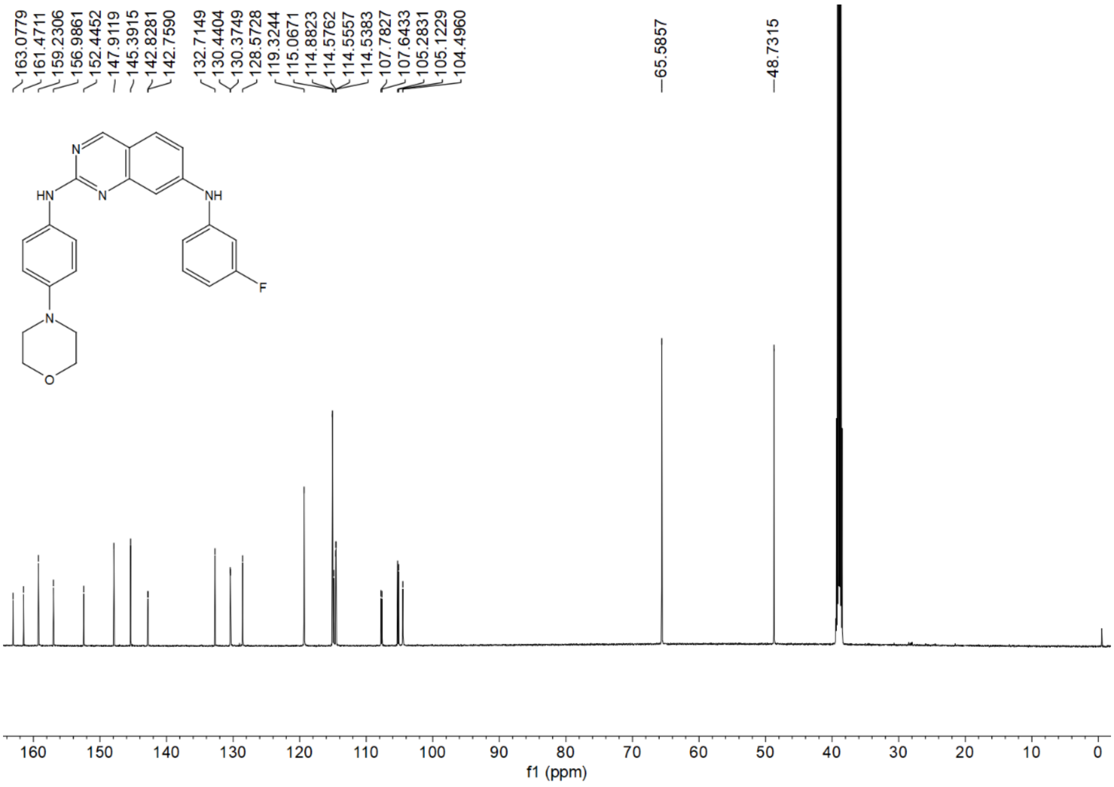

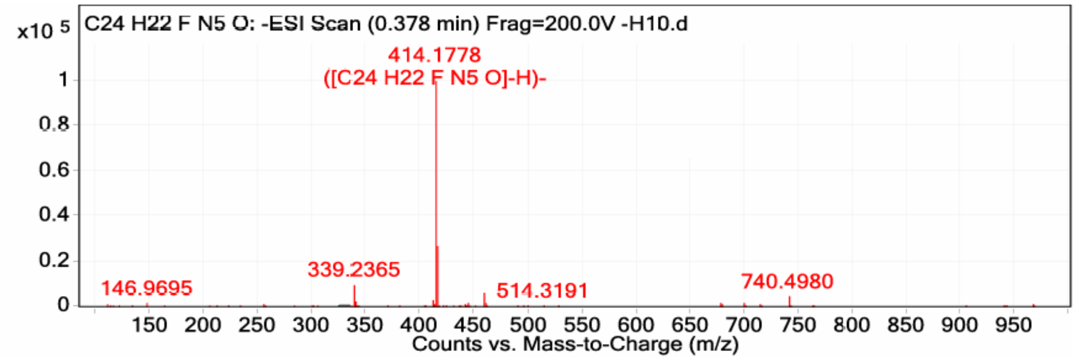

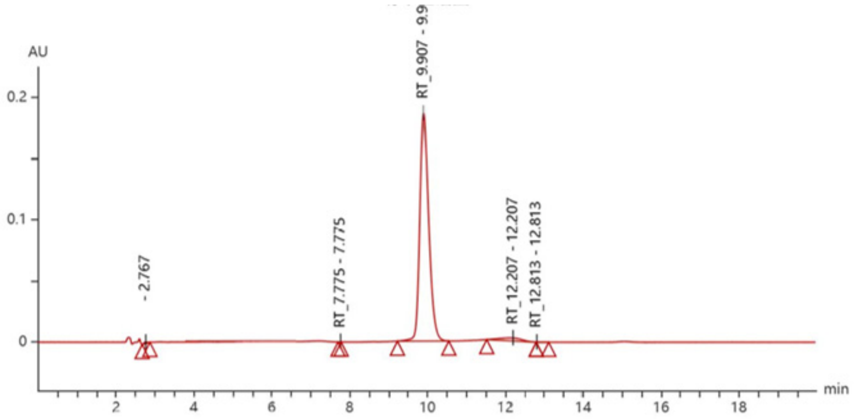

#	RT (min)	Area(μV*s)	High(μV)	Area(%)	High(%)
1	2.767	7817.267	1223.533	0.252	0.642
2	3.220	-	-	-	-
3	4.017	-	-	-	-
4	7.775	305.067	127.151	0.010	0.067
5	9.907	2992166.333	186634.052	96.596	97.909
6	12.207	96051.517	2461.318	3.101	1.291
7	12.813	1252.844	172.979	0.040	0.091

^{1}H-NMR, ^{13}C-NMR, HRMS and HPLC spectra of compound **H11**.

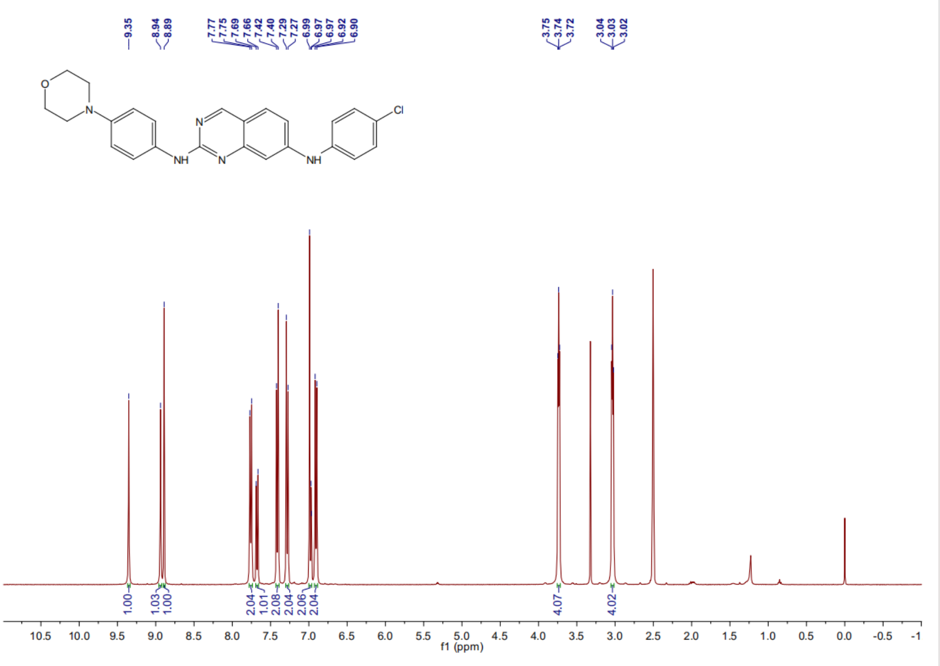

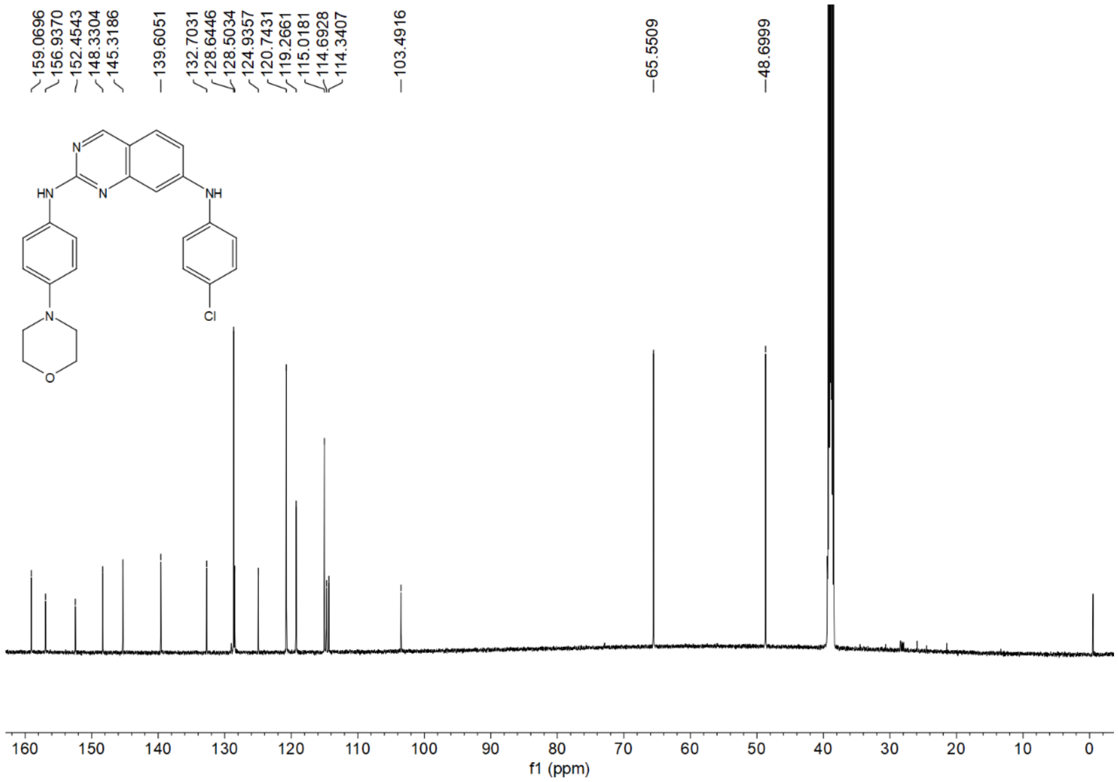

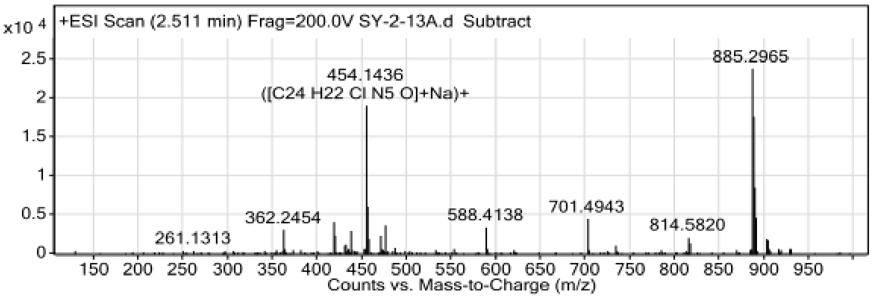

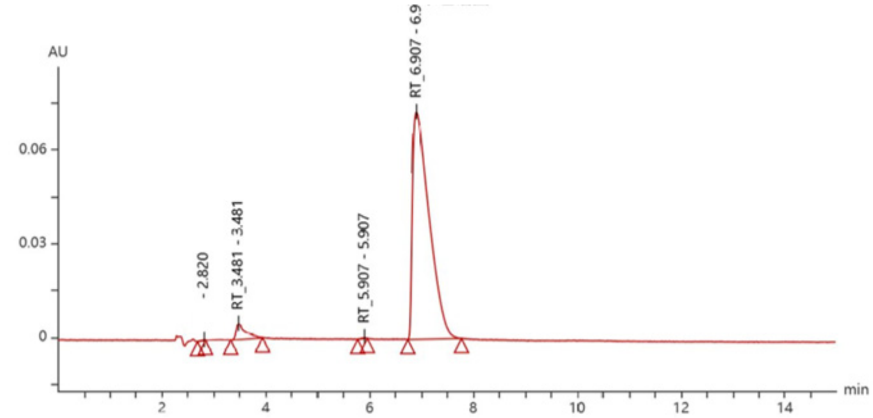

#	RT (min)	Area(μV*s)	High(μV)	Area(%)	High(%)
1	2.820	1536.842	319.783	0.091	0.408
2	3.220	-	-	-	-
3	3.481	65921.933	5048.745	3.905	6.436
4	4.017	-	-	-	-
5	5.907	1960.400	392.643	0.116	0.501
6	6.907	1618894.667	72687.081	95.888	92.656

^{1}H-NMR, ^{13}C-NMR, HRMS and HPLC spectra of compound **H12**.

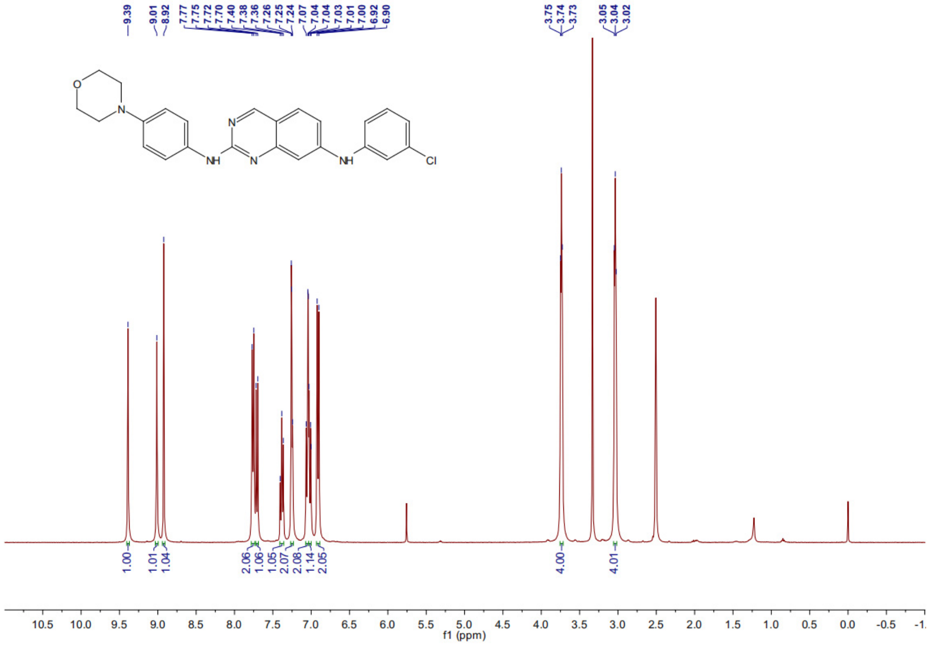

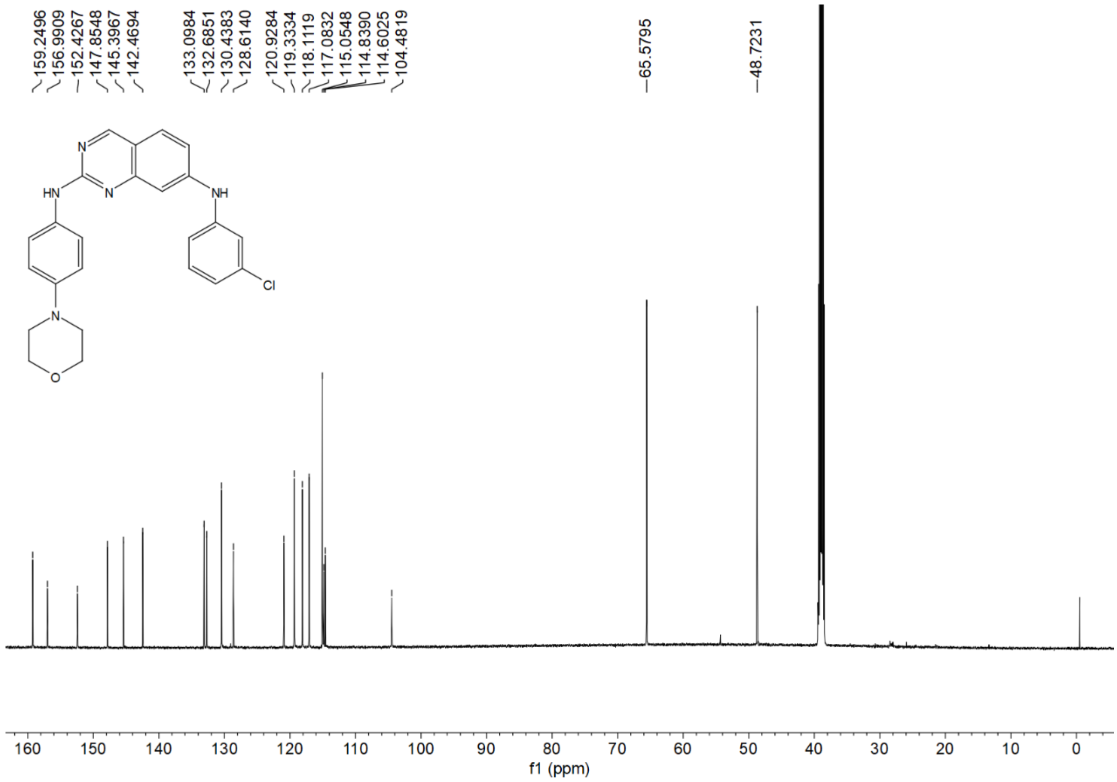

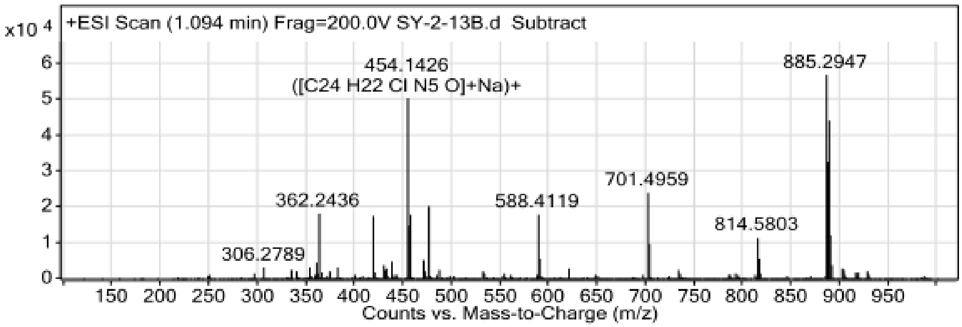

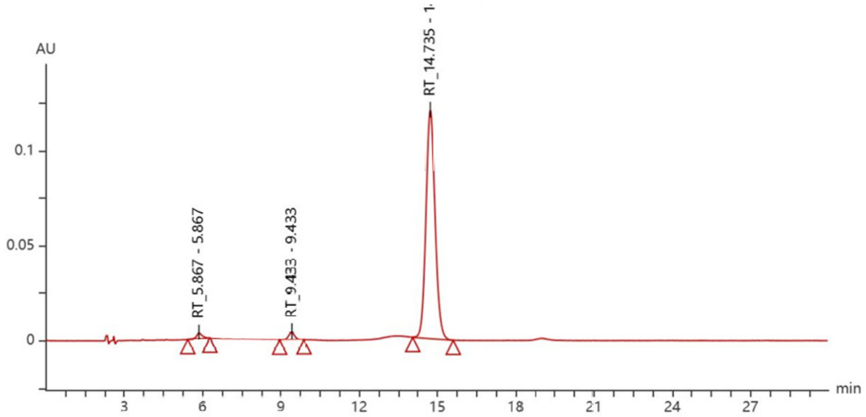

#	RT (min)	Area(µV*s)	High(µV)	Area(%)	High(%)
1	5.867	49179.450	3236.201	1.614	2.535
2	9.433	66526.700	4058.463	2.183	3.179
3	14.735	2931450.433	120358.395	96.203	94.286

^{1}H-NMR, ^{13}C-NMR, HRMS and HPLC spectra of compound **H13**.

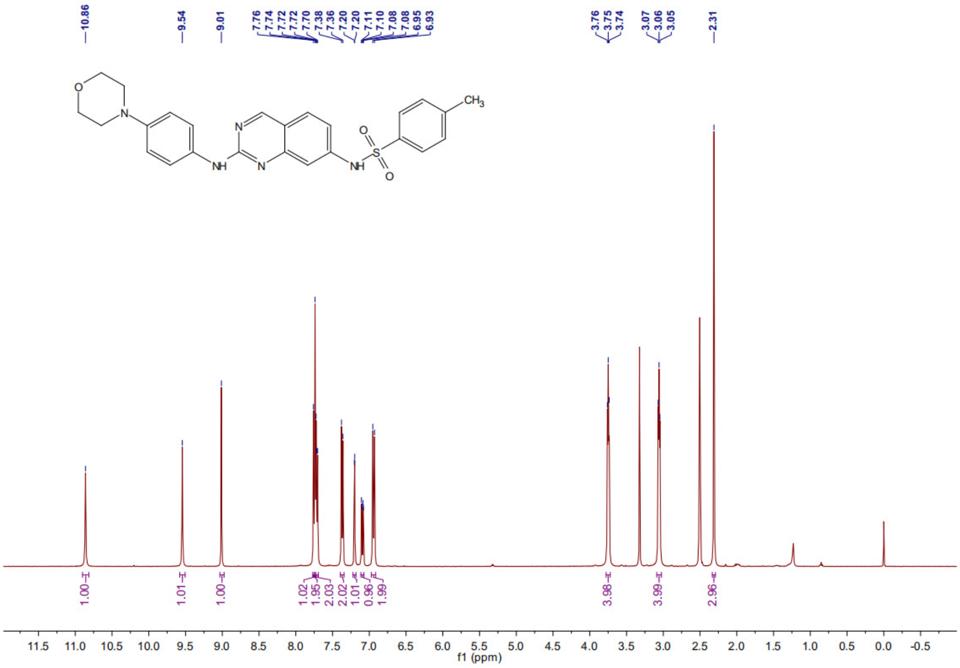

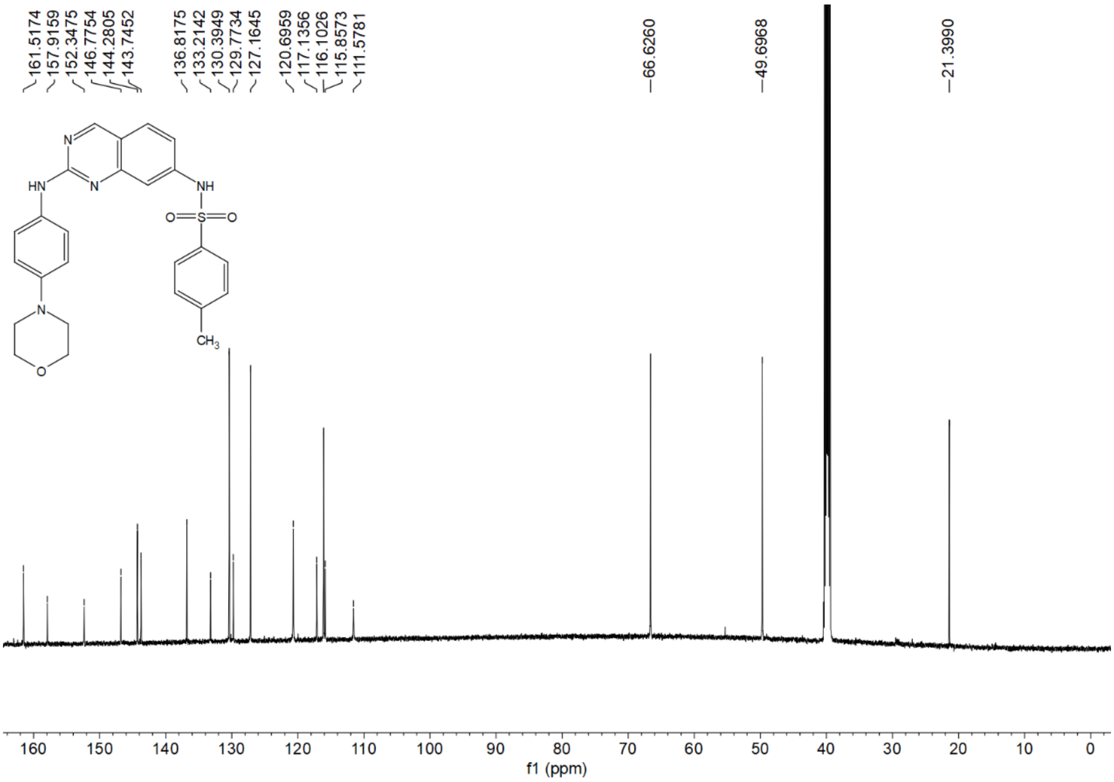

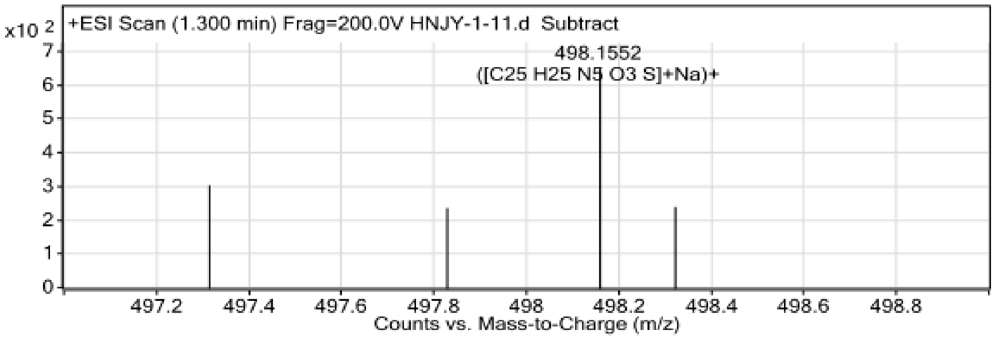

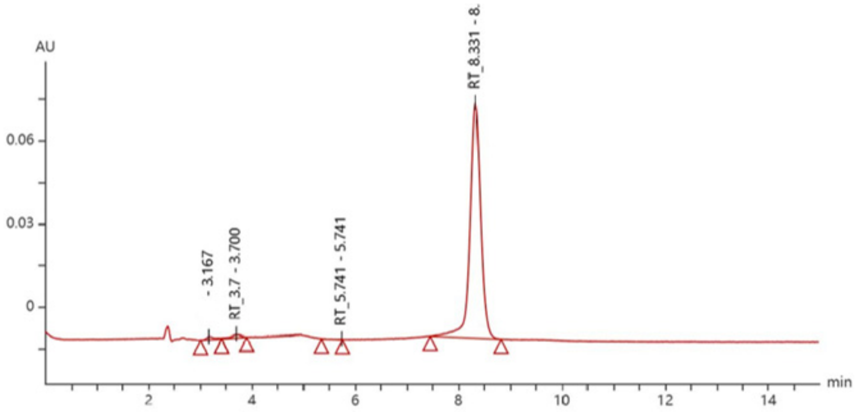

#	RT (min)	Area(μV*s)	High(μV)	Area(%)	High(%)
1	2.760	-	-	-	-
2	3.167	13784.998	1214.187	1.084	1.389
3	3.700	18504.211	1317.642	1.455	1.507
4	4.017	-	-	-	-
5	5.741	1591.430	192.156	0.125	0.220
6	8.331	1237911.200	84688.312	97.336	96.884

^{1}H-NMR, ^{13}C-NMR, HRMS and HPLC spectra of compound **H14**.

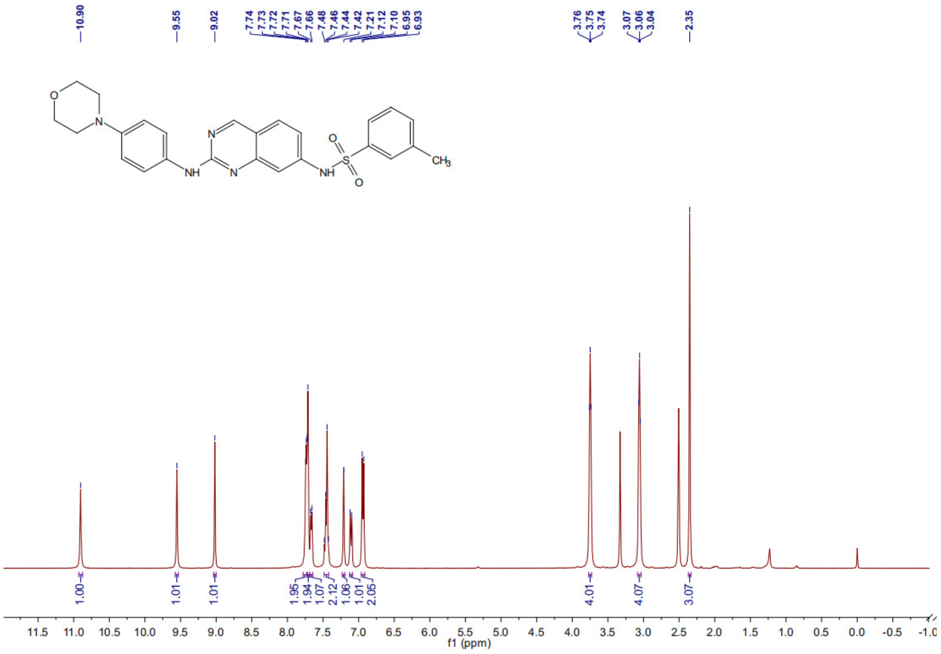

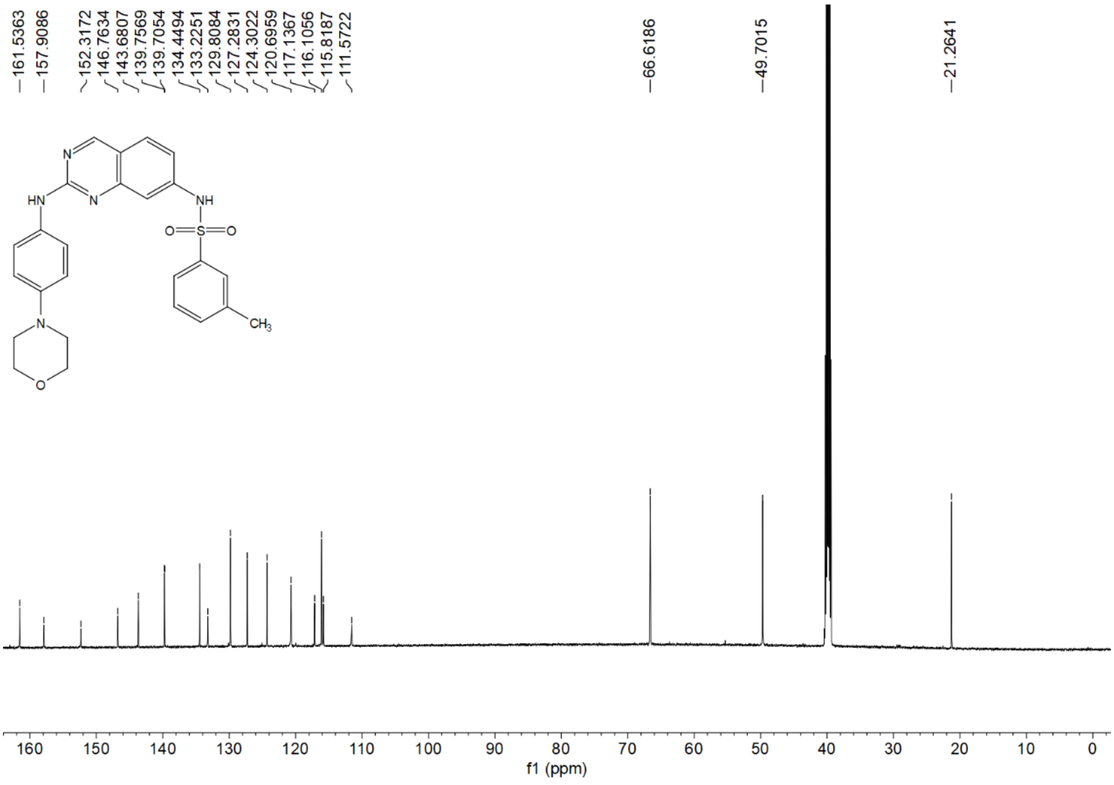

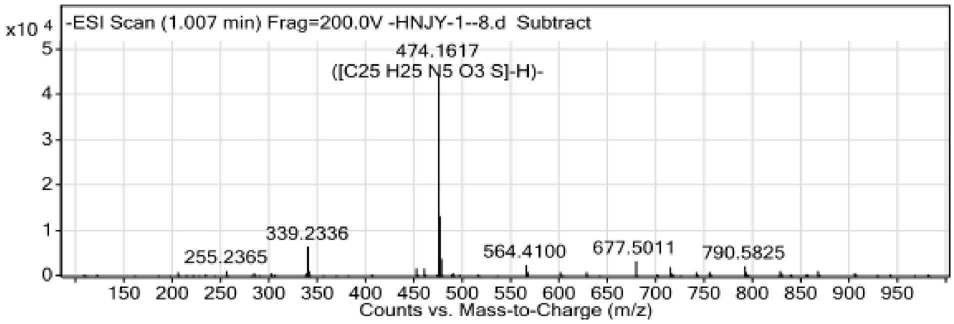

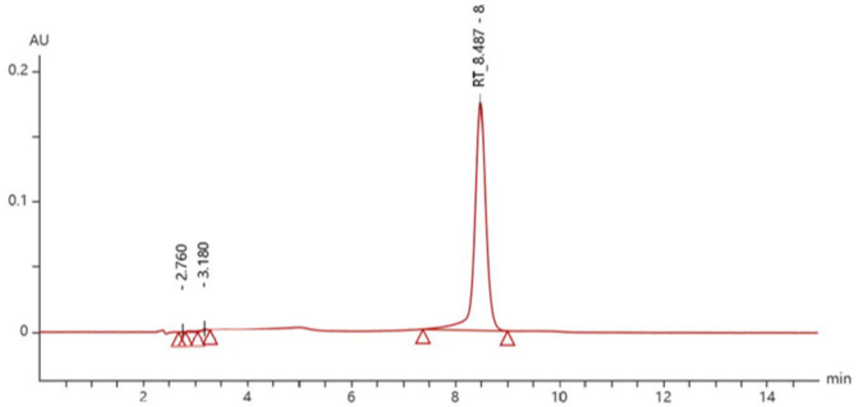

#	RT (min)	Area(μV*s)	High(μV)	Area(%)	High(%)
1	2.760	1441.633	402.850	0.054	0.227
2	3.180	9827.933	1449.806	0.370	0.818
3	4.017	-	-	-	-
4	8.487	2642995.733	175427.656	99.575	98.955

^{1}H-NMR, ^{13}C-NMR, HRMS and HPLC spectra of compound **H15**.

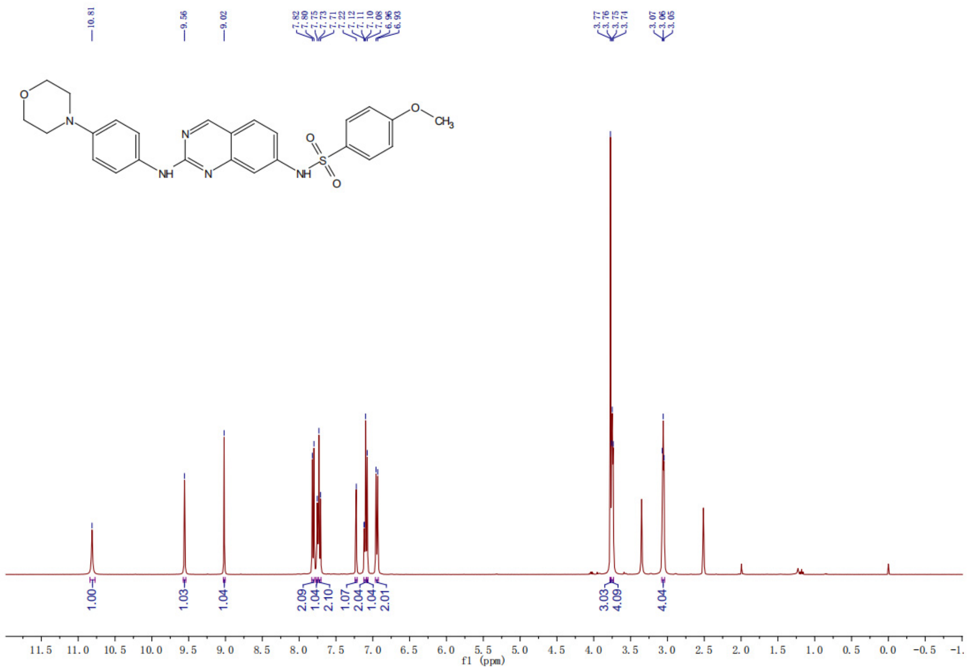

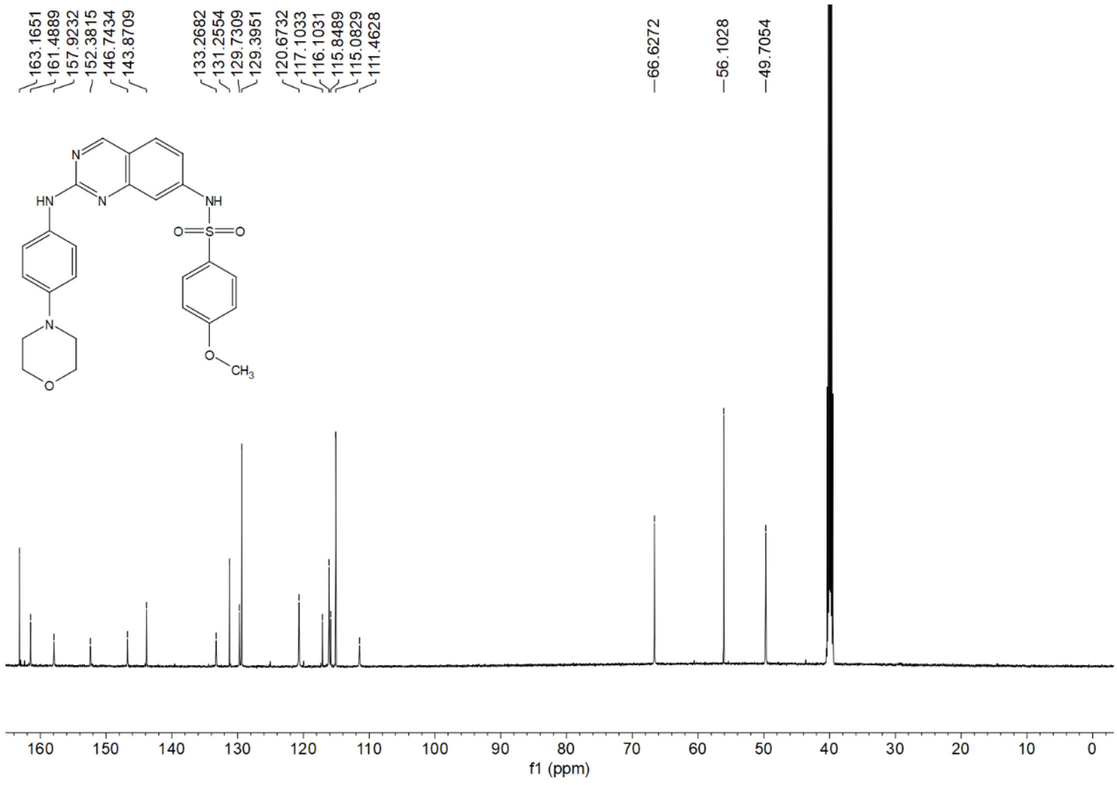

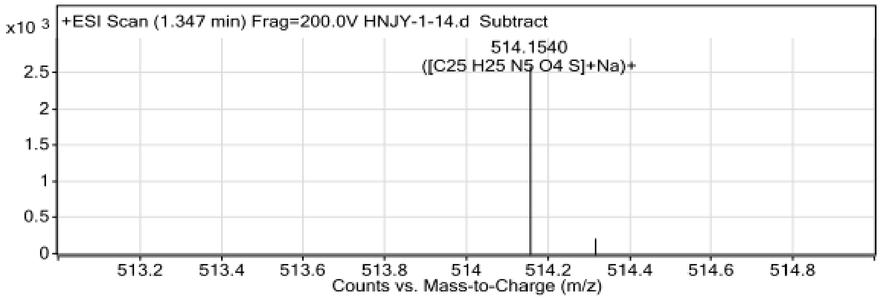

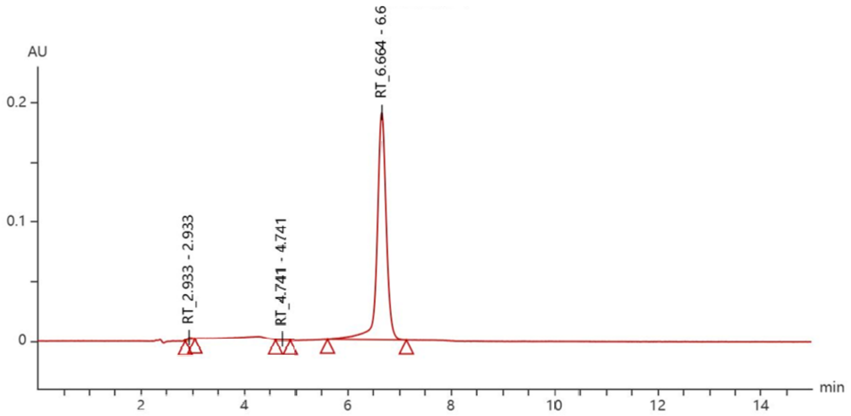

#	RT (min)	Area(μV*s)	High(μV)	Area(%)	High(%)
1	2.760	-	-	-	-
2	2.933	9719.467	1682.589	0.415	0.874
3	3.220	-	-	-	-
4	4.017	-	-	-	-
5	4.741	1156.660	-145.167	0.049	0.075
6	6.664	2333364.117	190766.261	99.536	99.051

^{1}H-NMR, ^{13}C-NMR, HRMS and HPLC spectra of compound **H16**.

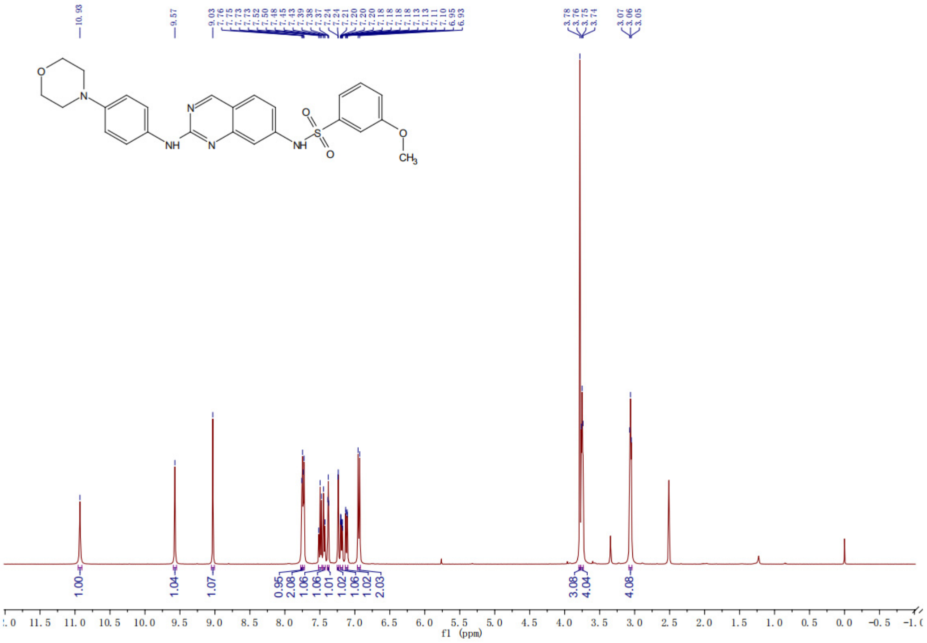

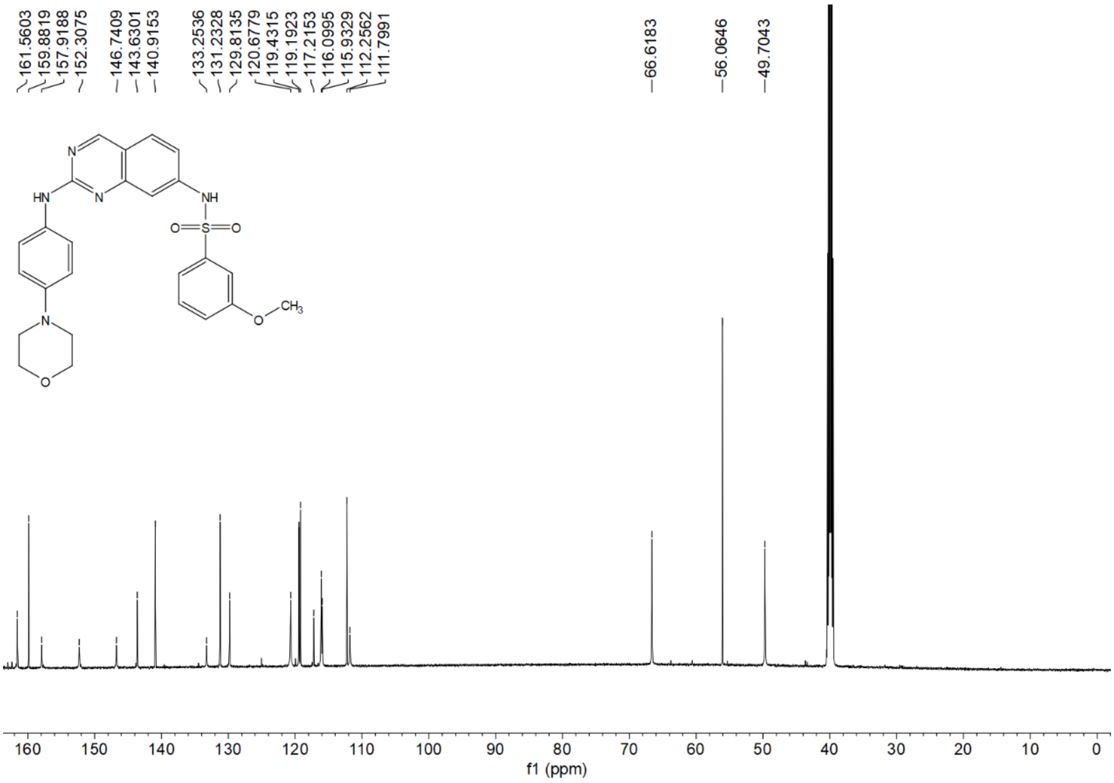

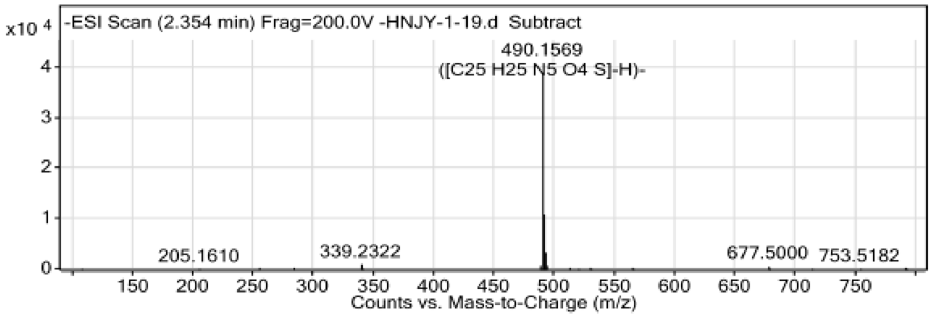

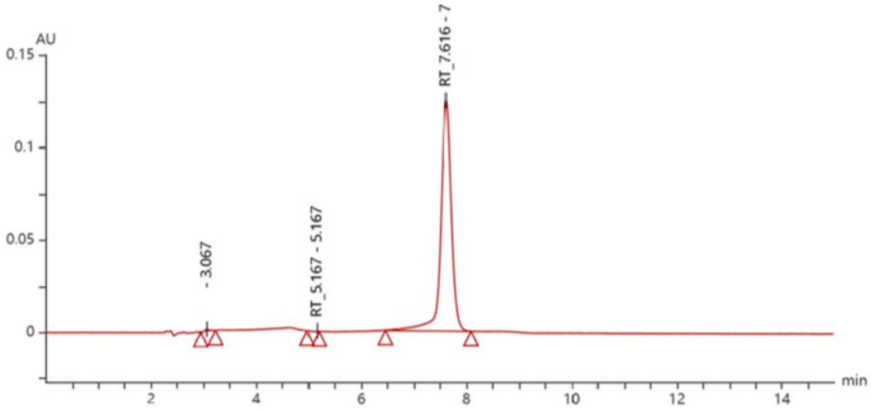

#	RT (min)	Area(μV*s)	High(μV)	Area(%)	High(%)
1	2.760	-	-	-	-
2	3.067	10332.733	1290.821	0.595	1.023
3	4.017	-	-	-	-
4	5.167	776.897	116.786	0.045	0.093
5	7.616	1726597.933	124753.809	99.361	98.884

^{1}H-NMR, ^{13}C-NMR, HRMS and HPLC spectra of compound **H17**.

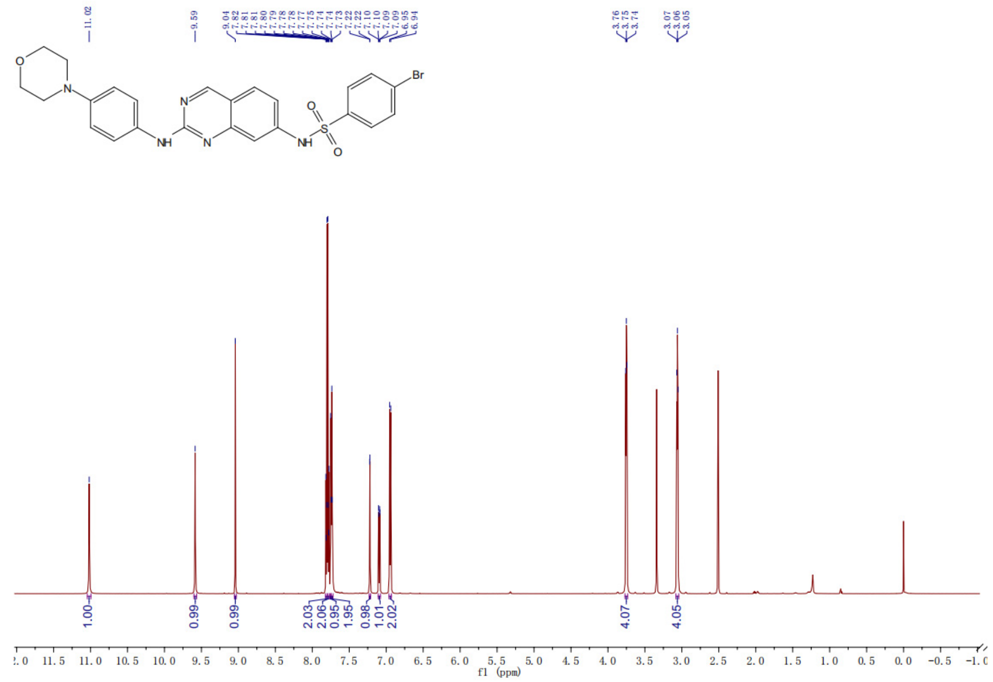

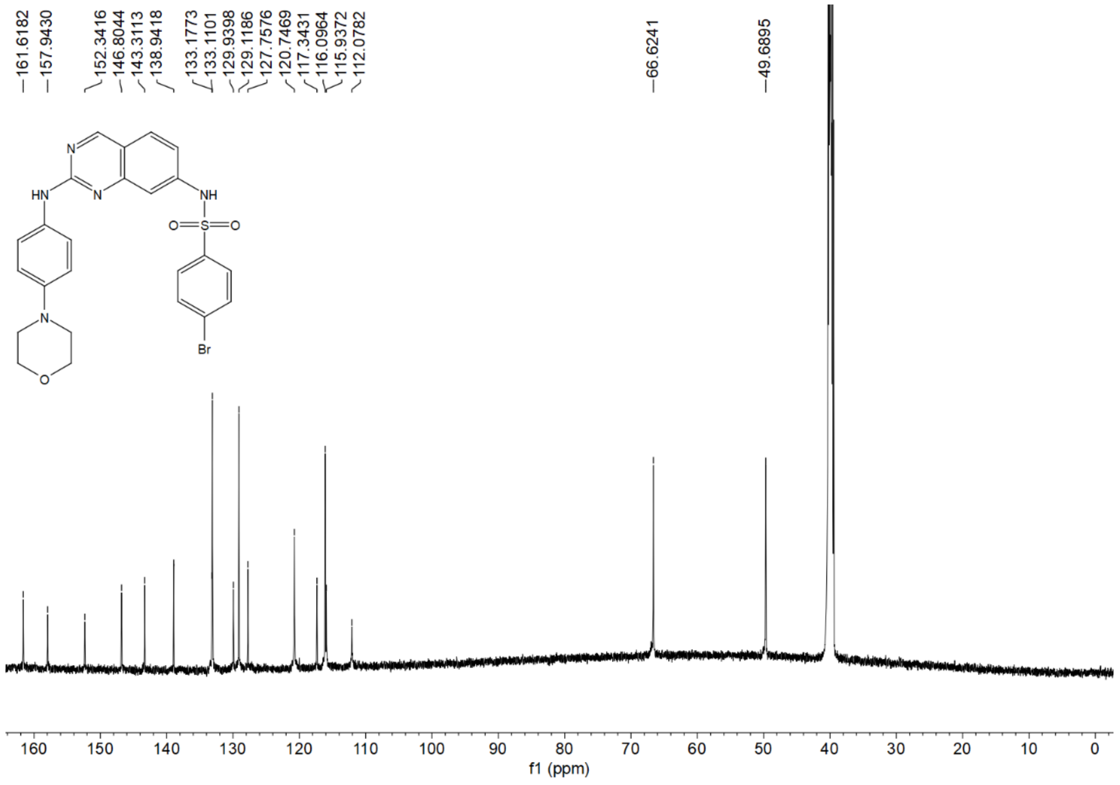

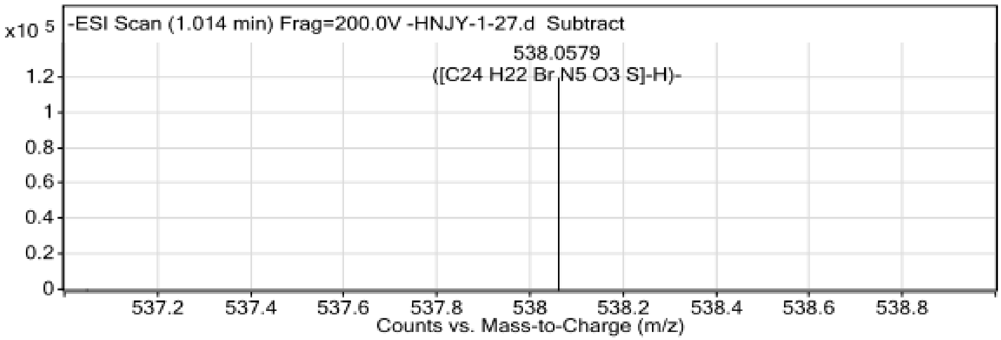

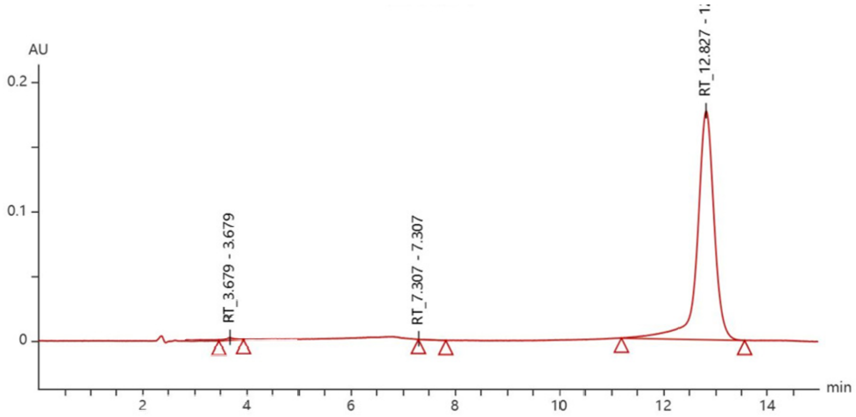

#	RT (min)	Area(μV*s)	High(μV)	Area(%)	High(%)
1	2.760	-	-	-	-
2	3.220	-	-	-	-
3	3.679	22207.133	2163.954	0.554	1.207
4	4.017	-	-	-	-
5	7.307	1815.016	213.892	0.045	0.119
6	12.827	3986989.242	176948.168	99.401	98.674

^{1}H-NMR, ^{13}C-NMR, HRMS and HPLC spectra of compound **H18**.

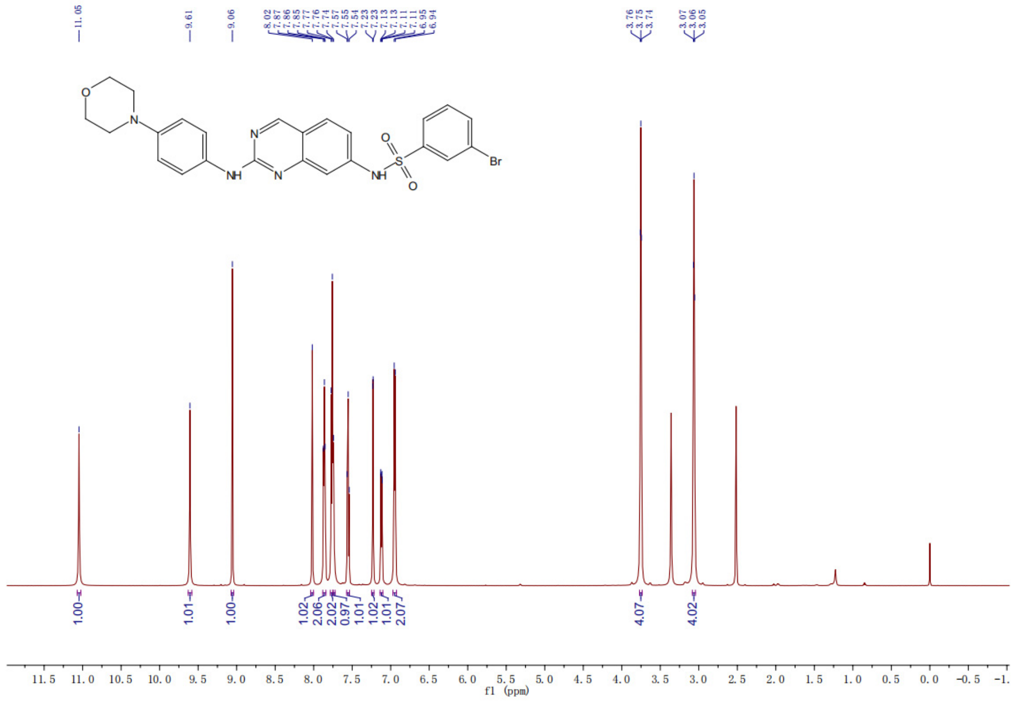

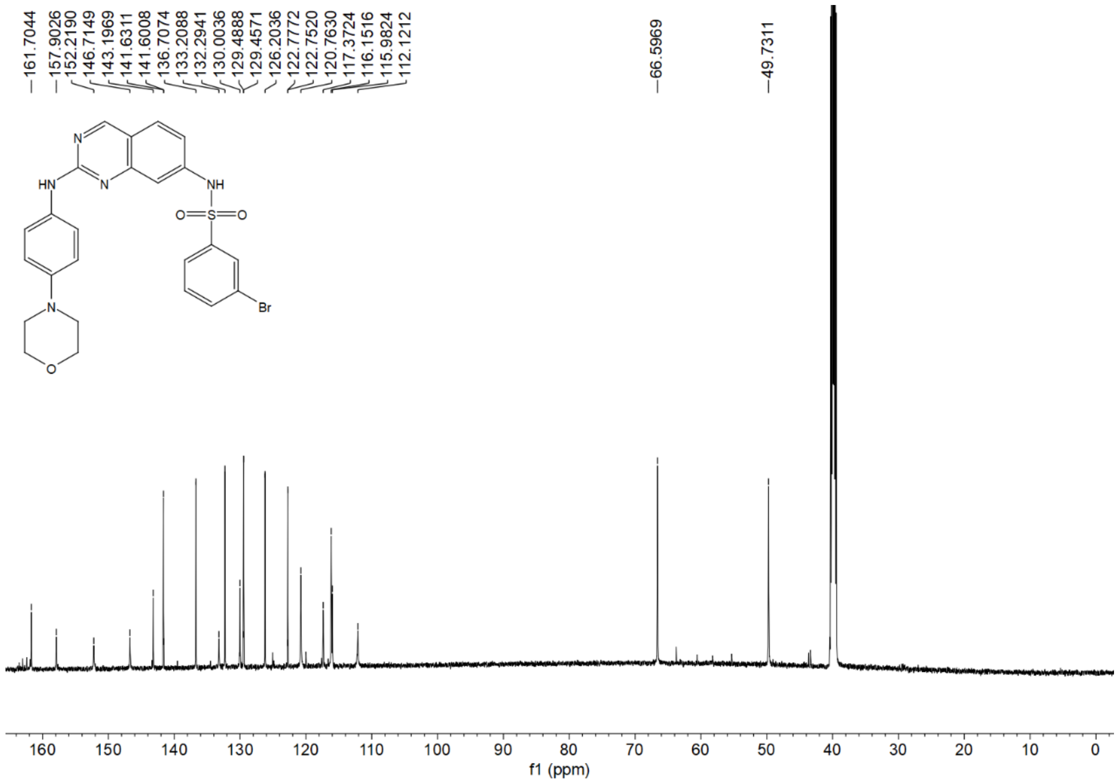

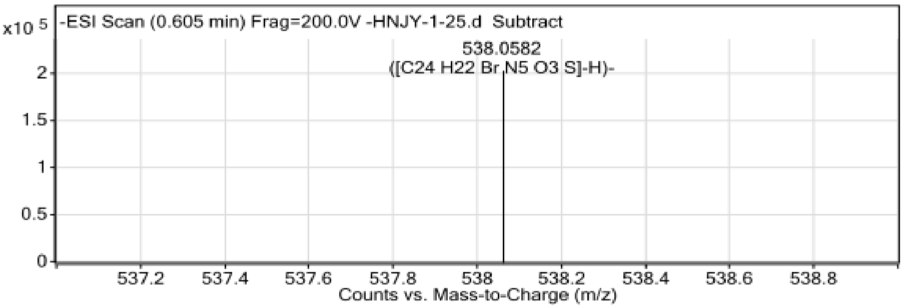

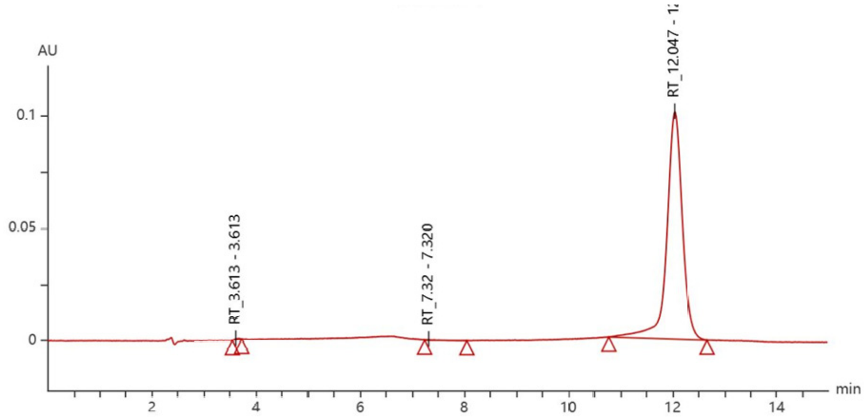

#	RT (min)	Area(μV*s)	High(μV)	Area(%)	High(%)
1	2.760	-	-	-	-
2	3.220	-	-	-	-
3	3.613	3989.133	662.352	0.190	0.650
4	4.017	-	-	-	-
5	7.320	3676.131	225.254	0.175	0.221
6	12.047	2094706.808	101079.608	99.635	99.130

^{1}H-NMR, ^{13}C-NMR, HRMS and HPLC spectra of compound **H19**.

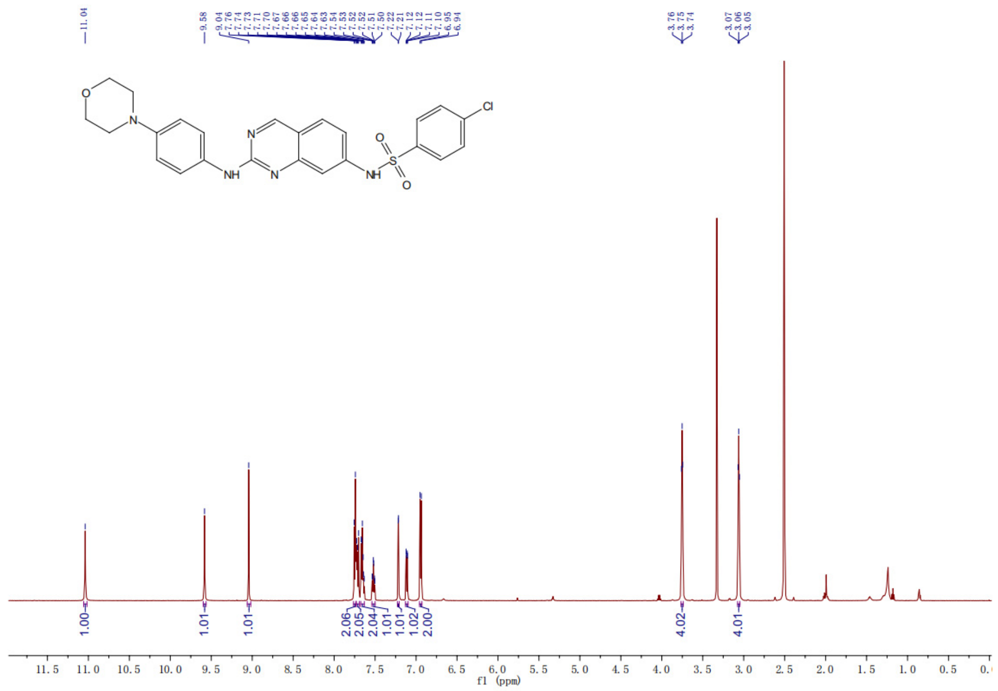

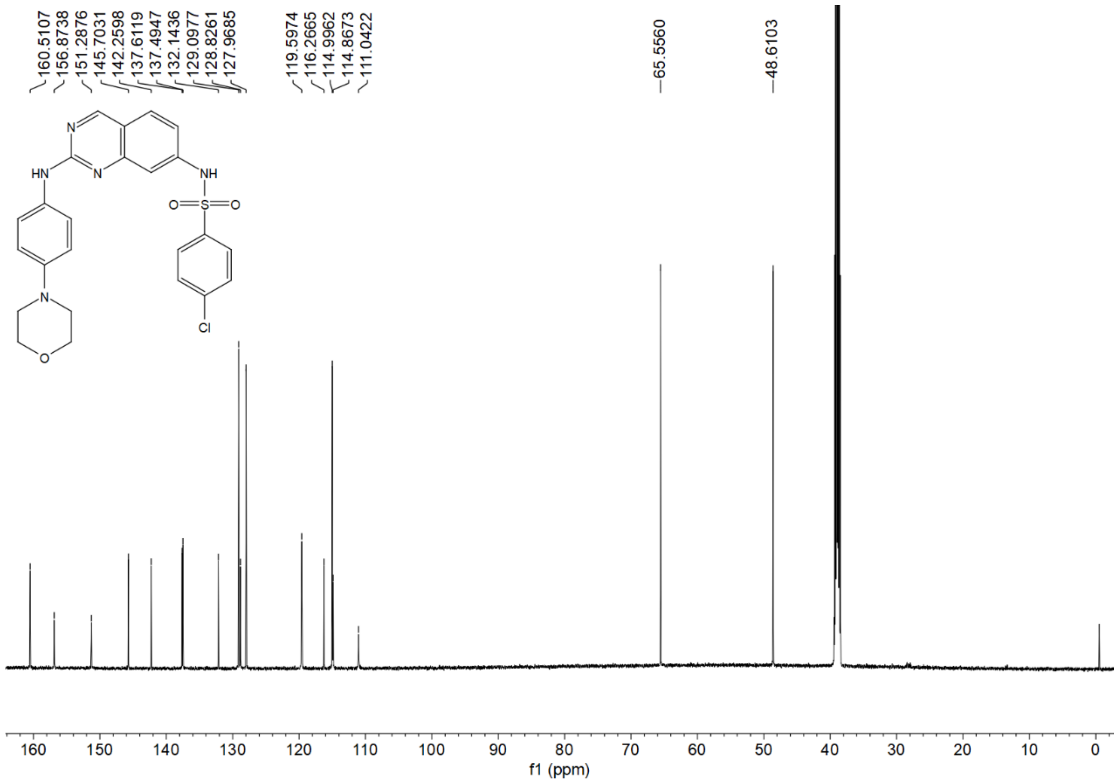

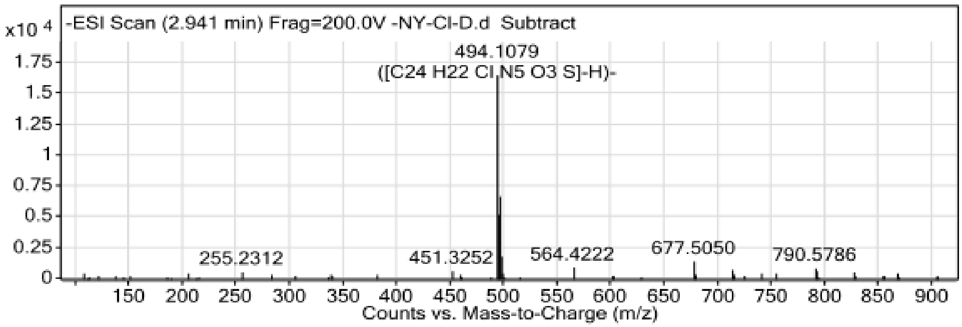

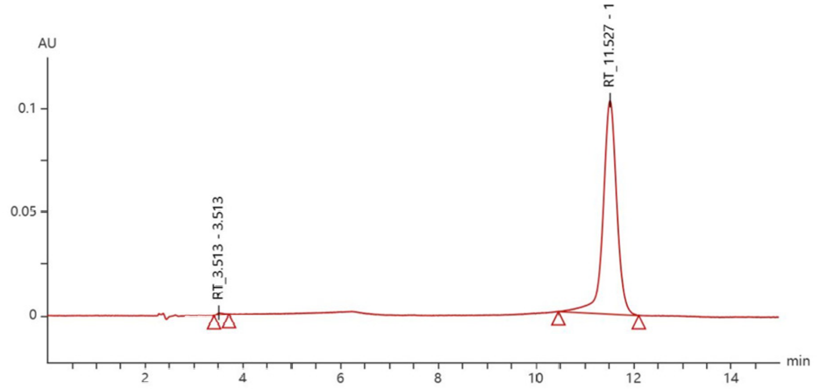

#	RT (min)	Area(μV*s)	High(μV)	Area(%)	High(%)
1	2.760	-	-	-	-
2	3.220	-	-	-	-
3	3.513	9323.933	1053.000	0.461	1.010
4	4.017	-	-	-	-
5	11.527	2014949.700	103200.415	99.539	98.990

^{1}H-NMR, ^{13}C-NMR, HRMS and HPLC spectra of compound **H20**.

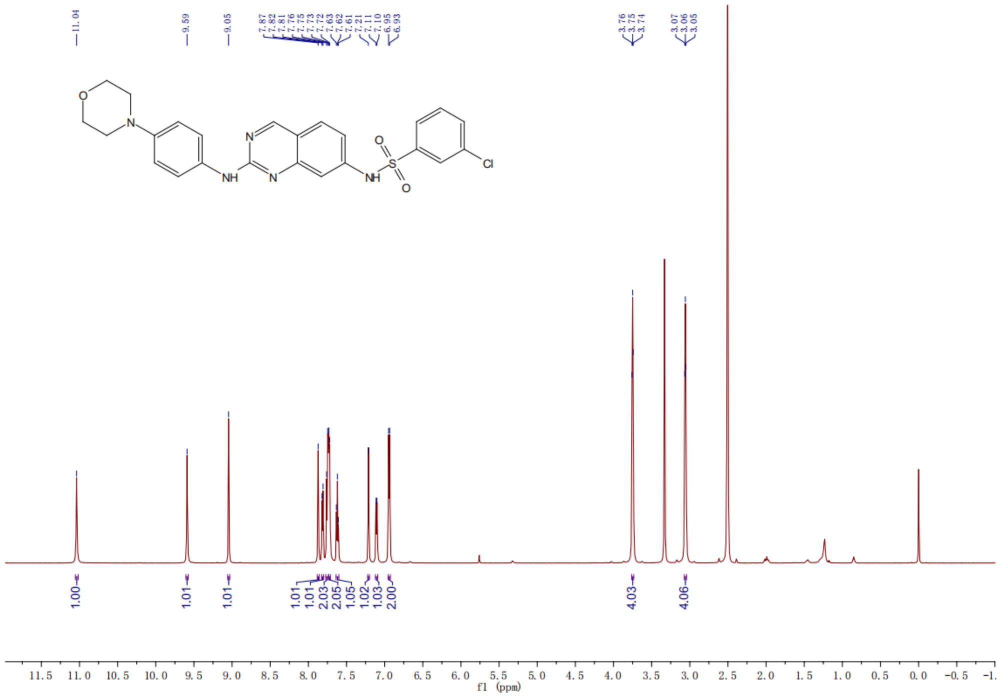

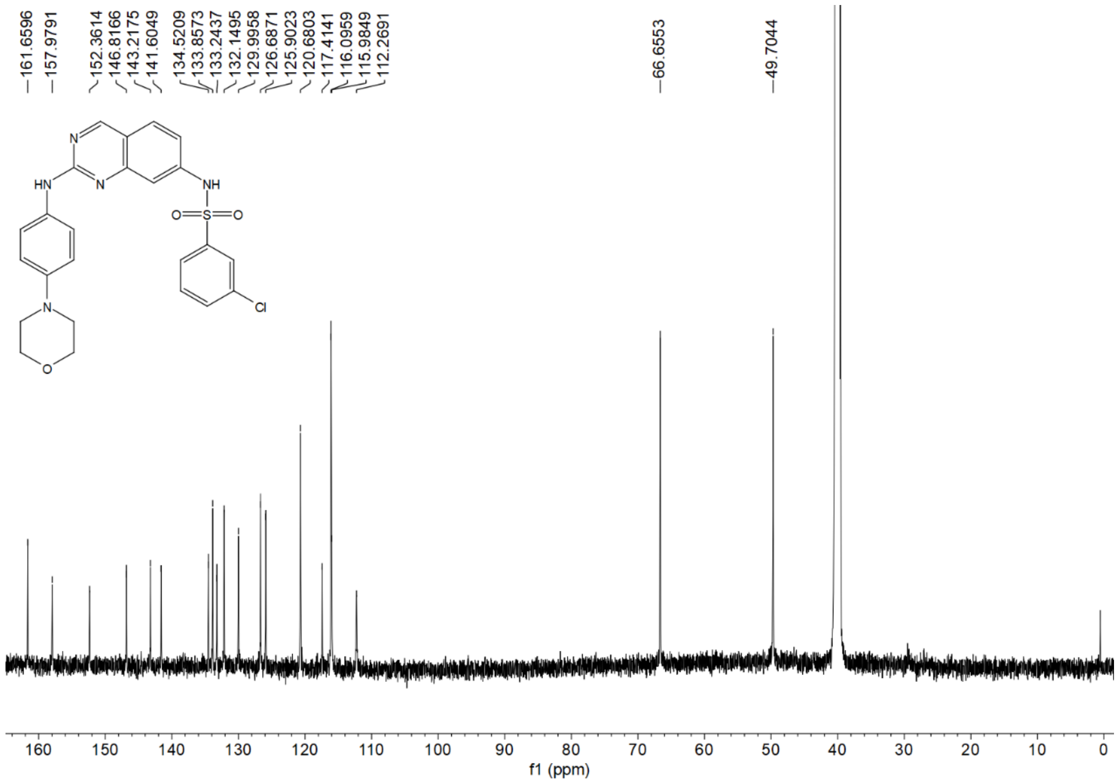

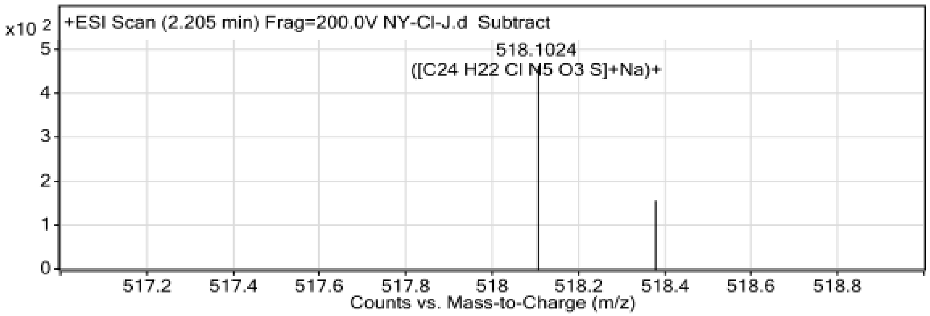

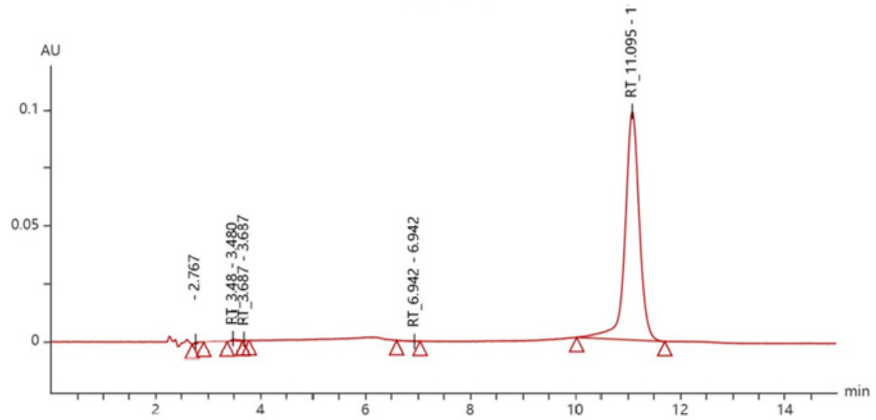

#	RT (min)	Area(μV*s)	High(μV)	Area(%)	High(%)
1	2.767	4805.033	674.439	0.256	0.670
2	3.220	-	-	-	-
3	3.480	10163.786	1030.754	0.541	1.024
4	3.687	1711.814	439.452	0.091	0.437
5	4.017	-	-	-	-
6	6.942	1599.959	-94.891	0.085	0.094
7	11.095	1860606.267	98409.934	99.027	97.775

^{1}H-NMR, ^{13}C-NMR, HRMS and HPLC spectra of compound **H21**.

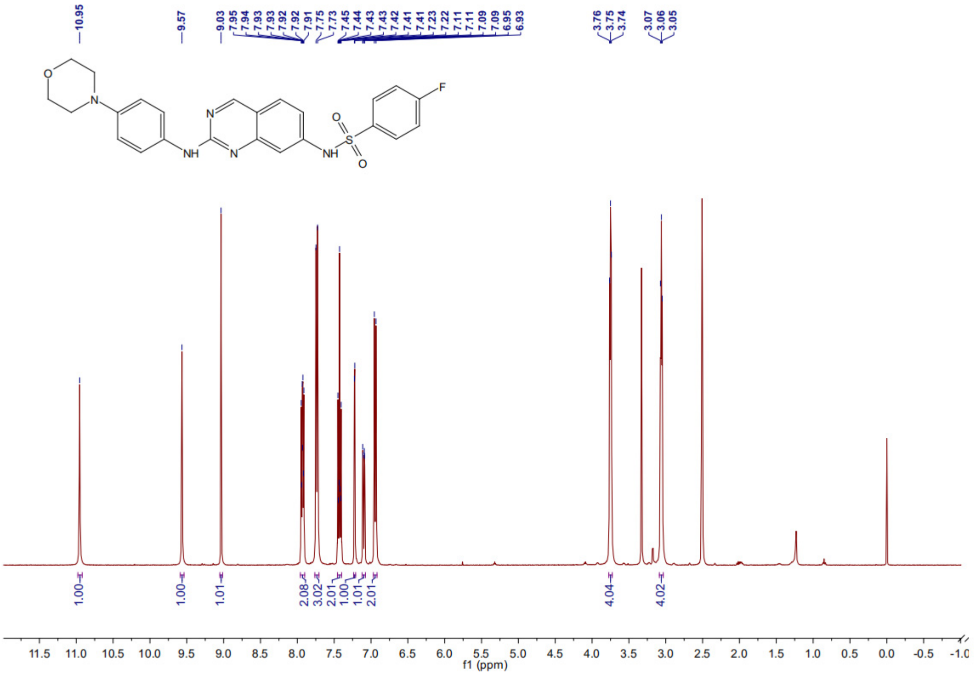

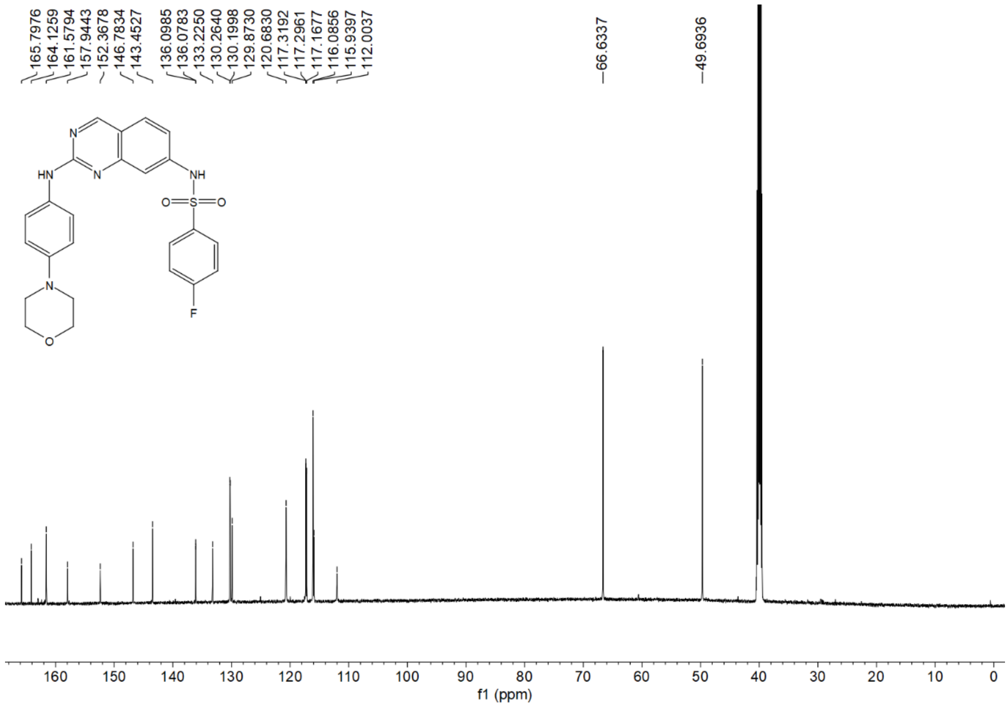

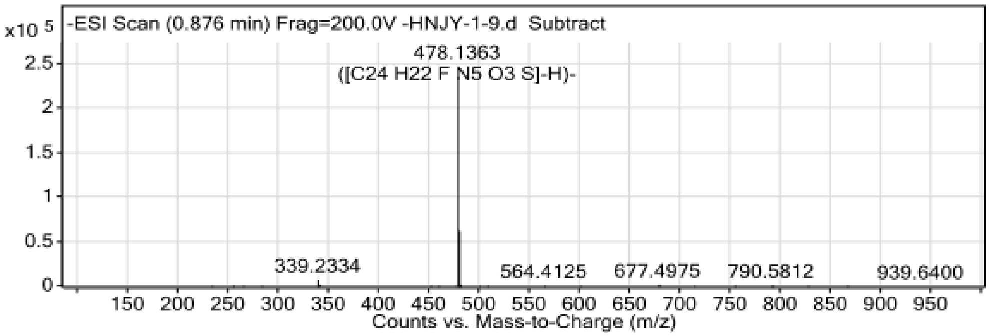

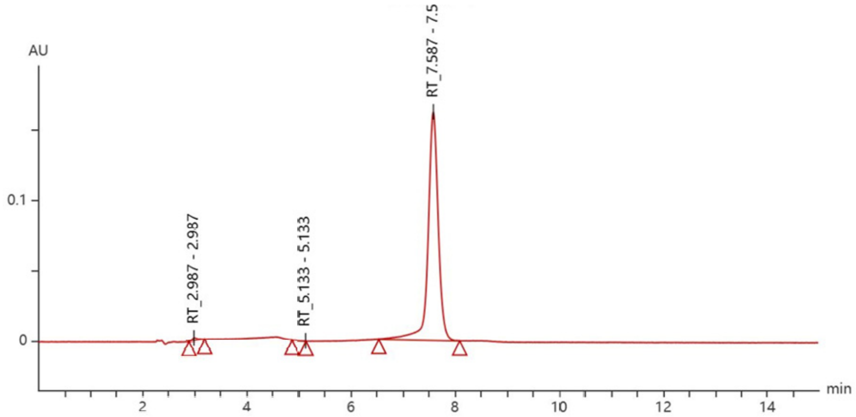

#	RT (min)	Area(μV*s)	High(μV)	Area(%)	High(%)
1	2.760	-	-	-	-
2	2.987	15792.275	2137.872	0.714	1.299
3	3.220	-	-	-	-
4	4.017	-	-	-	-
5	5.133	1359.867	198.392	0.061	0.121
6	7.587	2194404.867	162250.296	99.224	98.581

^{1}H-NMR, ^{13}C-NMR, HRMS and HPLC spectra of compound **H22**.

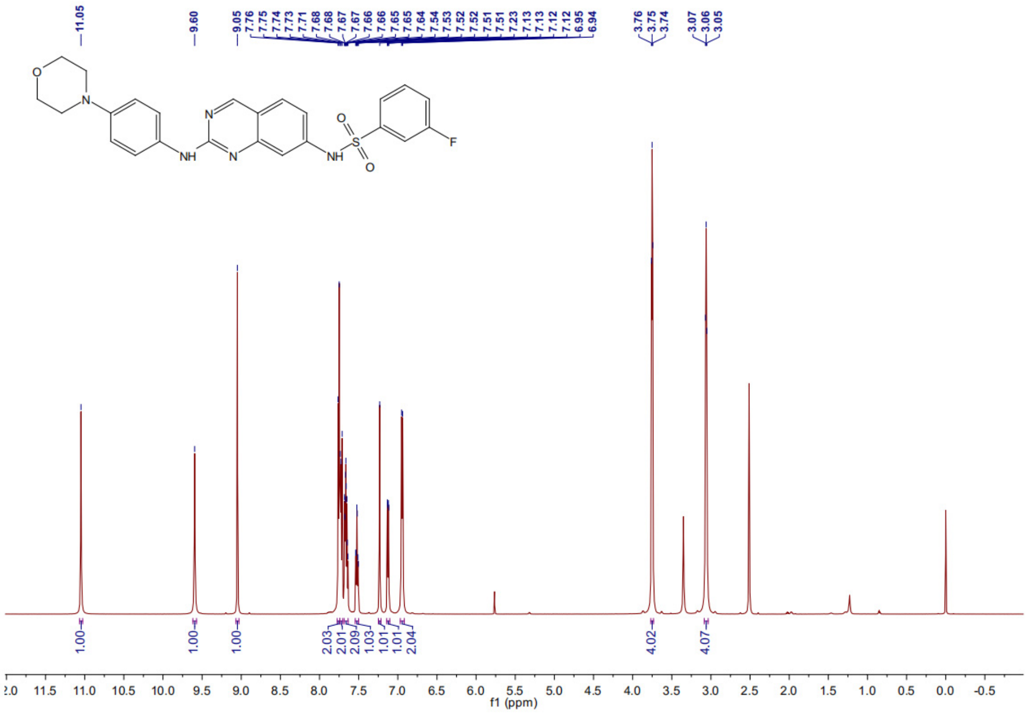

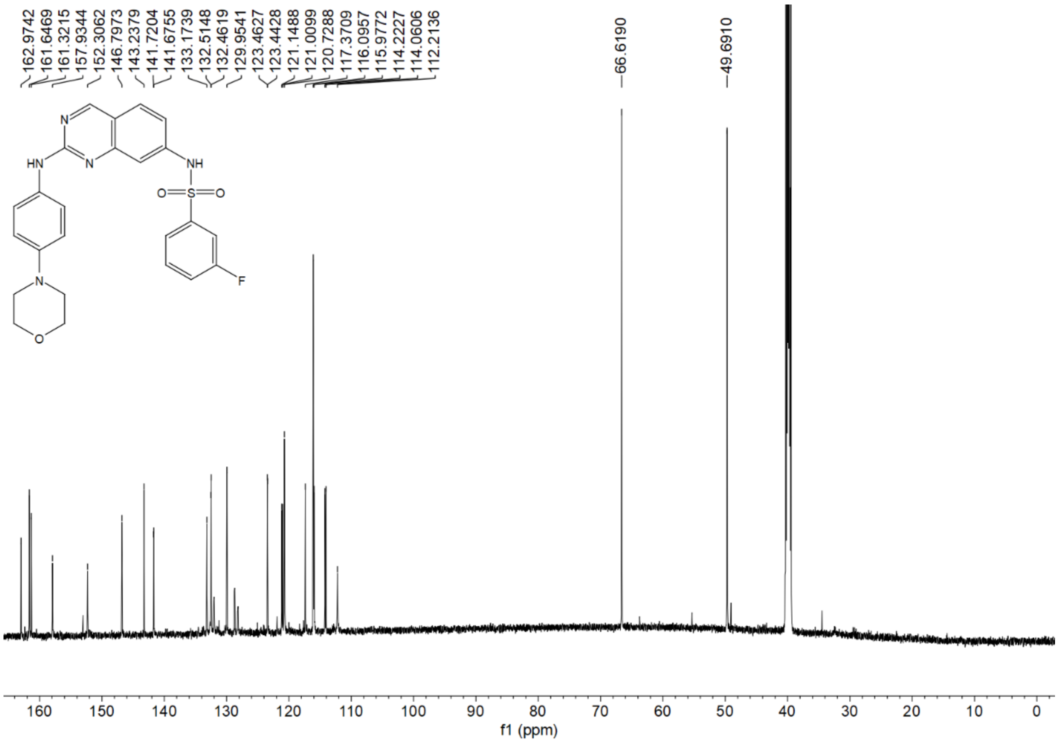

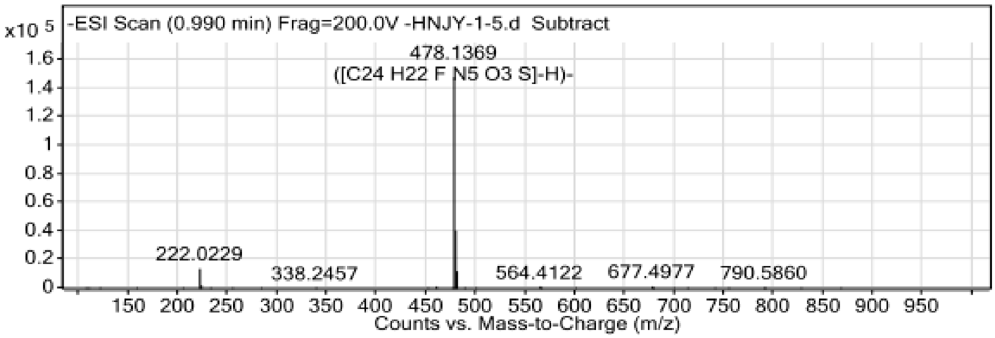

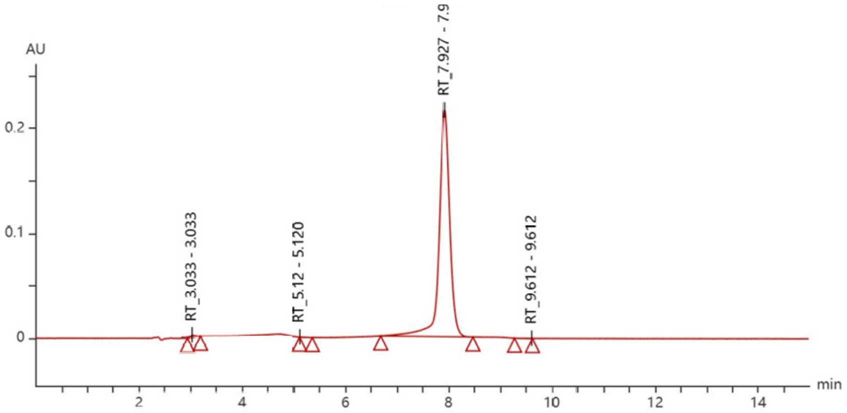

#	RT (min)	Area(μV*s)	High(μV)	Area(%)	High(%)
1	2.760	-	-	-	-
2	3.033	14323.867	1993.276	0.464	0.912
3	3.220	-	-	-	-
4	4.017	-	-	-	-
5	5.120	812.842	212.652	0.026	0.097
6	7.927	3071401.667	216140.821	99.470	98.859
7	9.612	1229.295	287.952	0.040	0.132

^{1}H-NMR, ^{13}C-NMR, HRMS and HPLC spectra of compound **H23**.

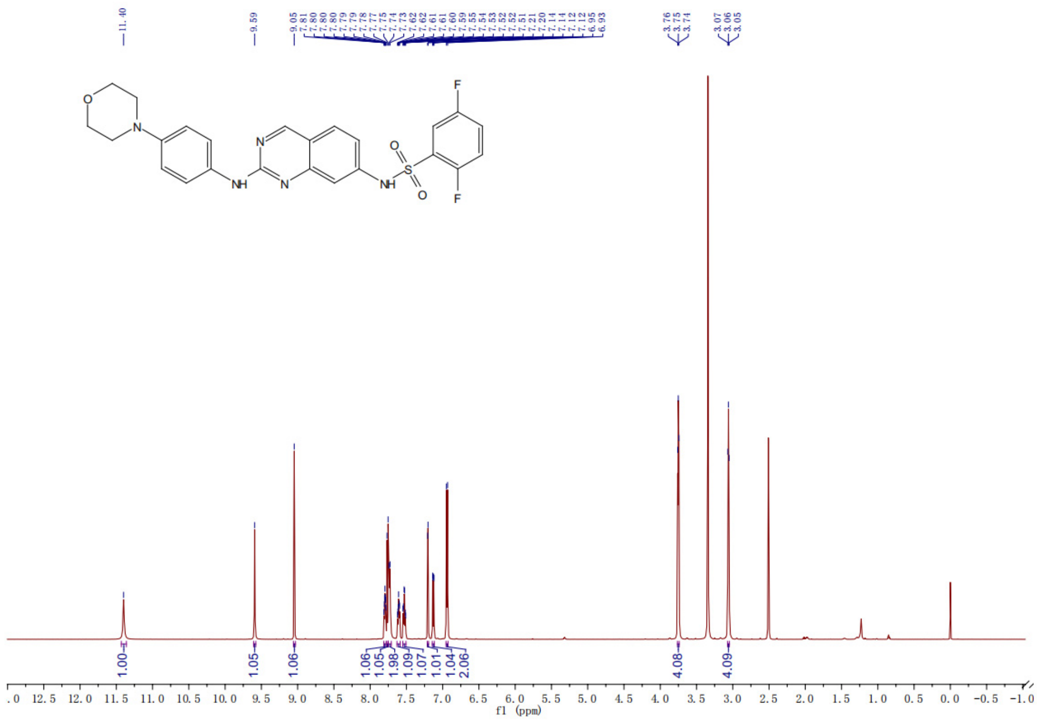

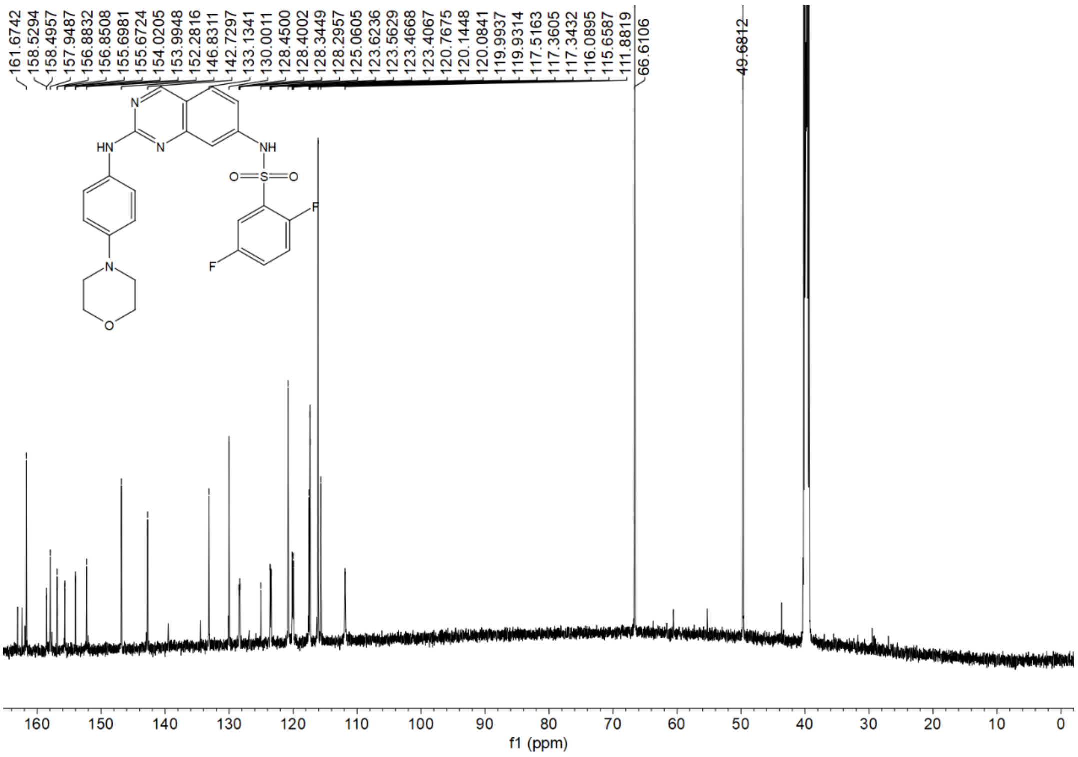

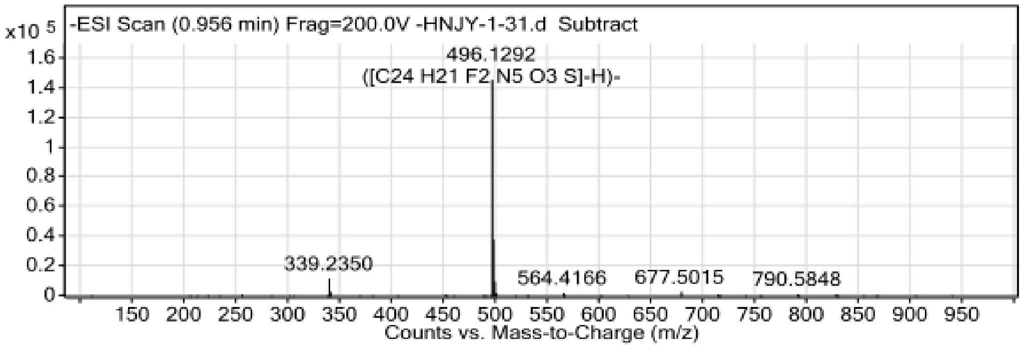

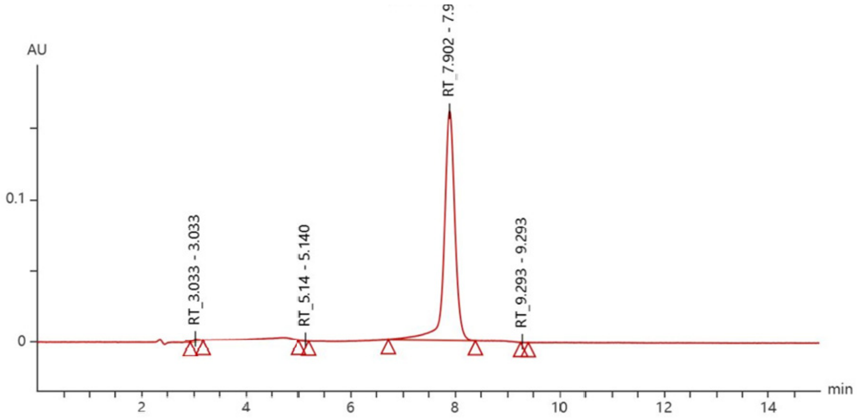

#	RT (min)	Area(μV*s)	High(μV)	Area(%)	High(%)
1	2.760	-	-	-	-
2	3.033	8727.875	1289.743	0.379	0.794
3	3.220	-	-	-	-
4	4.017	-	-	-	-
5	5.140	644.672	-23.581	0.028	0.015
6	7.902	2295334.400	160889.002	99.568	99.088
7	9.293	593.739	167.159	0.026	0.103

^{1}H-NMR, ^{13}C-NMR, HRMS and HPLC spectra of compound **H24**.

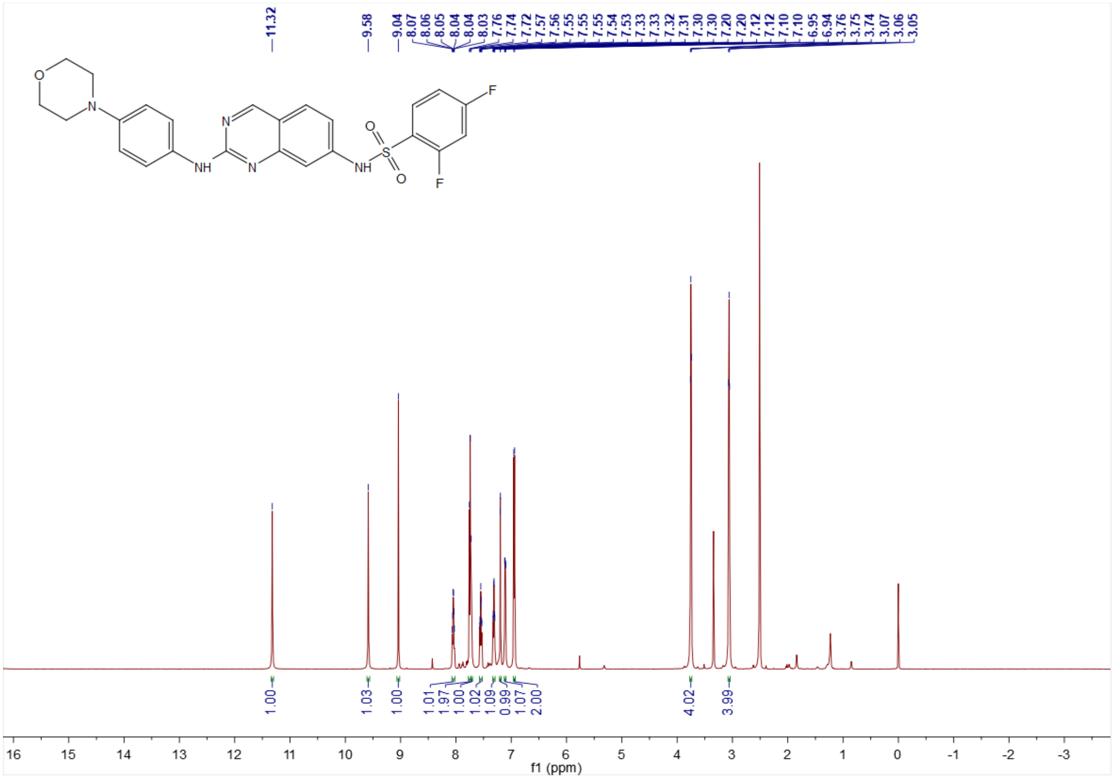

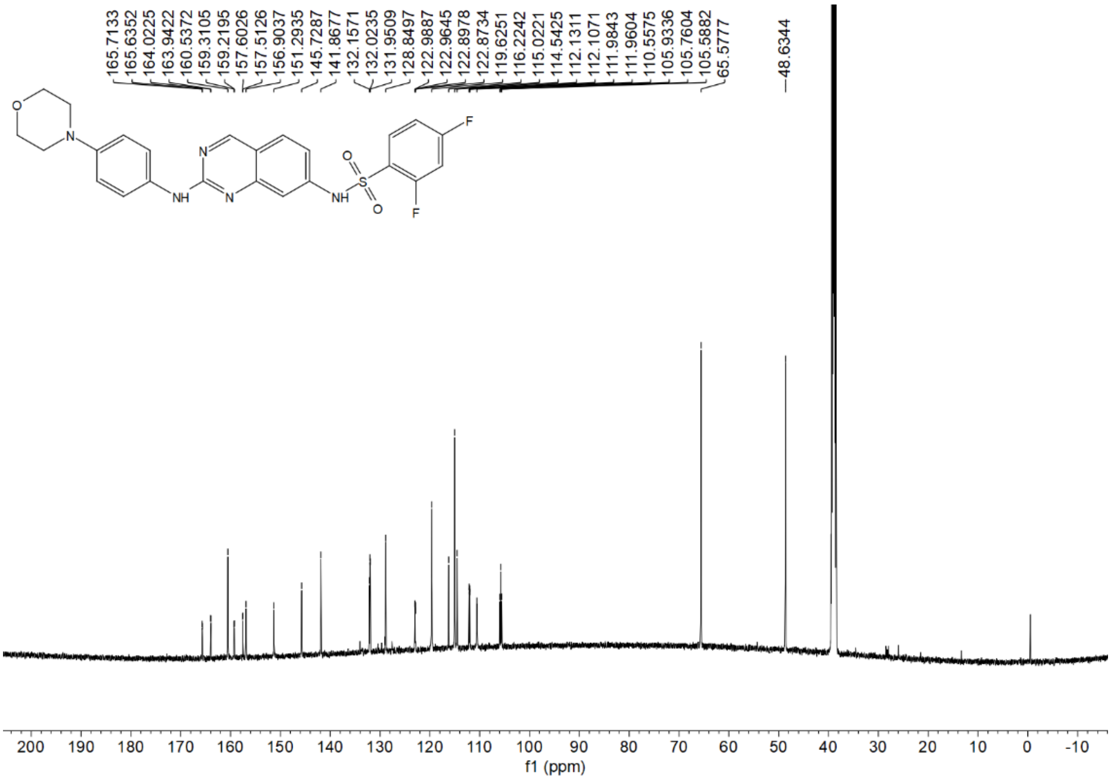

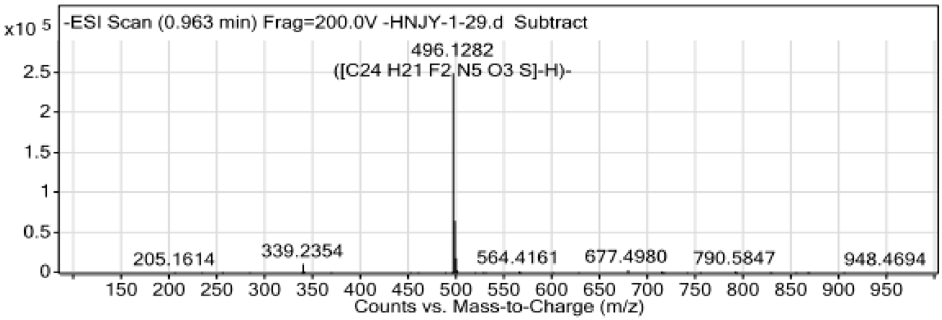

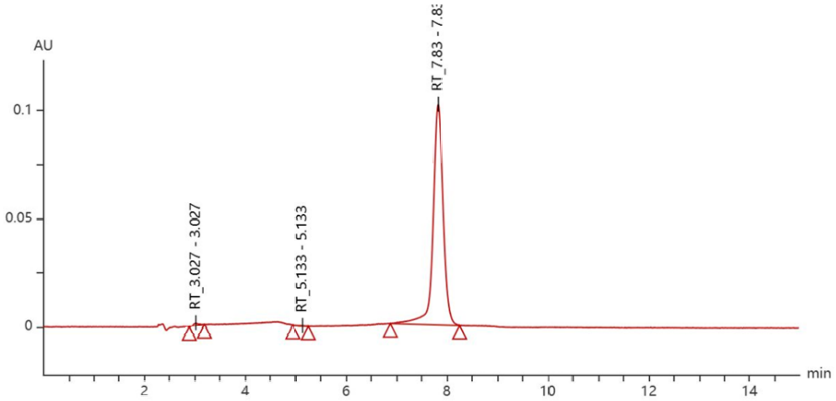

#	RT (min)	Area(μV*s)	High(μV)	Area(%)	High(%)
1	2.760	-	-	-	-
2	3.027	9246.292	1320.083	0.650	1.278
3	3.220	-	-	-	-
4	4.017	-	-	-	-
5	5.133	921.507	-76.661	0.065	0.074
6	7.830	1411248.200	101858.197	99.285	98.647

^{1}H-NMR, ^{13}C-NMR, HRMS and HPLC spectra of compound **H25**.

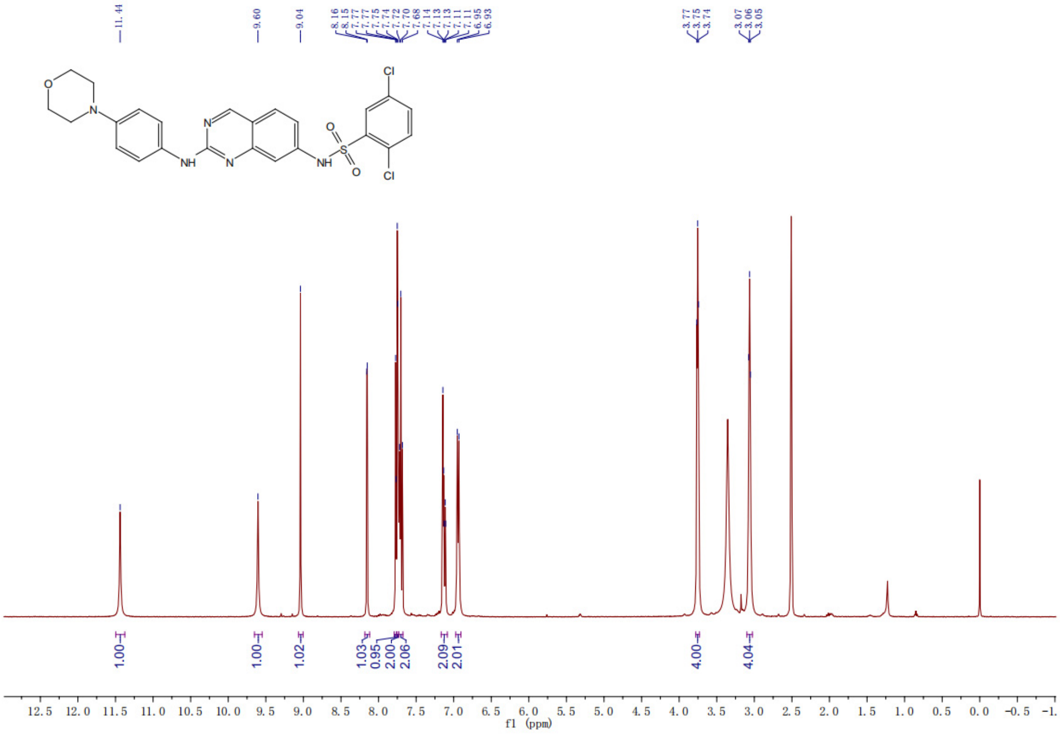

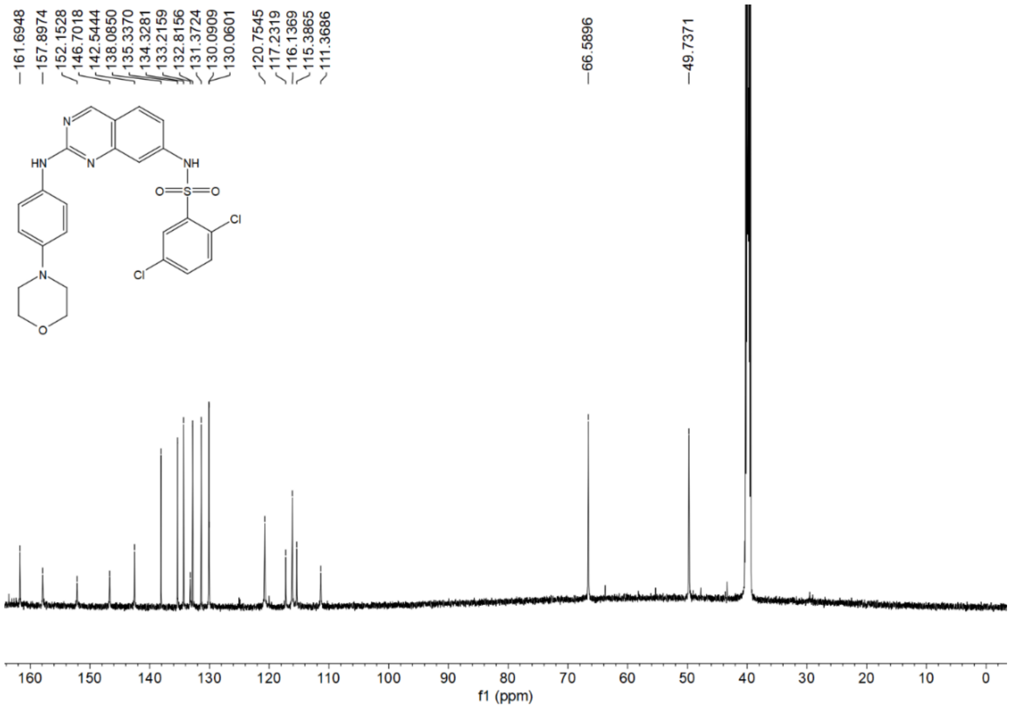

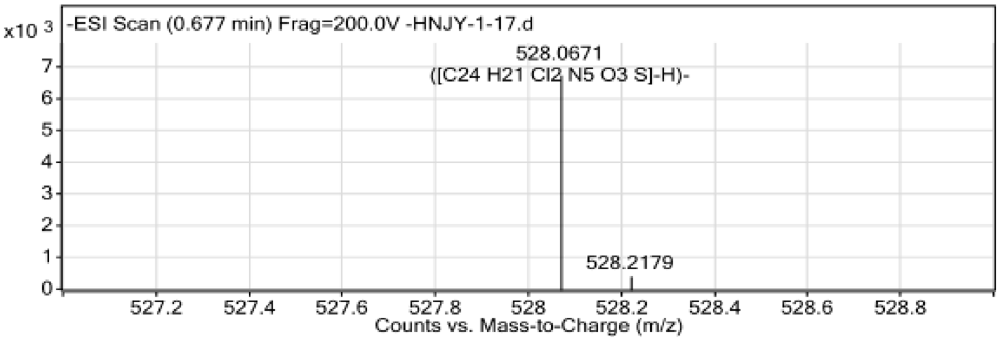

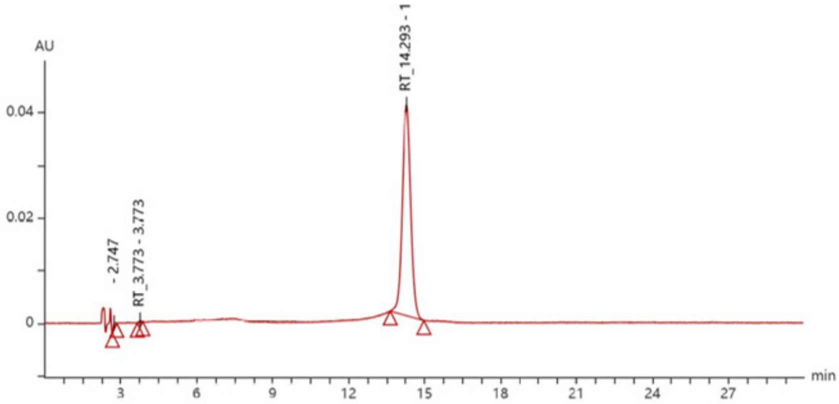

#	RT (min)	Area(μV*s)	High(μV)	Area(%)	High(%)
1	2.747	6602.200	1145.712	0.719	2.757
2	3.220	-	-	-	-
3	3.773	2501.500	350.538	0.273	0.843
4	4.017	-	-	-	-
5	14.293	908607.142	40062.368	99.008	96.400

^{1}H-NMR, ^{13}C-NMR, HRMS and HPLC spectra of compound **H26**.

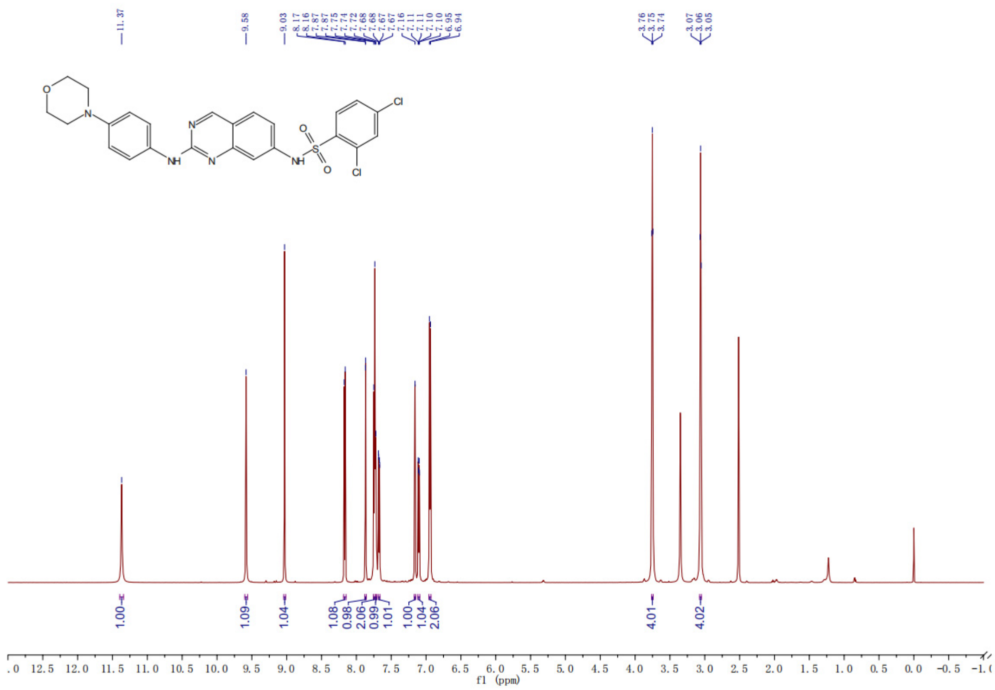

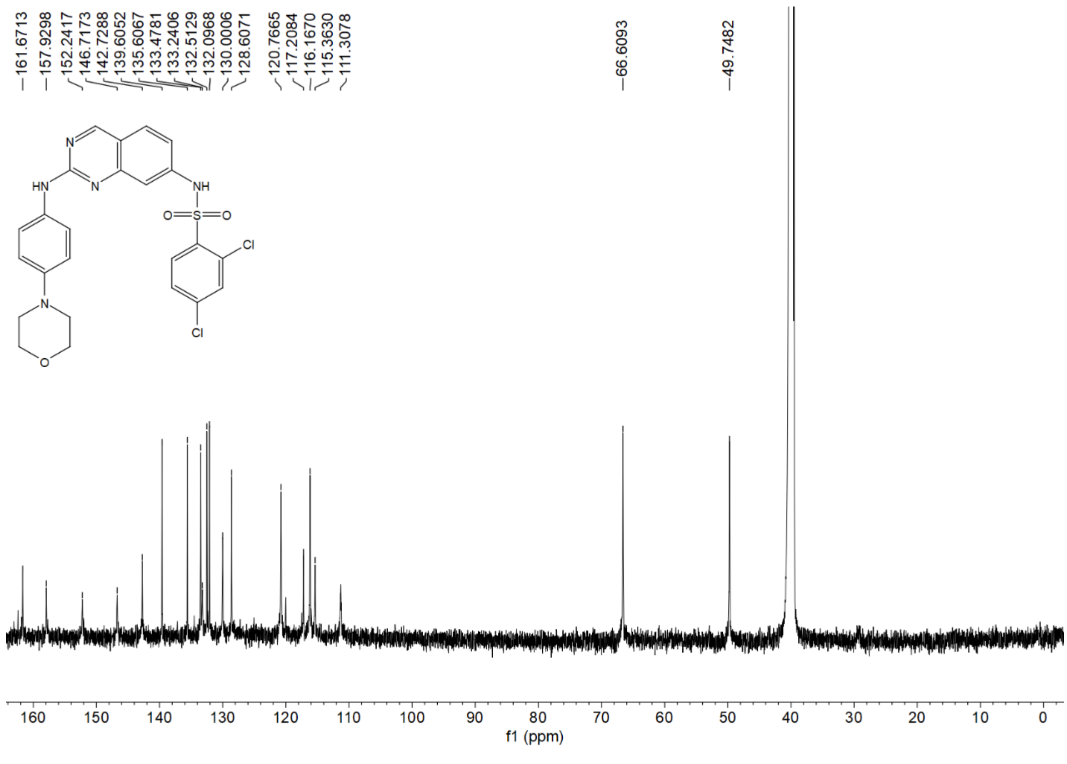

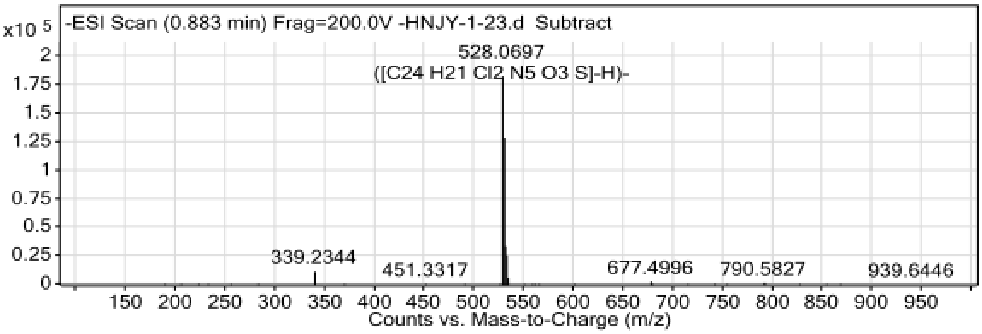

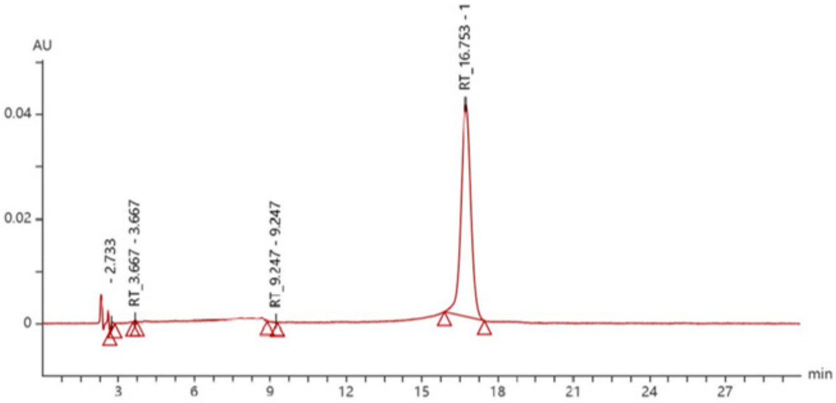

#	RT (min)	Area(μV*s)	High(μV)	Area(%)	High(%)
1	2.733	7689.267	1062.833	0.707	2.527
2	3.220	-	-	-	-
3	3.667	2166.067	369.096	0.199	0.877
4	4.017	-	-	-	-
5	9.247	1115.492	140.803	0.102	0.335
6	16.753	1077356.808	40493.412	98.992	96.261

^{1}H-NMR, ^{13}C-NMR, HRMS and HPLC spectra of compound **H27**.

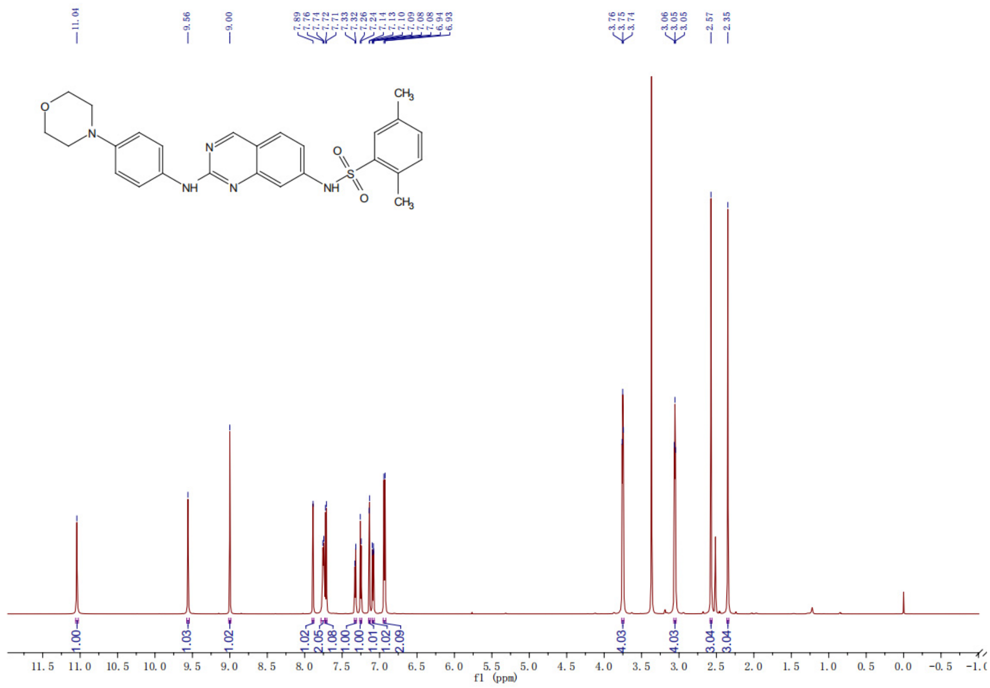

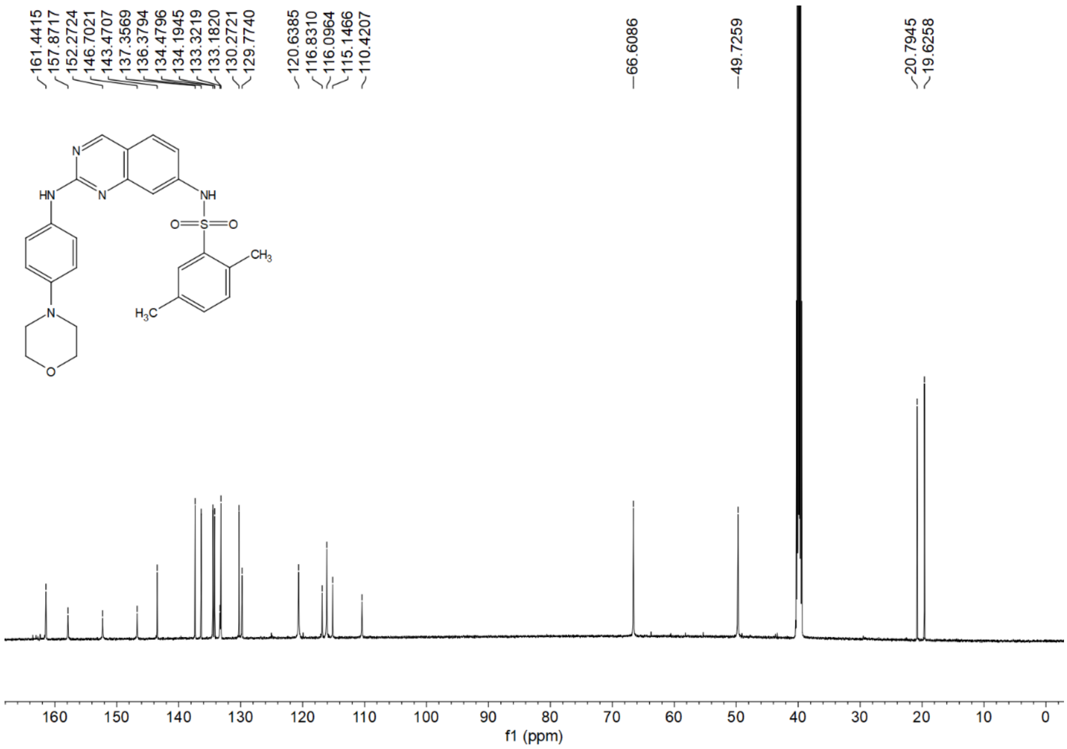

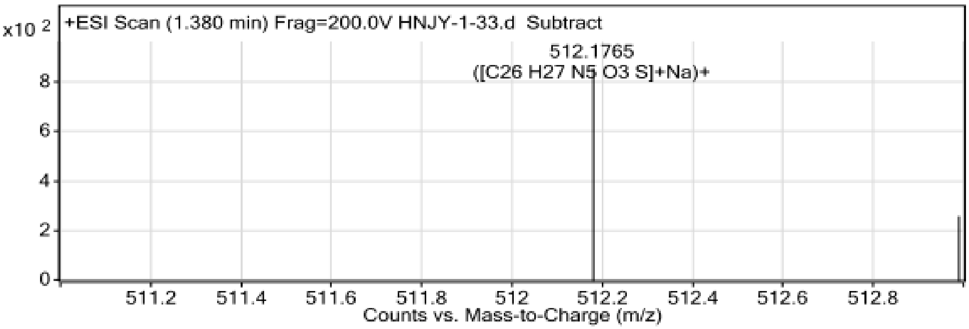

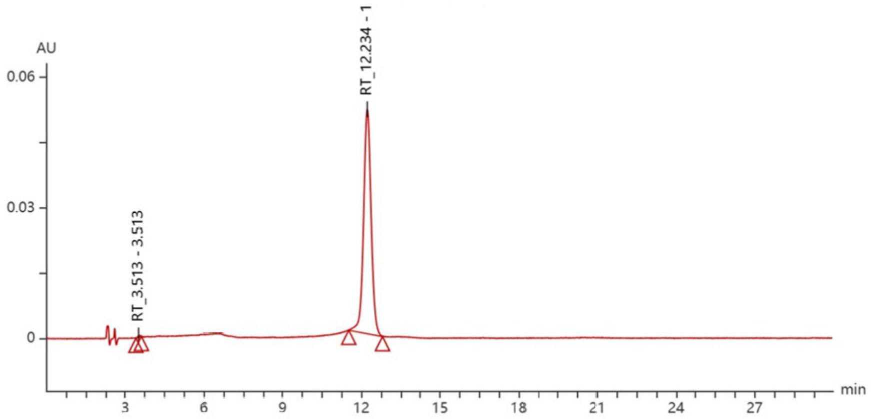

#	RT (min)	Area(μV*s)	High(μV)	Area(%)	High(%)
1	2.760	-	-	-	-
2	3.220	-	-	-	-
3	3.513	2426.168	386.944	0.235	0.746
4	4.017	-	-	-	-
5	12.234	1029533.550	51473.289	99.765	99.254

^{1}H-NMR, ^{13}C-NMR, HRMS and HPLC spectra of compound **H28**.

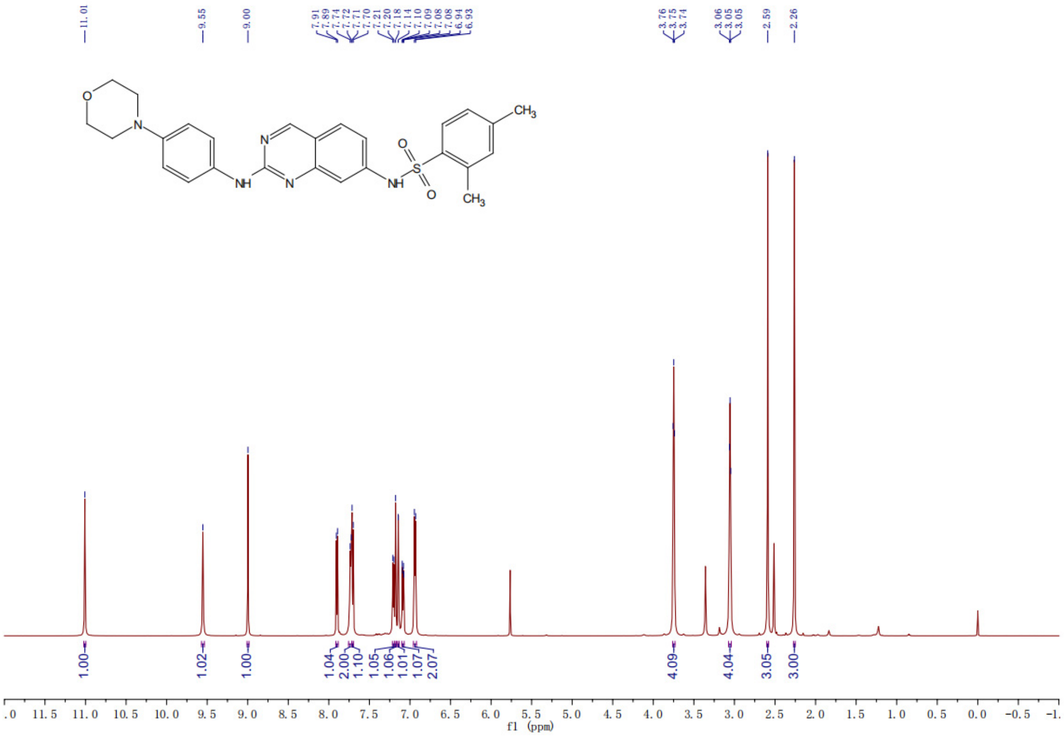

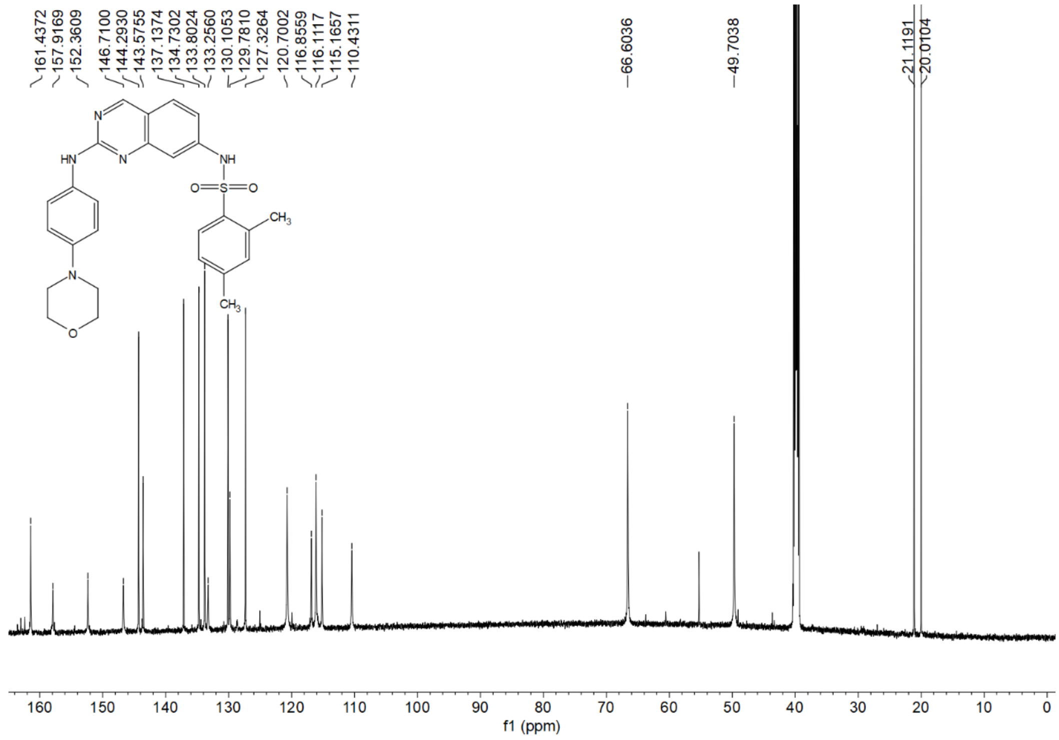

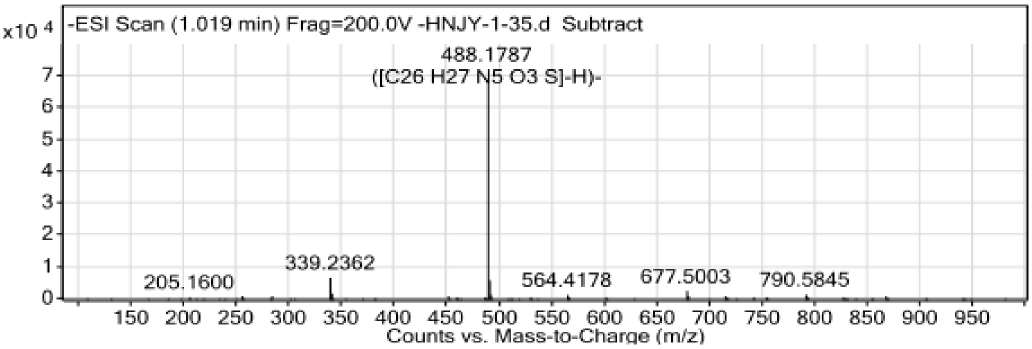

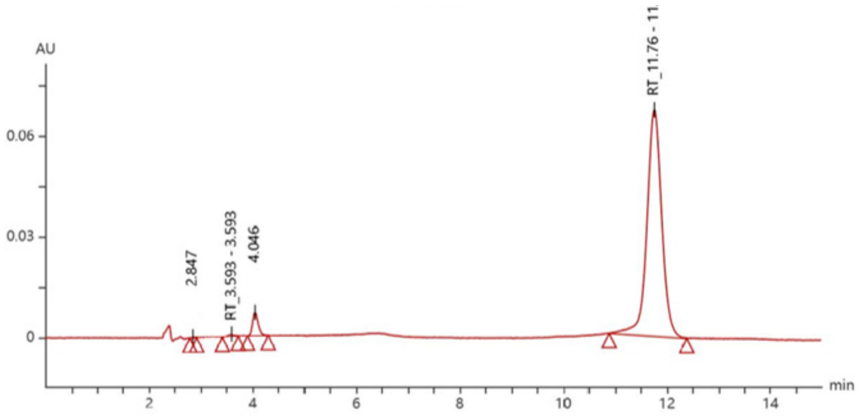

#	RT (min)	Area(μV*s)	High(μV)	Area(%)	High(%)
1	2.847	1715.667	352.050	0.122	0.466
2	3.220	-	-	-	-
3	3.593	6581.400	776.283	0.469	1.028
4	4.046	53918.633	6897.179	3.840	9.133
5	11.760	1341761.850	67492.804	95.569	89.373
